# A simple protocol for the synthesis of perylene bisimides from perylene tetracarboxylic dianhydride†

**DOI:** 10.1039/d4ra01576b

**Published:** 2024-04-08

**Authors:** Elina Marinho, Pedro R. Figueiredo, Rui Araújo, M. Fernanda Proença

**Affiliations:** a Department of Chemistry, University of Minho Campus de Gualtar 4710-057 Braga Portugal fproenca@quimica.uminho.pt +351 253604379

## Abstract

Perylene bisimides are highly attractive polycyclic aromatic hydrocarbons due to their photostability associated to unique and characteristic photochemical properties. They have been widely used for analytical purposes, despite the hydrophobicity of most of these compounds. The ring substitution pattern plays an important role in fine-tuning the physicochemical properties that govern solubility and aggregation. In this work, a selection of perylene bisimides were prepared from the reaction of perylenetetracarboxylic dianhydride with α-amino acids or primary aliphatic and aromatic amines. These molecules were obtained in good yield by a simple synthetic protocol based on the use of imidazole as a green solvent and avoiding the need for complex purification methods, a major advantage for future applications. Functionalization of the exocyclic substituent can also be performed and was exemplified by the incorporation of the maleimide and anthraquinone moieties.

## Abstract 2

PBIs were synthesized from the reaction of perylene bisanhydrides and primary amines in imidazole as solvent. Maleimide, anthraquinone or ferrocene were bonded to the side chain and the *S*-trityl group was replaced by imidazole in *S*-trityl cysteine.

## Graphical abstract

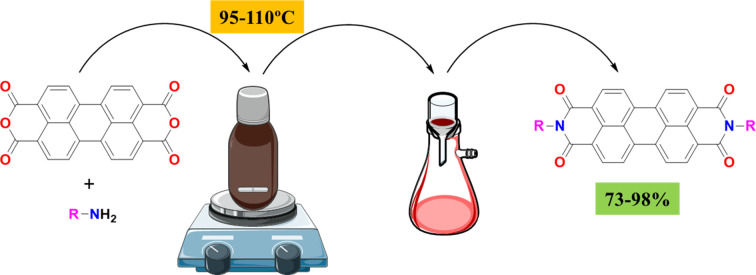

## 1. Introduction

**Fig. 1:**
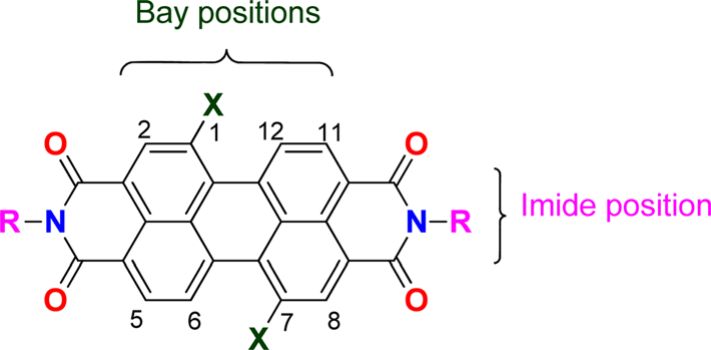
Structure of PBI and numbering of the aromatic carbons in the periphery of the ring system.

Perylene bisimides (PBI, Fig. 1) are valuable functional materials that have been used for several applications, depending on the substitution pattern of the fused aromatic ring. A wide range of substituents can be incorporated in the imide group or in the bay positions (C1, C6, C7 or C12).^1^ PBIs functionalized in the *ortho* positions (C2, C5, C8 or C11), have only been available in the past ten years, opening new and interesting research directions for PBI chemistry.^2^ These compounds have been extensively investigated because of their outstanding chemical, physical and optoelectronic properties, as well as their stability under thermal, oxidative stress and high electron mobility.^3^ They have been mostly used as semiconducting materials in organic solar cells,^1,4^ field effect transistors,^5^ light emitting diodes^6^ and hybrid LEDs^7^ due to their extended π-conjugation coupled with HOMO–LUMO suitable values, supplying emission properties along with efficient charge transfer.^8^

PBIs have also been used as typical fluorescent probes mostly in biological or environmental studies, as they are easy to prepare and can be modified in different ring positions, showing also good light, heat and chemical stability.^2^ Compounds incorporating the PBI nucleus also presented anticancer, antifungal, antiviral, antidepressant and antibacterial activities.^9^

According to the literature, PBIs were obtained by condensation of perylene tetracarboxylic acid dianhydride (PTCDA) with a primary amine function^10^ including amino acids^8,10*d*,11^ at high temperatures in solvents such as molten imidazole,^8,10*d*,11*c*,12^ quinolone^7^ or ethylene glycol.^12*d*,13^ Zinc acetate^10*c*,*d*,13*b*,14^ and zinc chloride catalysts^12*c*,14*a*,15^ were also used to increase the reactivity of aromatic amines.

The non-covalent functionalization of carbon nanotubes using PBIs has been successfully applied within our research group. This mild and effective functionalization reaction allowed the chemical modification of the carbon nanotube surface without affecting the external carbon layer. Varying the substituent at the imide function of the PBI unit proved to be a fundamental asset to control the selective interaction with the neighboring environment or the desired properties of the material. A simple and efficient synthetic method was previously developed in-house for the preparation of a selection of PBI, from the reaction of perylenetetracarboxylic dianhydride with α-amino acids^16^ and this line of research has been further explored.

In this work, we report the synthesis of PBIs from the reaction of PTCDA with primary amines, extending the scope of this method to other amino acids and to primary aliphatic and aromatic amines and hydrazines. The reaction conditions were optimized in order to enhance the sustainability of the synthetic protocol, reducing waste, avoiding aggressive solvents and minimizing the temperature.

## 2. Results and discussion

The reaction of perylenetetracarboxylic dianhydride 1 with amino acids was initially performed with phenylalanine 2a (2–2.1 eq.) using imidazole (10–17 eq.) as solvent and heating at 95 °C and 110 °C for 2 h and 4 h respectively. The milder experimental conditions resulted in the best isolated yield of PBI 3a (91%) and a temperature of 95 °C, allowing the solid imidazole to melt, was selected for the reactions with the different amino acids (conditions summarized in Table 1, entries 1–8). Imidazole is considered a green solvent and was previously used in the synthesis of PBIs by reaction of perylenetetracarboxylic dianhydride with 2–2.2 eq. of an amino acid (alanine, valine, phenylalanine, isoleucine, leucine, histidine, cysteine or aspartic acid) and heating at 120–140 °C for 0.5–8 h.^8,10*d*,11*c*,12^ The reaction was performed under argon^12*a*^ or nitrogen^11*c*^ atmosphere or in the absence of an inert gas.^8,10*d*^ The use of zinc acetate as catalyst was also reported.^10*d*^

**Table 1:** Optimized experimental conditions for the synthesis of PBIs 3

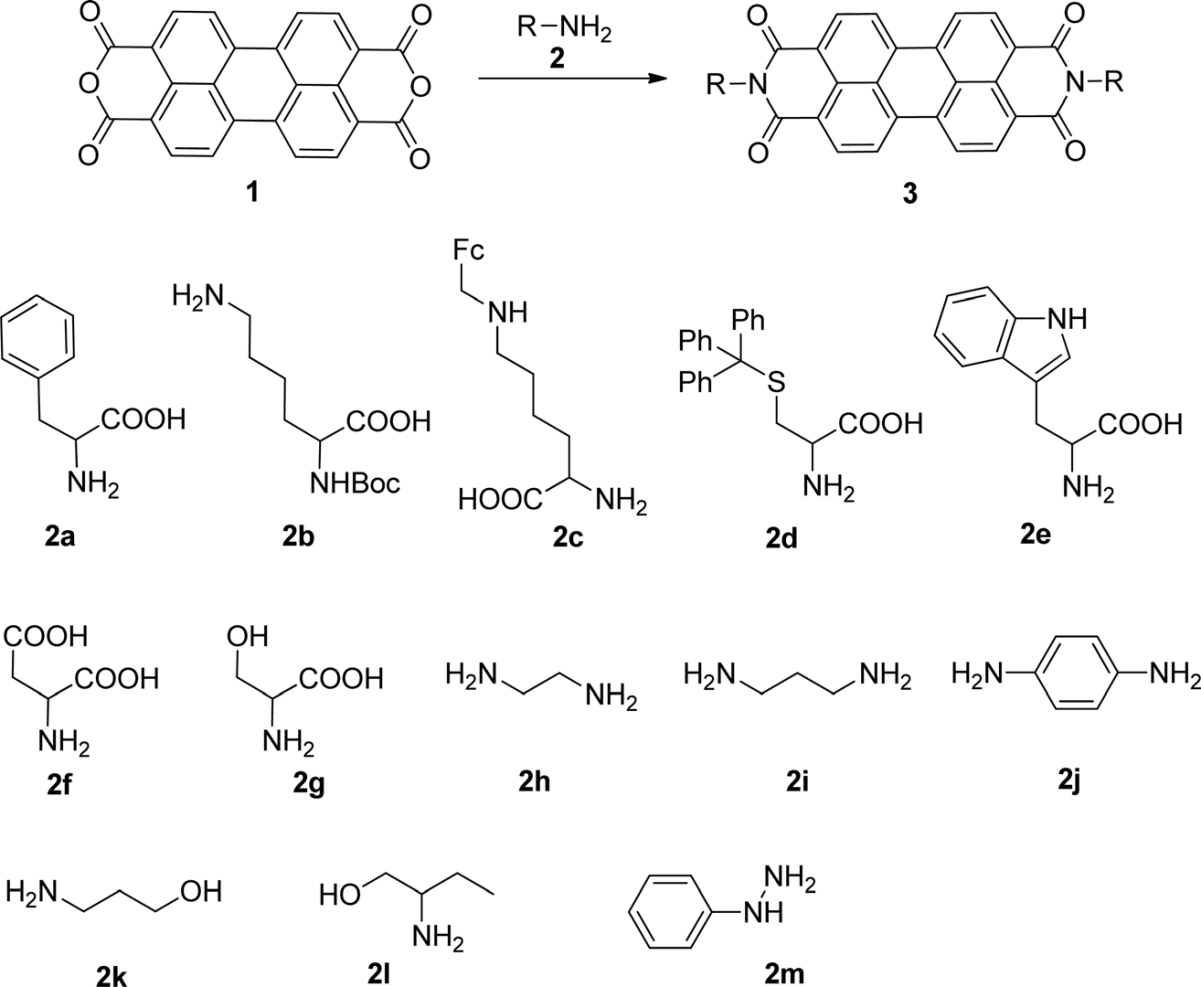			
Entry	R	Experimental conditions	Product, yield (%)
1	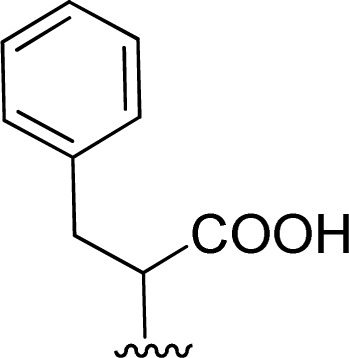	1 + 2a (2.1 eq.) + imidazole (10 eq.), 95 °C, 2 h	3a, 91%^a^
		1 + 2a (2 eq.) + imidazole (17 eq.), 110 °C, 4 h	3a, 74%^a^
2	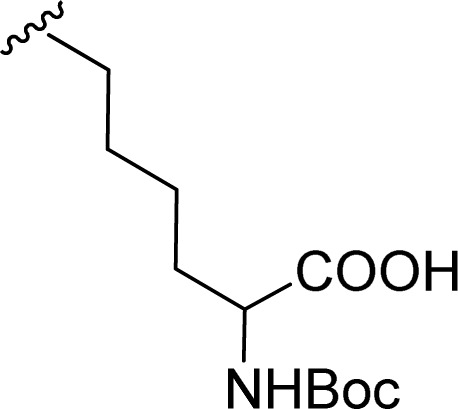	1 + 2b (2 eq.) + imidazole (17 eq.), 95 °C, 3 h	3b, 92%^a^
3	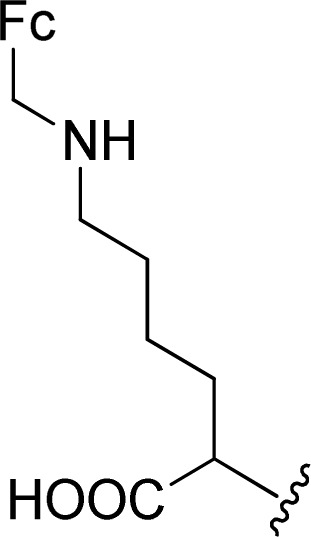	1 + 2c (2.3 eq.) + imidazole (17 eq.), 95 °C, 3 h	3c, 95%
4	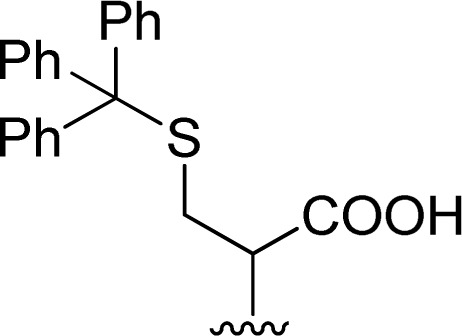	1 + 2d (2 eq.) + imidazole (17 eq.), 95 °C, 3 h	3d, 89%
5	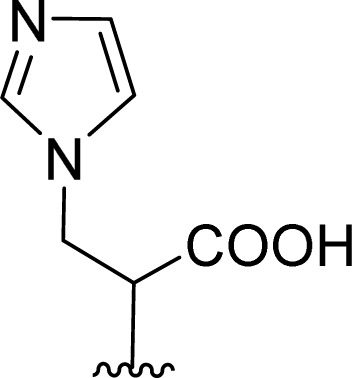	1 + 2d (2 eq.) + imidazole (17 eq.), 110 °C, 48 h	4, 39%
6	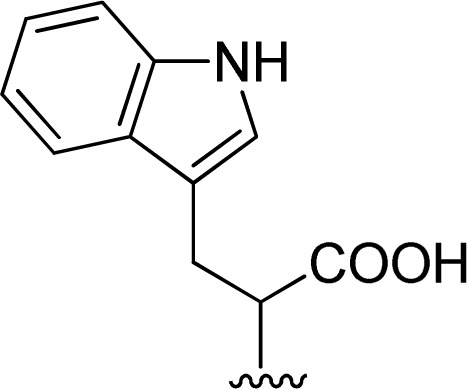	1 + 2e (2.2 eq.) + imidazole (17 eq.), 95 °C, 3 h	3e, 92%
7	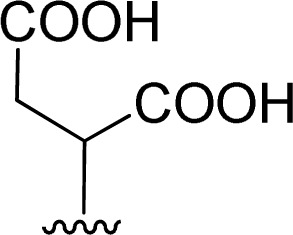	1 + 2f (2 eq.) + imidazole (17 eq.), 95 °C, 3 h	3f, 88%
8	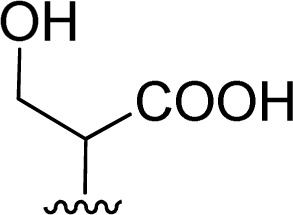	1 + 2g (2 eq.) + imidazole (17 eq.), 95 °C, 3 h	3g, 73%
9	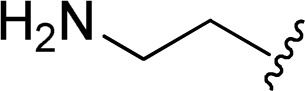	1 + 2h (10 eq.), 100 °C, 30 min	3h, 98%
10	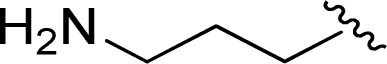	1 + 2i (10 eq.), 100 °C, 30 min	3i, 96%
11	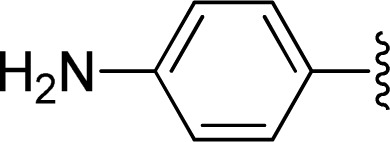	1 + 2j (2.5 eq.), EtOH, 100 °C, 2 days	3j, 74%
12	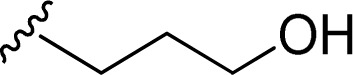	1 + 2k (10 eq.), 100 °C, 1 h	3k, 77%
13	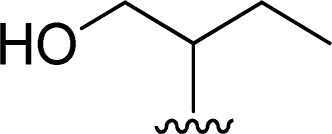	1 + 2l (10 eq.), 100 °C, 1 h	3l, 84%
14	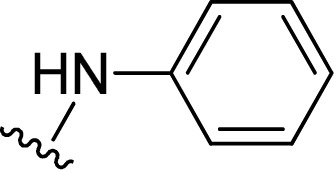	1 + 2m (101 eq.), 600 W, 1 h 40 min^b^	3m, 96%

a Reported in the literature [ref. 16].

b By microwave irradiation.

**Scheme 1:**
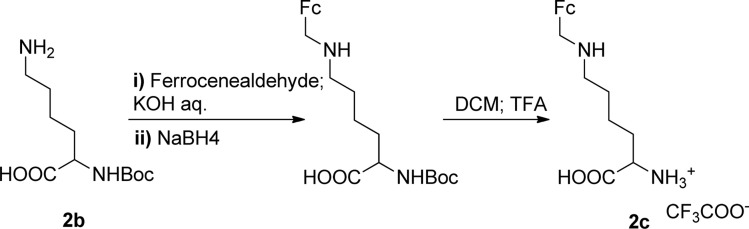
Synthesis of compound 2c.

The Boc-protected lysine (2b) was incorporated in perylene 1 and the product was isolated in 92% yield after 3 h at 95 °C (entry 2). Boc-lysine 2b was also used to incorporate a ferrocene (Fc) unit by reaction with formylated ferrocene, and the reagents were combined in a 1 : 1 molar ratio in aqueous 2.6 M KOH solution. The imine was reduced *in situ* by addition of sodium borohydride and product 2c was isolated in 53% yield after cleavage of the Boc protecting group (Scheme 1). The reaction between 2c and perylene 1 occurred smoothly in molten imidazole at 95 °C (Table 1, entry 3). The selection of ferrocene to generate PBI 3c, was inspired by the excellent redox and catalytic properties of this organometallic unit, with important applications as electrochemical sensors, biosensors and supramolecular switches.^17^ Also in medicinal chemistry, its presence in the structure of a drug can lead to additional therapeutic applications.^18^

The reaction of perylene 1 with *S*-trityl cysteine 2d in imidazole required extensive optimization studies (see Table 2). The reaction was initially tested under the standard reaction conditions and the two reagents were combined in a 1 : 2 molar ratio and heated at 95 °C for 3 h. The expected product 3d was isolated but ^1^H NMR showed the presence of traces of 5 and an unknown contaminant with aromatic protons (Table 2, entry 1). Increasing the reaction time to 4 h (entry 2) did not improve the purity of the product. The amount of amino acid was increased to 2.2 eq. (entry 3) and 2.5 eq. (entry 4) and in both cases the formation of compound 3d was accompanied by the competitive formation of perylene 4, and also of compounds 5 and 6. The signals of compounds 5 and 6 used to determine their relative amounts were the integration of the OH proton (*δ* 6.44 ppm) and CH proton (*δ* 5.60 ppm) respectively. For compounds 3d and 4, the integration of the signal at *δ* 8.37 ppm for H4′ (for 4 h) and of the signal at *δ* 6.02 ppm for H1 (for 2 h) were used, respectively. The amount of imidazole used as solvent was halved (8 molar equivalents) maintaining the 1 : 2 ratio of reagents 1 and 2d and the reaction temperature and time (95 °C, 3 h – entry 5). The pure product 3d was isolated in 61% yield.

**Table 2:** Optimization studies on the reaction of perylene 1 with cysteine-*S*-trityl 2d using imidazole as solvent

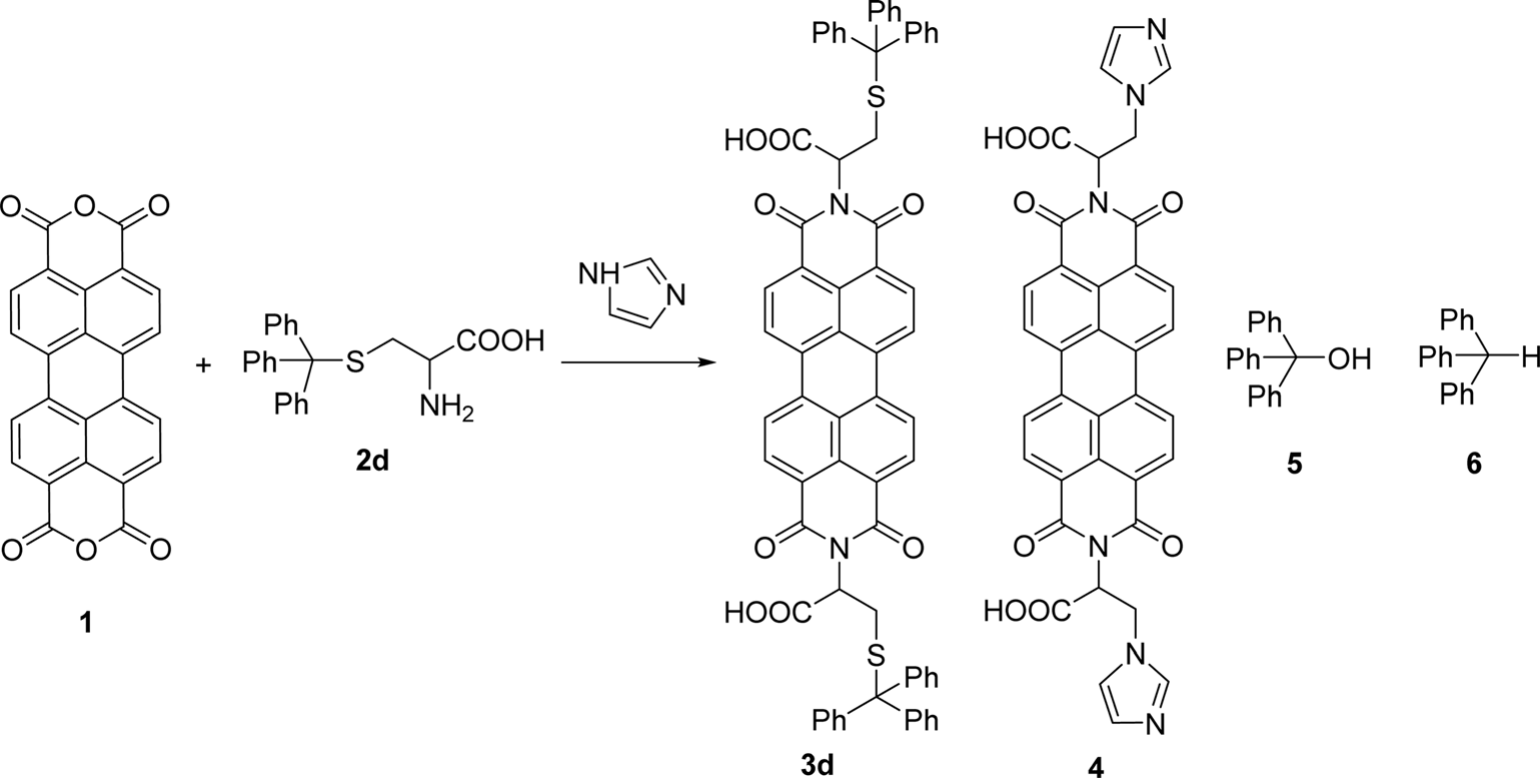					
Entry	Reaction conditions				Product
	1	2d	Imidazole	Temperature, time	
1	1 eq. (0.33 mmol)	2 eq.	17 eq.	95 °C, 3 h	3d^a^^b^
2	1 eq. (0.33 mmol)	2 eq.	17 eq.	95 °C, 4 h	3d^a^^b^
3	1 eq. (0.33 mmol)	2.2 eq.	17 eq.	95 °C, 3 h	3d + 4 + 5 + 6^a^ (7.6 : 2.3 : 1.2 : 1.0)
4	1 eq. (0.33 mmol)	2.5 eq.	17 eq.	95 °C, 3 h	3d + 4 + 5 + 6^a^ (2.5 : 1.8 : 1.0 : 1.2)
					3d, 0.24 g, 69%
5	1 eq. (0.33 mmol)	2 eq.	8 eq.	95 °C, 3 h	3d, 0.23 g, 61%
6	1 eq. (0.27 mmol)	2 eq.	17 eq.	110 °C, 3 h	3d + 4 + 5 + 6^a^ (2.1 : 1.0 : 2.1 : 2.4)
7	1 eq. (0.77 mmol)	2 eq.	17 eq.	110 °C, 4 h	3d + 4 + 5 + 6^a^ (2.7 : 1.0 : 2.1 : 2.5)
8	1 eq. (0.32 mmol)	2 eq.	17 eq.	110 °C, 27 h	4^a^^c^
9	1 eq. (0.31 mmol)	2 eq.	17 eq.	110 °C, 48 h	4, 0.08 g, 39%

a By ^1^H NMR.

b With traces of 5 and an unknown contaminant with aromatic protons.

c Not quantified; product with traces of 6 and broad signals in the aromatic region.

Attempts to understand the effect of temperature on the product mixture led us to perform the same reaction at 110 °C for 3 h (entry 6) and 4 h (entry 7), leading to a mixture of products 3d, 4, 5 and 6 in a variable ratio (Table 2). The reaction time was increased to 27 h (entry 8) and then to 48 h (entry 9) leading to the isolation of the pure product 4 in 39% yield. Cleavage of the trityl group in PBI 3d led to compound 9 in 93% isolated yield (Scheme 4).

**Scheme 2:**
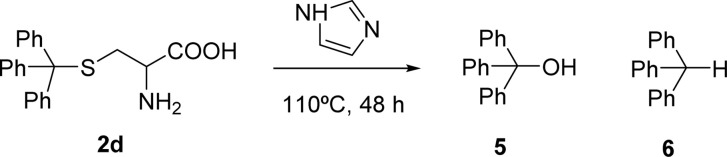
Products identified in the reaction of cysteine-*S*-trityl with imidazole.

The formation of PBI 4 was unexpected and the replacement of the *S*-trityl group by the imidazole nucleus was also tested using only *S*-trityl cysteine and 17 molar equivalents of imidazole with heating at 110 °C for 48 h (Scheme 2). The product was isolated as a beige solid after washing with water, and identified by NMR spectroscopy as compound 6 (*δ*_H_ 5.60 ppm for the C–H proton and *δ*_C_ 55.79 ppm) with traces of compound 5 (*δ*_H_ 6.44 ppm for the OH signal and *δ*_C_ 80.58 ppm) formed in a 9 : 1 molar ratio. Although cysteine is no longer identified in the reaction mixture, the replacement of the *S*-trityl group by the imidazole was not observed. The absence of sulfur in the isolated solid mixture was confirmed by the lead acetate paper test.

Attempts to understand the evolution of *S*-trityl cysteine under these experimental conditions, prompted us to perform the reaction in an NMR tube and accompany the progression with time.

In the first experiment, *S*-trityl cysteine (6.6 mg) was combined with imidazole (8 molar equivalents) in DMSO-d_6_ (600 μL). The NMR tube was heated at 110 °C and the reaction was followed every hour for 28 h. After 1 h, *S*-trityl cysteine was completely absent and only imidazole and triphenylmethane 6 were visible in the spectrum. The experiment was repeated under the same experimental conditions, but the reaction was followed by ^1^H NMR every 5 minutes, for a total of 1 hour. After 15 minutes, the *S*-protected cysteine was absent and traces of alanine were visible in the spectrum (*δ*_H_ 1.23 ppm d, *J* = 7.2 Hz for the CH_3_ protons) together with a broad singlet at *δ*_H_ 6.1 ppm that was later confirmed to exchange with D_2_O. The total absence of correlation with other carbon atoms in the ^13^C NMR spectrum prevented the identification of this new molecule. After 20 minutes, triphenylmethane 6 was also visible, and its formation gradually increased with time, as the signals for alanine and the unknown molecule (*δ*_H_ 6.1 ppm) were fading away and completely disappeared after 1 h.

The experiment was repeated in the absence of imidazole and *S*-trityl cysteine was heated in DMSO-d_6_ at 110 °C. After 10 minutes, alanine and the unknown compound (*δ*_H_ 6.1 ppm) were visible, and after 15 minutes the proton signal for triphenylmethane 6 was also present. After 20 minutes, all these signals increased and a broad signal at *δ*_H_ 6.4 ppm, assigned to the OH proton of triphenylmethanol 5 also started to appear. With continued heating, the signals for alanine and the unknown compound gradually faded away leading to a 1.5 : 2 mixture of compounds 5 and 6 as the major visible products after 1 h.

The evolution of *S*-trityl cysteine in DMSO-d_6_ was also followed at room temperature (20–22 °C) both in the absence and in the presence of imidazole (8 molar equivalents).

In the absence of imidazole, traces of alanine were visible in the spectrum after 4 days and traces of triphenylmethane after 9 days. After one month, only traces of *S*-trityl cysteine were visible in the spectrum, where the major products were triphenylmethanol 5 alanine and triphenylmethane 6 in a 4 : 1.5 : 1 molar ratio.

When imidazole was present, this evolution was considerably delayed, as alanine could only be detected after 9 days and triphenylmethane 6 after 14 days. After one month, *S*-trityl cysteine and alanine were still present in a 3 : 1 molar ratio, together with traces of triphenylmethane.

The serendipitous observation, in our experimental work, that the *S*-trityl group in *S*-trityl cysteine was cleaved in DMSO-d_6_ leading to alanine and that the rate of this process, that occurs at room temperature, was considerably accelerated by heating at 110 °C, can inspire future work on a new gentle process for the desulfurization of cysteine. As far as we know, this reaction was never reported and mild desulfurization strategies are important when peptide and protein synthesis are performed through a native chemical ligation-desulfurization approach.^19^

**Scheme 3:**
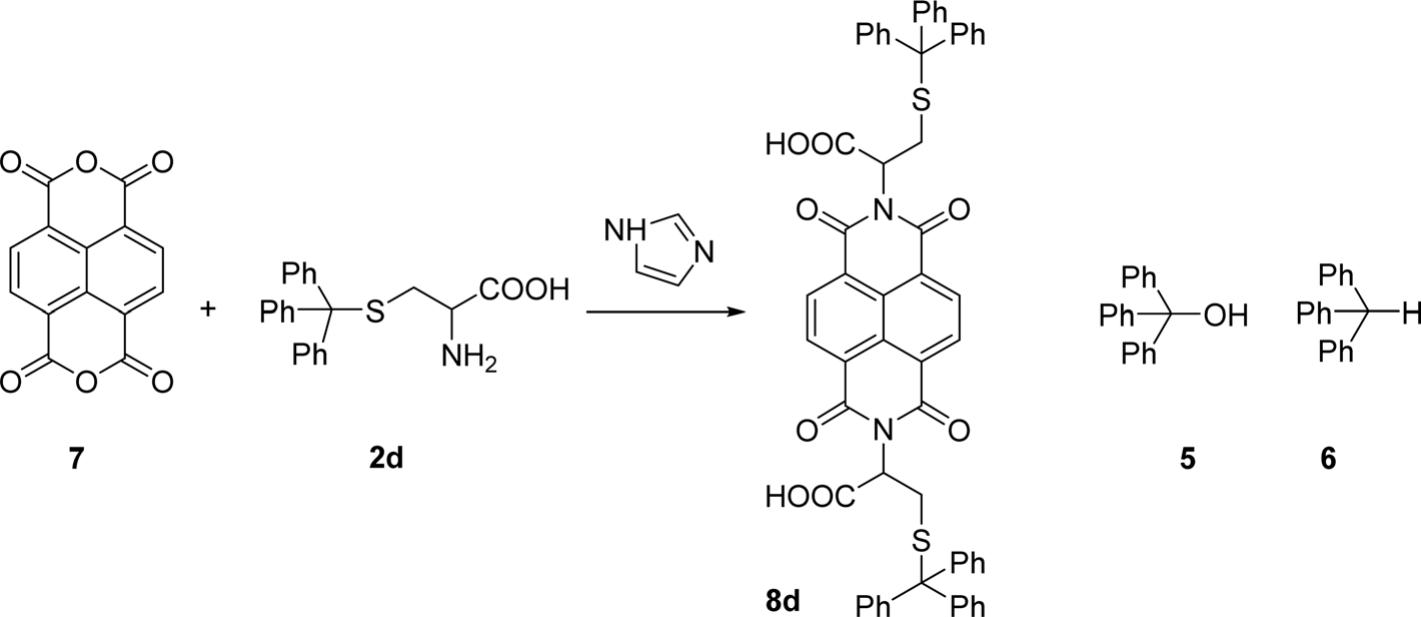
Condensation of naphthalene tetracarboxylic dianhydride with cysteine-*S*-trityl.

Naphthalenebisimides (NBI) are structural analogues of PBIs and equally important as n-type semiconductor materials,^20^ for organic electronics and in supramolecular chemistry.^21^ The reaction of naphthalenetetracarboxylic dianhydride 7 with 2 eq. of the *S*-trityl cysteine 2d in melted imidazole (17 eq.) was performed at 95 °C for 3 h, reproducing the conditions used for the perylene analogue. The NBI 8d was isolated in 53% yield and products 5 and 6 were also identified in the reaction mixture (Scheme 3).

The reaction of perylene dianhydride 1 with amino acids 2e, 2f and 2g under the normal reaction conditions, summarized in Table 1 (entries 6, 7 and 8 respectively) led to the isolation of the corresponding PBIs 3e, 3f and 3g in 92, 88 and 73% yield after 3 h at 95 °C using imidazole as solvent. Mahapatra *et al.*,^11*c*^ reported the synthesis of PBI 3f, when the dianhydride 1 was combined with aspartic acid in imidazole and the mixture was heated at 120 °C for 8 h, under a nitrogen atmosphere. The product was isolated in 82% yield and used in the detection and quantification of caffeine in aqueous medium.

The reaction of perylene dianhydrides 1 with aliphatic and aromatic diamines or aminoalcohols was also carried out, using an excess of amine as solvent (2.5–10 eq.) (Table 1, entries 9–13). The reactions were performed at 100 °C for 0.5–1 h for alkyl amines or for 2 days with the less nucleophilic arylamine (entry 11). The synthesis of PBI 3h, was already reported in the literature and the reagents were refluxed in benzene for 5 h (84%)^10*a*^ or perylene 1 and a large excess of ethylenediamine were heated for 24 h at 90 °C, followed by 2 h at 150 °C (86%).^22^

In this work, perylene 1 was also reacted with phenylhydrazine and the reaction required the use of a large excess of amine under microwave irradiation (600 W) for over one hour (entry 14), leading to the expected product 3m in 96% yield.

**Scheme 4:**
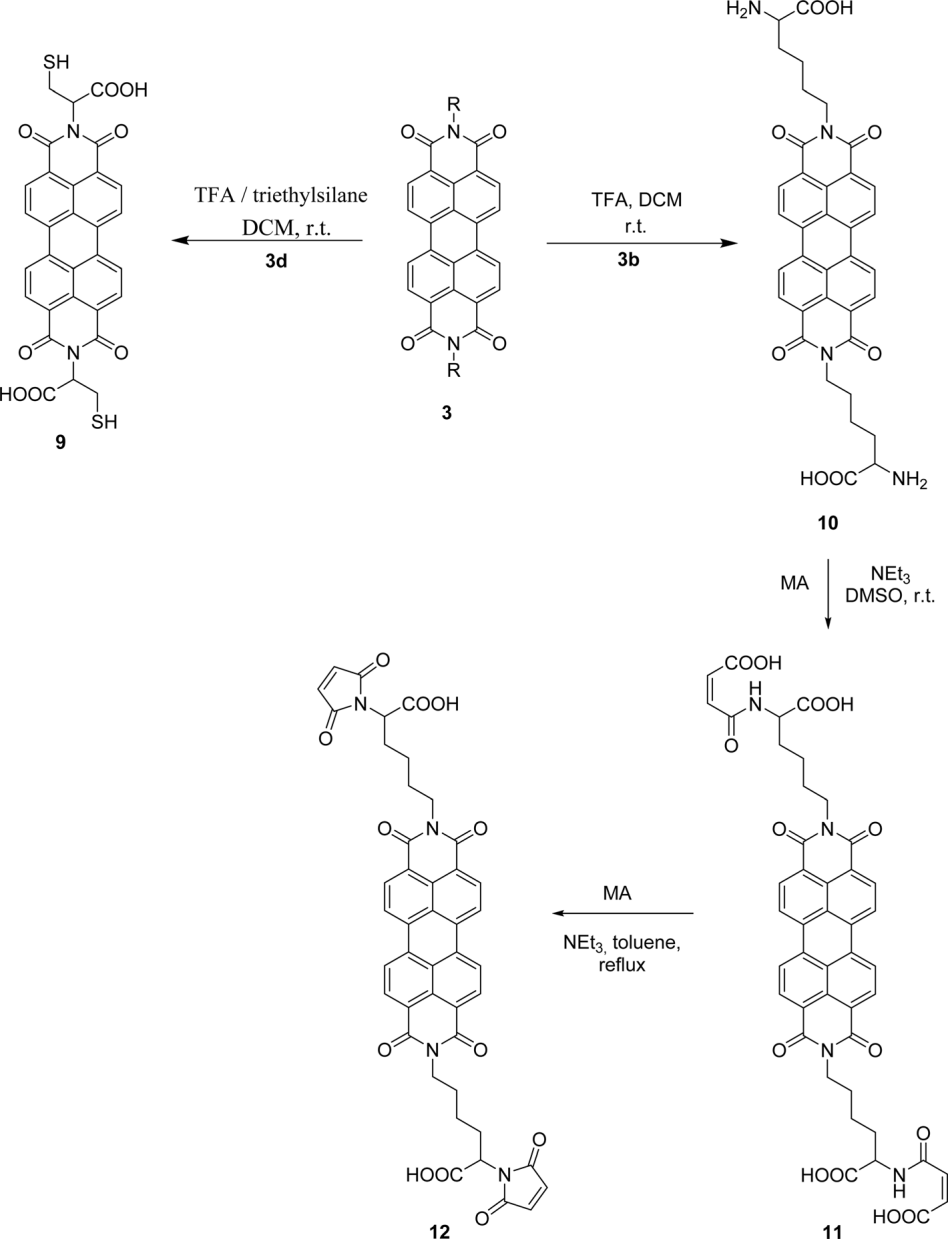
Cleavage of the protecting group in compounds 3d and 3b and synthesis of PBI 12.

PBIs 3a and 3b were functionalized on the exocyclic amino acid chain by incorporating either maleimide or anthraquinone. The maleimide unit is an important asset that provides access to valuable derivatives by nucleophilic addition to the double bond, although its presence in macromolecular materials is still limited. In order to incorporate this group into PBI 3b, the Boc protecting group in this compound was removed (Scheme 4). The use of TFA in DCM, at room temperature, led to PBI 10, isolated in 92% yield. The work proceeded with the reaction of compound 10 with maleic anhydride (MA) activated by trimethylamine and using DMSO as solvent, at room temperature. After 5 h, product 11 was isolated in 96% yield. The desired product 12 could not be obtained by increasing the reaction temperature or the reaction time, as extensive decomposition of the reaction mixture occurred, leading to polymeric material. The use of an extra amount of maleic anhydride and its fractional addition during the reaction period allowed the isolation of compound 12 in 73% yield.

**Scheme 5:**
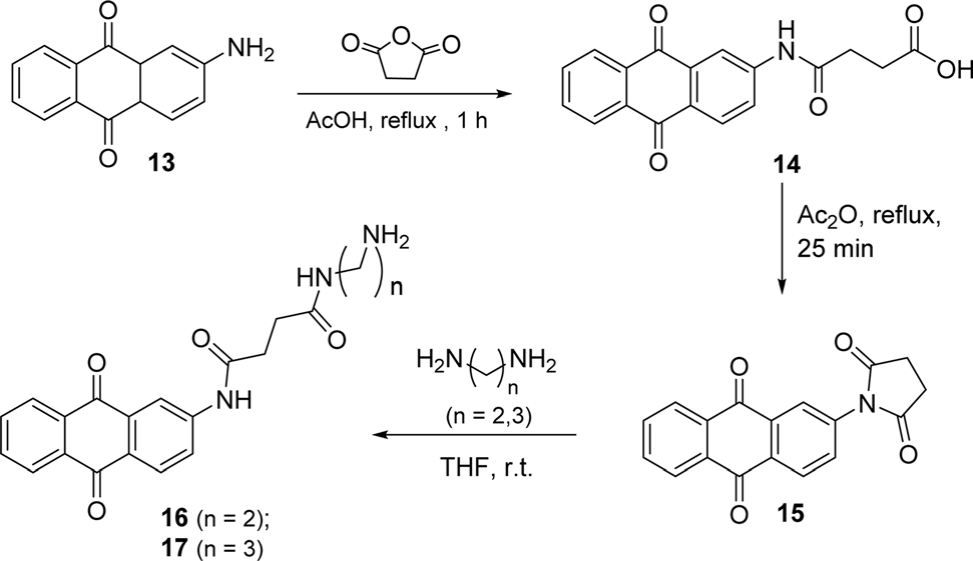
Synthesis of compounds 16 and 17.

The anthraquinone motif, mainly used as a redox catalyst, has wide industrial applications. The aim of this part of the work was to anchor this unit to the perylene aromatic surface through different linkers. The 2-aminoanthraquinone was the starting material to build a side arm, used as a linker, and reaction with succinic anhydride in glacial acetic acid and reflux for 1 h (Scheme 5) led to the formation of compound 14. Compound 15 was prepared from 14 and intramolecular cyclization involving the nitrogen atom of the amide and the carbonyl group of the carboxylic acid occurred in the presence of acetic anhydride, used as a solvent. The synthesis of compounds 16 and 17 resulted from the reaction of compound 15 with ethylenediamine or propane-1,3-diamine using THF as solvent, under stirring at room temperature.

**Scheme 6:**
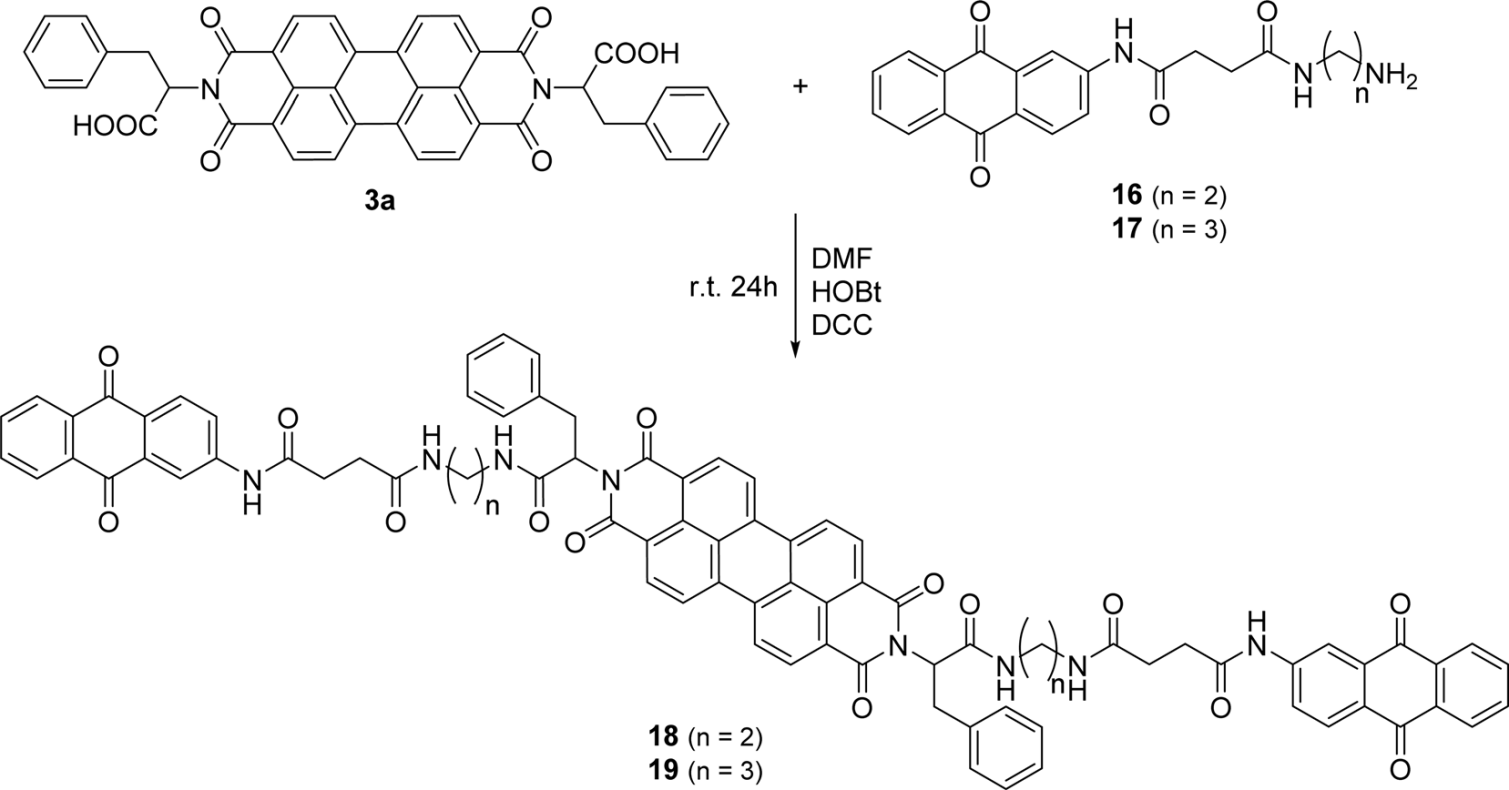
Synthesis of compounds 18 and 19.

PBI 3a was selected to incorporate anthraquinone derivatives 16 and 17 due to the presence of the phenyl group in the side chain, reducing aggregation and improving solubility. The carboxylic acid allowed the formation of an amide bond, using standard peptide coupling conditions. The carboxylic function in compound 3a was pre-activated with HOBt and DCC in DMF (Scheme 6) and reaction with the amino group of compound 16 or 17 led to the final products 18 and 19 isolated in 36% and 50% yield, respectively.

Attempts to characterize all the compounds by NMR spectroscopy were hampered by their poor solubility in DMDO-d_6_ and a few drops of TFA were added to the NMR tube. The formation of the corresponding salt improved the solubility in this solvent, allowing NMR analysis. The product of the reaction of perylenetetracarboxylic dianhydride 1 with the aminoalcohol 2k could not be characterized by NMR due its poor solubility even in the presence of TFA. This may be related to the linear shape of the aminoalcohol that favors aggregation of the corresponding PBI 3k. The use of 2-amino-1-butanol 2l in the reaction with perylenetetracarboxylic dianhydride 1 resulted in the formation of a branched PBI 3l, which reduced aggregation and allowed a good solubility in DMSO-d_6_.

## 3. Conclusion

In conclusion, we developed an efficient method to prepare perylene bisimides from the reaction of perylene tetracarboxylic dianhydride with different amino acids, amino-alcohols and primary aromatic amines. The products were isolated in 73–98% yield from a reaction that proceeded smoothly at 95–110 °C, using mostly equimolar amounts of the reagents in molten imidazole. The side chain functionality was explored to incorporate ferrocene, maleimide or anthraquinone, all appealing groups not only for research but also for a wide range of industrial applications.

The unexpected evolution of *S*-trityl cysteine in the perylene side chain to 1*H*-imidazolil propanoic acid was observed only when the amino acid was associated to the perylene ring. When a solution of *S*-trityl cysteine in DMSO, was kept either at room temperature or under heating at 110 °C, the equally unexpected formation of alanine was detected by ^1^H NMR. New desulfurization strategies, transforming a cysteine into an alanine unit, are important approaches for peptide and protein synthesis. A concept article recently published by Li *et al.*,^19*a*^ reports metal-based and metal-free approaches and P–B desulfurization methods, but none of them involved the use of *S*-trityl cysteine, as is reported in the present work.

## Author contributions

E. M., P. F. and R. A. performed chemical synthesis and characterization data of all compounds. E. M. wrote the original draft of the manuscript. E. M., P. F., R. A. and M. F. P. reviewed and edited the manuscript. M. F. P. supervised the project. All authors have read and agreed to the published version of the manuscript.

## Conflicts of interest

There are no conflicts of interest to declare.

## Supplementary Material

RA-014-D4RA01576B-s001
