# Supplementary material for: A simple protocol for the synthesis of perylene bisimides from perylene tetracarboxylic dianhydride
Source: RSC Adv. 2024 Apr 8;14(16):11141–50. doi: 10.1039/d4ra01576b (PMC10999908; doi:10.1039/d4ra01576b)
Supplement: RA-014-D4RA01576B-s001 [file RA-014-D4RA01576B-s001.pdf]

Electronic Supplementary Material (ESI) for RSC Advances.
This journal is © The Royal Society of Chemistry 2024

A Simple Protocol for the Synthesis of Perylene Bisimides from Perylene Tetracarboxylic Dianhydride

Elina Marinho,[a] Pedro R. Figueiredo,[a] Rui Araújo,[a] and M. Fernanda Proença*[a]

[a] Department of Chemistry, University of Minho, Campus de Gualtar, 4710-057 Braga, Portugal;
Tel: +351 253604379; E-mail: fproenca@quimica.uminho.pt

Supporting information

Table of Contents

I. General information	S2
II. Reaction of Ferrocenealdehyde with Boc-Lysine	S2
III. Synthesis and Characterization of (2-{[(5-amino-5-carboxypentyl)amino]methyl}cyclopenta-2,4-dien-1-id-1-yl)(cyclopenta-2,4-dien-1-id-1-yl)ironbis(ylium) **2c**	S3
IV. General procedure for compounds **3a-m**	S3
V. Characterization of products **3a-m**	S4
VI. Synthesis and Characterization of compound **4**	S8
VII. Synthesis and Characterization of compounds **9-12**	S9
VIII. Synthesis and Characterization of compounds **14-17**	S11
IX. Synthesis and Characterization of compounds **18-19**	S13
X. NMR spectra of products	S16
XI. Evolution of S-trityl cysteine	S47
XII. References	S51

I. General information

All compounds were fully characterized by elemental analysis and spectroscopic data. The NMR spectra were recorded on a Varian Unity Plus (^{1}H: 300 MHz, ^{13}C: 75 MHz), or on a Bruker Avance III 400 (1H: 400 MHz, 13C: 100 MHz, ^{15}N: 40 MHz) including the ^{1}H-^{13}C and ^{1}H-^{15}N correlation spectra (HMQC and HMBC). Deuterated DMSO-d_6 was used as solvent. The peak patterns are indicated as follows: s, singlet; d, doublet; t, triplet; dd, doublet of doublets; m, multiplet; q, quartet and br, broad. The coupling constants J, are reported in hertz (Hz). The reactions under microwave irradiation were performed on a CEM microwave reactor, model Mars 5, using a quartz open vessel. Infra-red spectra were recorded on a FTIR Bomem MB 104 using Nujol mulls with NaCl cells or using KBr pellets (about 1 mg of compound in 99 mg of KBr). All reactions were monitored by thin layer chromatography (TLC) using silica gel 60 F254 (Merck). The melting points were determined on a Stuart SMP3 melting point apparatus and are uncorrected. Elemental analyses were performed on a LECO CHNS-932 instrument.

II. Reaction of Ferrocenealdehyde with Boc-Lysine

Ferrocenealdehyde (0.56 g, 2.6 mmol) was added a solution of Boc-Lysine **2b** (0.64 g; 2.6 mmol) in 1 mL of aqueous solution of KOH (2.6 M). The mixture was stirred at room temperature for 1 h. The mixture was cooled in an ice bath and $NaBH_4$ was added (0.13 g, 3.4 mmol). The mixture was stirred at room temperature for 1 h. A few drops of HCl conc. were added leading to the formation of a yellow solid. The solid was filtered, washed with distillated water and dried at 40 ºC in a vacuum pistol for 16 h and identified as (2-{[(5-{[(tert-butoxy)carbonyl]amino}-5-carboxypentyl)amino]methyl}cyclopenta-2,4-dien-1-id-1-yl)(cyclopenta-2,4-dien-1-id-1-yl)ironbis(ylium) (0.79 g; 68%).

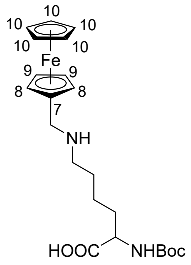

^{1}H NMR (400 MHz, DMSO-d_6 + TFA, ppm) δ 8.43 (brs, 1H, NH), 7.2 (d, 1H, NH, J = 7.6 Hz), 4.37 (s, 2H, H-8), 4.24 (s, 2H, H-9), 4.2 (s, 5H, H-10), 3.9-3.8 (m, 2H, H-6), 3.8-3.65 (m, 1H, H-1), 2.9-2.7 (m, 2H, H-5), 1.65-1.5 (m, 4H), 1.36 (s, 9H, Me), 1.36-1.2 (m, 2H, H-2, H-3 or H-4); ^{13}C NMR (100 MHz, DMSO-d_6 + TFA, ppm) δ 174.2 (COOH), 155.7 (CO), 78.13 (C-7), 76.8 (C-(Me)$_3$), 70.37 (C-8), 68.95 (C-9), 68.80 (C-10), 53.25 (C-1), 46.3 (C-6), 45.8 (C-5), 30.18 (C-2, C-3 or C-4), 28.24 (Me), 24.90 (C-2, C-3 or C-4), 22.73 (C-2, C-3 or C-4).

III. Synthesis and Characterization of (2-{[(5-amino-5-carboxypentyl)amino] methyl}cyclopenta-2,4-dien-1-id-1-yl)(cyclopenta-2,4-dien-1-id-1-yl)ironbis (ylium) 2c

Cleavage of the BOC group in compound (2-{[(5-{[(tert-butoxy)carbonyl]amino}-5-carboxypentyl)amino]methyl}cyclopenta-2,4-dien-1-id-1-yl)(cyclopenta-2,4-dien-1-id-1-yl)ironbis(ylium) (0.78 g; 1.80 mmol) was carried out in 5 mL of dichloromethane with 500 µL of TFA. The mixture was stirred at room temperature for 15 h. The solvent and the excess of TFA were eliminated on the rotary evaporator. The solid was recovered by filtration using diethyl ether as washing solvent and identified as (2-{[(5-amino-5-carboxypentyl)amino]methyl}cyclopenta-2,4-dien-1-id-1-yl)(cyclopenta-2,4-dien-1-id-1-yl)ironbis(ylium) **2c** (0.44 g; 0.96 mmol; 53%).

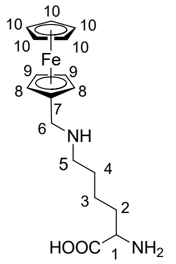

^{1}H NMR (400 MHz, DMSO-d_6 + TFA, ppm) δ 8.40 (brs, 3H, NH_2 + NH); 4.37 (s, 2H, H-8), 4.25 (s, 2H, H-9), 4.2 (s, 5H, H-10), 3.9 (s, 2H, H-6), 3.75-3.65 (m, 1H, H-1), 2.9-2.7 (m, 2H, H-5), 1.85-1.65 (m, 2H, H-2, H-3 or H-4), 1.60-1.46 (m, 2H, H-2, H-3 or H-4), 1.45-1.3 (m, 2H, H-2, H-3 or H-4); ^{13}C NMR (100 MHz, DMSO-d_6 + TFA, ppm) δ 170.84 (COOH), 76.84 (C-7), 70.29 (C-8), 68.88 (C-9), 68.73 (C-10), 52.01 (C-1), 46.14 (C-6), 45.52 (C-5), 29.53 (C-2, C-3 or C-4), 26.60 (C-2, C-3 or C-4), 24.53 (C-2, C-3 or C-4); FT-IR (KBr, cm^{-1}) 763, 825, 956, 1010, 1047, 1102, 1245, 1276, 1327, 1455, 1637, 2600-3330.

IV. General procedure for the synthesis of compounds 3a-m

- For compounds **3a-3g**

Perylenetetracarboxylic dianhydride (1 equiv.) was combined with approximately 2 equiv. of the corresponding amino acid and 17 equiv. of imidazole. The reaction mixture was kept at 95 ºC (melting point of imidazole) under magnetic stirring for 3 h. The mixture was dissolved in distilled water and the unreacted perylenetetracarboxylic dianhydride was removed by filtration. The synthesized perylene bisimide was precipitated from solution by the dropwise addition of conc. HNO_3. The solid was filtered and washed with distilled water in order to eliminate the excess of imidazole. The product was dried for 24 h at 100 ºC in a vacuum pistol.

- For compounds **3h-3m**

Perylenetetracarboxylic dianhydride (1 equiv.) was combined with 2.5 equiv. (**2j**), 10 equiv. (**2h**, **2i**, **2k**, **2l**) or 101 equiv. (**2m**) of the corresponding amine. The reaction mixture was kept at 100 ºC under magnetic stirring for 30 min – 2 days or 600 W for 1 h and 40 min.. The solid was filtered and washed with water, ethanol and diethyl ether. The product was dried for 24 h at 100 ºC in a vacuum pistol.

V. Characterization of compounds 3a-m

Synthesis of 2,2'-(1,3,8,10-tetraoxo-1,3,8,10-tetrahydroanthra[2,1,9-def:6,5,10-d'e'f']diisoquinoline-2,9-diyl)bis(3-phenylpropanoic acid) (3a)

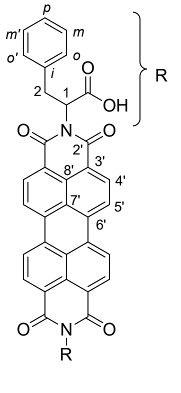

The synthesis of compound **3a** was previously reported in the literature.[1] The product was isolated with 91% yield. ^{1}H NMR (400 MHz, DMSO-d$_6$, 80 °C, ppm) δ 8.38 (d, 4H, H-5', J = 7.6 Hz), 8.30 (d, 4H, H-4', J = 7.6 Hz), 7.23 (d, 4H, H*o* + H*o*', J = 7.6 Hz), 7.16 (t, 4H, H*m* + H*m*', J = 7.2 Hz), 7.06 (t, 2H, H*p*, J = 7.2 Hz), 5.93 (dd, 2H, H-1, J = 9.2 Hz and J = 5.6 Hz), 3.65 (dd, 2H, H-2, J = 14.4 Hz and J = 5.6 Hz), 3.45 (dd, 2H, H-2, J = 14.4 Hz and J = 9.2 Hz); ^{13}C NMR (100 MHz, DMSO-d$_6$, 80 °C, ppm) δ 169.95 (COOH), 161.95 (C-2'), 137.66 (C*i*), 133.62 (C-6'), 130.75 (C-4'), 128.63 (C*o* + C*o*'), 127.94 (C-8'), 127.75 (C*m* + C*m*'), 125.95 (C*p*), 125.07 (C7'), 123.36 (C-5'), 121.67 (C-3'), 53.87 (C-1), 34.21 (C-2); ^{15}N NMR (40.6 MHz, DMSO-d$_6$, 80 °C, ppm) δ 176.1 (N-1').

Synthesis of 6,6'-(1,3,8,10-tetraoxoanthra[2,1,9-def:6,5,10-d'e'f']diisoquinoline-2,9(1*H*,3*H*,8*H*,10*H*)-diyl)bis(2-((tert-butoxycarbonyl)amino)hexanoic acid) (3b)

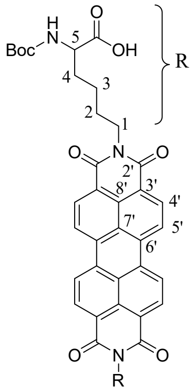

The synthesis of compound **3b** was previously reported in the literature.[1] The product was isolated with 92% yield. ^{1}HNMR (400 MHz, DMSO-d$_6$, ppm) δ 7.56 (brs, 8H, H-4' and H-5'), 7.00 (d, 2H, N-H, J = 7.6 Hz), 3.91-3.78 (m, 6H, H-1 and H-5), 1.74-1.61 (m, 4H, H-4), 1.56-1.46 (m, 8H, H-2 and H-3), 1.36 (s, 18H, Me group from Boc); ^{13}C NMR (100 MHz, DMSO-d$_6$, ppm) δ 174.37 (COOH), 161.80 (C-2'), 155.67 (C=O from Boc), 132.20 (C-6'), 129.44 (C-4'), 126.79 (C-8'), 123.57 (C-7'), 122.93 (C-5'), 121.09 (C-3'), 78.00 (C(Me)$_3$ from Boc), 53.58 (C-5), 39.71 (C-1), 30.70 (C-4), 28.19 (Me group from Boc), 27.04 (C-2), 23.33 (C-3).

Synthesis of [2-({[5-carboxy-5-(18-{1-carboxy-5-[({2-[(cyclopenta-2,4-dien-1-id-1-yl)ferriobis(ylium)] cyclopenta-1(5),3-dien-2-id-1-yl}methyl)amino]pentyl}-6,8,17,19-tetraoxo-7,18-diazaheptacyclo [14.6.2.22,5.03,12.04,9.013,23.020,24]hexacosan-7-yl)pentyl]amino}methyl)cyclopenta-2,4-dien-1-id-1-yl](cyclopenta-2,4-dien-1-id-1-yl)ironbis(ylium (3c)

95%, m.p. > 300 ºC; ^{1}H NMR (400 MHz, DMSO-d$_6$ + TFA, ppm) δ 8.80-7.80 (m, 8H, H-4' and H-5'), 5.6-5.3 (m, 2H, H-1), 4.37 (brs, 4H, H-8), 4.20 (s, 4H, H-9), 4.17 (s, 10H, H-10), 4.00-3.70 (m, 4H, H-6), 2.9-2.7 (m, 4H, H-5), 2.40-2.0 (m, 4H, H-2), 1.80-1.50 (m, 4H, H-3 or H-4), 1.5-1.2 (m, 4H, H-3 or H-4);

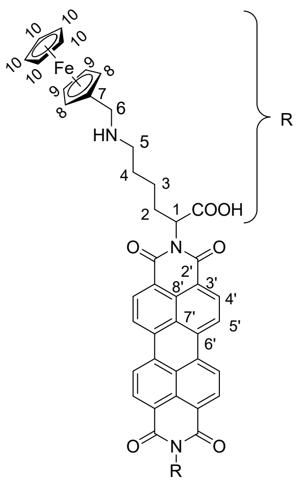

^{13}C NMR (100 MHz, DMSO-d$_6$, ppm) δ 171.30 (COOH), 162.51 (C=O), 133.58 (C-6'), 130.99 (C-4'), 128.13 (C-8'), 124.88 (C-7'), 123.62 (C-5'), 121.90 (C-3'), 77.02 (C-7), 70.46 and 70.41 (C-8), 68.96 (C-9), 68.83 (C-10), 53.47 (C-1), 46.34 (C-6), 46.03 (C-5), 28.52 (C-2), 25.54 (C-3 or C-4), 23.47 (C-3 or C-4); FT-IR (KBr, cm^{-1}) 3088 (broad), 1696, 1655, 1592, 1443, 1401, 1363, 1344, 1252, 1104, 855, 808, 746, 648. Anal. Calcd for $C_{62}H_{66}Fe_2N_4O_8$: C, 67.28; H, 6.01; N, 5.06; Fe, 10.09. Found: C, 67.20; H, 6.09; N, 4.98; Fe, 9.93.

Synthesis of 2,2'-(1,3,8,10-tetraoxoanthra[2,1,9-def:6,5,10-d'e'f']diisoquinoline-2,9(1*H*,3*H*,8*H*,10*H*)-diyl)bis(3-(tritylthio)propanoic acid) (3d)

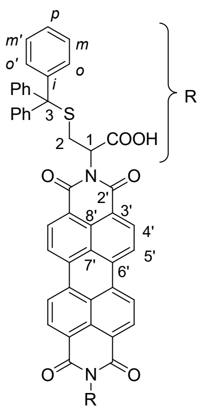

89%, m.p. > 300 ºC; ^{1}H NMR (400 MHz, DMSO-d$_6$, ppm) δ 8.51 (d, 4H, H-5', J = 7.6 Hz), 8.37 (d, 4H, H-4', J = 7.6 Hz), 7.4-7.1 (m, 30H, Ph), 5.61-5.57 (m, 2H, H-1), 3.10 (d, 4H, H-2, J = 7.2 Hz); ^{13}C NMR (100 MHz, DMSO-d$_6$, ppm) δ 169.49 (COOH), 162.06 (CO), 147.73 (C*i*), 134.10 (C-6'), 131.45 (C-4'), 129.10 (C*o* + C*o'*), 128.65 (C-8'), 126.90 (C*p*), 128.10 (C*m* + C*m'*), 125.71 (C-7'), 124.30 (C-5'), 121.75 (C-3'), 66.48 (C-3), 52.03 (C-1), 30.49 (C-2); ^{15}N NMR (40.6 MHz, DMSO-d$_6$, ppm) δ 205.32 (N-1'). FT-IR (KBr, cm^{-1}) 3050, 2935, 1739, 1702, 1665, 1594, 1488, 1436, 1402, 1362, 1344, 1257, 1181, 1124, 1083, 1030, 978, 855, 809, 745, 700; Anal. Calcd for $C_{68}H_{46}S_2N_2O_8$: C, 75,40; H, 4.28; N, 2.59; S, 5.92. Found: C, 75,48; H, 4.36; N, 2.44; S, 5.86.

Synthesis of 2,2'-(1,3,8,10-tetraoxoanthra[2,1,9-def:6,5,10-d'e'f']diisoquinoline-2,9(1*H*,3*H*,8*H*,10*H*)-diyl)bis(3-(1*H*-indol-3-yl)propanoic acid) (3e)

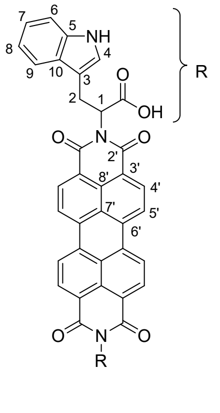

92%, m.p. > 300 ºC; ^{1}H NMR (400 MHz, DMSO-d$_6$, ppm) δ 10.67 (s, 2H, NH), 7.78-7.71 (m, 4H, H-4' or H-5'), 7.70 (dd, 2H, H-9, J = 7.6 Hz and J = 2.4 Hz), 7.30 (d, 2H, H-4, J = 7.6 Hz), 7.17 (dd, 2H, H-6, J = 8.0 Hz and J = 2.4 Hz), 7.48-7.01 (m, 4H, H-4' or H-5'), 7.00 (t, 2H, H-8, J = 7.6 Hz), 6.91 (t, 2H, H-7, J = 7.6 Hz), 6.13 (t, 2H, H-1, J = 7.6 Hz), 3.81-3.68 (m, 4H, H-2); ^{13}C NMR (100 MHz, DMSO-d$_6$, ppm) δ 171.06 (COOH), 162.08 (C-2'), 136.03 (C-5), 132.82 (C-6'), 130.66 (C-4'), 127.65 (C-10), 124.47 (C-8'), 123.94 (C-4), 123.81 (C-7'), 122.56 (C-5'), 121.53 (C-3'), 121.04 (C-7), 118.51 (C-8), 118.40 (C-9), 111.40 (C-6), 110.18 (C-3), 53.44 (C-1), 24.01 (C-2); FT-IR (KBr, cm^{-1}) 3391, 3057, 2937, 2598, 1730, 1696, 1656, 1593, 1436, 1401, 1364, 1345, 1255, 1171, 1129, 1094, 1011, 979, 951, 857, 809, 746. Anal. Calcd for $C_{46}H_{28}N_4O_8$: C, 72.25; H, 3.69; N, 7.33. Found: C, 72.22; H, 3.64; N, 7.38.

Synthesis of 2,2'-(1,3,8,10-tetraoxoanthra[2,1,9-def:6,5,10-d'e'f']diisoquinoline-2,9(1*H*,3*H*,8*H*,10*H*)-diyl)disuccinic acid (3f)

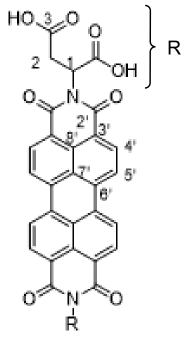

88%, m.p. > 300 ºC; ^{1}H NMR (400 MHz, DMSO-d_6, ppm) δ 12.78 (brs, 4H, COOH), 7.99 (d, 4H, H-4’, J = 7.6 Hz), 7.81 (d, 4H, H-5’, J = 7.6 Hz), 6.01 (dd, 2H, H-1, J = 8.4 Hz and J = 4.0 Hz), 3.39 (dd, 2H, H-2, J = 16.8 Hz and J = 8.4 Hz), 2.83 (dd, 2H, H-2, J = 16.8 Hz and J = 4.0 Hz); ^{13}C NMR (100 Mz, DMSO-d_6, ppm) δ 172.10 (C-3), 170.46 (COOH), 162.80 (C-2’), 132.96 (C-6’), 130.57 (C-4’), 127.65 (C-8’), 124.32 (C-7’), 123.01 (C-5’), 121.52 (C-3’), 49.38 (C-1), 34.40 (C-2); FT-IR (KBr, cm^{-1}) 3467, 3170, 3116, 2939, 1755, 1701, 1643, 1593, 1573, 1508, 1434, 1400, 1365, 1346, 1303, 1257, 1222, 1176, 1134, 960, 864, 810, 748, 640. Anal. Calcd for $C_{32}H_{18}N_2O_{12}$: C, 61.74; H, 2.91; N, 4.50. Found: C, 61.67; H, 2.87; N, 4.43.

Synthesis of 2,2'-(1,3,8,10-tetraoxoanthra[2,1,9-def:6,5,10-d'e'f']diisoquinoline-2,9(1*H*,3*H*,8*H*,10*H*)-diyl)bis(3-hydroxypropanoic acid) (3g)

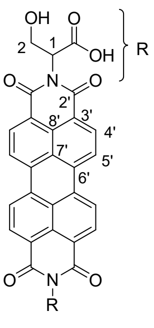

73%, m.p. > 300 ºC; ^{1}H NMR (400 MHz, DMSO-d_6, ppm) δ 14-12 (brs, 2H, COOH), 8.23-8.16 (m, 8H, H-4’ and H-5’), 5.73 (dd, 2H, H-1, J = 9.3 Hz and J = 5.1 Hz), 4.98 (brs, 2H, OH), 4.19-4.15 (m, 4H, H-2); ^{13}C NMR (100 Mz, DMSO-d_6, ppm) δ 169.75 (COOH), 162.40 (C-2’), 133.47 (C-6’), 130.86 (C-4’), 128.10 (C-8’), 124.90 (C-7’), 123.40 (C-5’), 122.06 (C-3’), 58.54 (C-2), 55.28 (C-1); FT-IR (KBr, cm^{-1}): 3436, 2931, 1731, 1693, 1650, 1593, 1573, 1434, 1400, 1365, 1346, 1253, 1176, 1029, 860, 810, 748, 651. Anal. Calcd for $C_{30}H_{18}N_2O_{10}$: C, 63.61; H, 3.20; N, 4.95. Found: C, 63.68; H, 3.26; N, 5.03.

Synthesis of 2,9-bis(2-aminoethyl)anthra[2,1,9-def:6,5,10-d'e'f']diisoquinoline-1,3,8,10(2*H*,9*H*)-tetraone (3h)

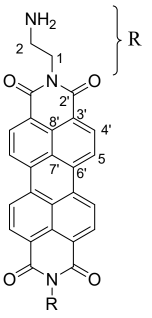

98%, m.p. > 300 ºC, ^{1}H NMR (400 MHz, DMSO-d_6 + TFA, ppm) δ 8.38 (d, 4H, H-5’, J = 8.4 Hz), 8.22 (d, 4H, H-4’, J = 7.6 Hz), 7.88 (brs, 6H, NH_3^+), 4.33 (brs, 4H, H-1), 3.19 (brs, 4H, H-2); ^{13}C NMR (100 MHz, DMSO-d_6 + TFA, ppm) δ 163.35 (C-2’), 133.75 (C-6’), 130.83 (C-4’), 128.21 (C-8’), 125.04 (C-7’), 124.18 (C-5’), 122.49 (C-3’), 37.84 (C-1), 37.84 (C-2); ^{15}N NMR (40.6 MHz, DMSO-d_6 + TFA, ppm) δ 249.93 (N-1’), 30.93 (NH_2). FTIR-ATR (cm^{-1}) 3367, 3304, 2951, 2860, 1687, 1647, 1585, 1506, 1439, 1398, 1336, 1242, 1159, 1082, 1041, 847, 806, 741, 631. Anal. Calcd for $C_{28}H_{20}N_4O_4$: C, 70.58; H, 4.23; N, 11.76. Found: C, 70.43; H, 4.15; N, 11.81.

Synthesis of 2,9-bis(3-aminopropyl)anthra[2,1,9-def:6,5,10-d'e'f']diisoquinoline-1,3,8,10(2*H*,9*H*)-tetraone (3i)

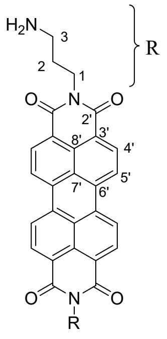

96%, m.p. > 300 ºC; ¹H NMR (400 MHz, DMSO-d_6 + TFA, ppm) δ 8.33 (d, 4H, H-5', J = 8.4 Hz), 8.14 (d, 4H, H-4', J = 7.6 Hz), 7.83 (brs, 4H, NH_2), 4.04 (brs, 4H, H-1), 2.92 (brs, 4H, H-3), 1.97 (brs, 4H, H-2); ¹³C NMR (100 MHz, DMSO-d_6 + TFA, ppm) δ 162.86 (C-2'), 133.26 (C-6'), 130.40 (C-4'), 127.71 (C-8'), 124.52 (C-7'), 123.85 (C-5'), 121.92 (C-3'), 37.48 (C-1 and C-3), 26.29 (C-2); ¹⁵N NMR (40.6 MHz, DMSO-d_6 + TFA, ppm) δ 35.82 (NH_2). FTIR-ATR (cm^{-1}) 3377, 3305, 2951, 2863, 1689, 1645, 1582, 1509, 1438, 1400, 1346, 1212, 1156, 1081, 1047, 877, 806, 731, 639. Anal. Calcd for $C_{30}H_{24}N_4O_4$: C, 71.42; H, 4.79; N, 11.10. Found: C, 71.53; H, 4.85; N, 11.21.

Synthesis of 2,9-bis(4-aminophenyl)anthra[2,1,9-def:6,5,10-d'e'f']diisoquinoline-1,3,8,10(2*H*,9*H*)-tetraone (3j)

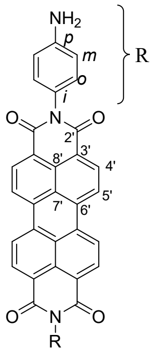

74%, m.p. > 300 ºC. ¹H NMR (400 MHz, DMSO-d_6 + TFA, ppm) δ 8.63 (d, 4H, H-5', J = 8.4 Hz), 8.41 (d, 4H, H-4', J = 8.0 Hz), 7.53 (d, 4H, H*o* + H*o'*, J = 8.4 Hz), 7.44 (d, 4H, H*m* + H*m'*, J = 8.8 Hz), 4.28 (brs, 4H, NH_2); ¹³C NMR (100 MHz, DMSO-d_6 + TFA, ppm) δ 162.99 (C-2'), 134.32 (C*i*), 133.93 (C-6'), 133.79 (C*p*), 130.86 (C-4'), 130.61 (C*o* + C*o'*), 128.63 (C-8'), 125.39 (C-7'), 124.19 (C-5'), 122.91 (C*m* + C*m*'), 122 (C-3'); FTIR-ATR (cm^{-1}) 3381, 3122, 3057, 3037, 2374, 2114, 1757, 1728, 1699, 1653, 1587, 1512, 1437, 1404, 1360, 1292, 1255, 1230, 1180, 1146, 1117, 1016, 933, 862, 808, 727, 648. Anal. Calcd for $C_{36}H_{20}N_4O_4$: C, 75.52; H, 3.52; N, 9.79. Found: C, 75.43; H, 3.65; N, 9.81.

Synthesis of 2,9-bis(3-hydroxypropyl)anthra[2,1,9-def:6,5,10-d'e'f']diisoquinoline-1,3,8,10(2*H*,9*H*)-tetraone (3k)

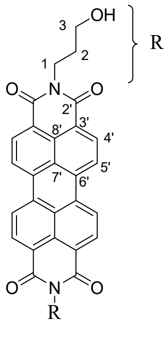

77%, m.p. > 300 ºC; It was not possible to characterize this compound by ¹H and ¹³C NMR due to it poor insolubility in DMSO-d_6. FTIR-ATR (cm^{-1}) 3387, 3313, 2957, 2869, 1683, 1655, 1580, 1533, 1439, 1401, 1346, 1232, 1176, 1047, 870, 811, 733, 665. Anal. Calcd for $C_{30}H_{22}N_2O_6$: C, 71.14; H, 4.38; N, 5.53. Found: C, 71.23; H, 4.50; N, 5.51.

Synthesis of 2,9-bis(1-hydroxybutan-2-yl)anthra[2,1,9-def:6,5,10-d'e'f']diisoquinoline-1,3,8,10(2*H*,9*H*)-tetraone (3l)

84%, m.p. >300 ºC; ¹H NMR (400 MHz, DMSO-d_6, 70 ºC, ppm) δ 8.15 (brs, 8H, H-4' and H-5'), 5.06 (quintet, 2H, H-2, J = 7.1 Hz), 4.64 (s, 2H, OH), 4.08 (t, 2H, H-1, J = 8.9 Hz), 3.87 (t, 2H, H-1, J = 8.9 Hz), 2.09 (qd, 2H, H-3, J = 15.8 Hz and J = 7.1 Hz), 1.95 (qd, 2H, H-3, J = 15.8 Hz and J = 7.1 Hz),

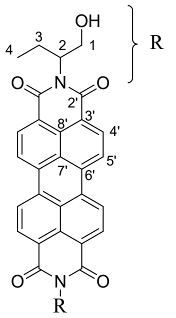

0.97 (t, 6H, H-4, J = 7.5 Hz); ^{13}C NMR (100 MHz, DMSO-d$_6$, 70 ºC, ppm) δ 162.98 (C-2'), 132.83 (C-6'), 130.04 (C-4'), 127.82 (C-8'), 124.53 (C-7'), 122.88 (C-5'), 122.24 (C-3'), 61.15 (C-1), 57.24 (C-2), 21.48 (C-3), 10.88 (C-4); ^{15}N NMR (40.6 MHz, DMSO-d$_6$, 70 ºC, ppm) δ 175.89 (N-1'). FT-IR (KBr, cm^{-1}) 3506, 2966, 2877, 1693, 1650, 1593, 1577, 1434, 1404, 1342, 1253, 1176, 1049, 860, 810, 748. Anal. Calcd for $C_{32}H_{26}N_2O_6$: C, 71.90; H, 4.90; N, 5.24. Found: C, 71.85; H, 4.95; N, 5.16.

Synthesis of 2,9-bis(phenylamino)anthra[2,1,9-def:6,5,10-d'e'f']diisoquinoline-1,3,8,10(2*H*,9*H*)-tetraone (3m)

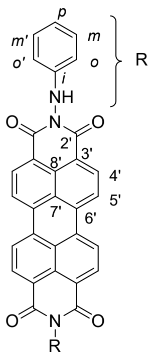

96%, m.p. >300 ºC; ^{1}H NMR (400 MHz, DMSO-d$_6$, ppm) δ 9.00 (d, 4H, H-4' or H-5', J = 8 Hz), 8.68 (brs, 2H, NH), 8.62 (d, 4H, H-4' or H-5', J = 8Hz), 7.17 (t, 4H, H*m* + H*m*', J = 7.2 Hz), 6.8 (d, 4H, H*o* + H*o*', J = 8 Hz), 6.79 (t, 2H, H*p*, J = 7.9 Hz); ^{13}C NMR (100 MHz, DMSO-d$_6$, ppm) δ 162.33 (C-2'), 147.25 (C*i*), 134.48 (C-6'), 131.55 (C-4'), 128.85 (C*m* + C*m*'), 126.05 (C-7' and C-8'), 124.42 (C-5'), 123.21 (C-3'), 119.58 (C*p*), 112.64 (C*o* + C*o*'); FT-IR (KBr, cm^{-1}): 3506, 3055, 1704, 1677, 1593, 1496, 1400, 1353, 1249, 1172, 1056, 964, 856, 810, 744, 698. Anal. Calcd for $C_{36}H_{20}N_4O_4$: C, 75.52; H, 3.52; N, 9.79. Found: C, 75.42; H, 3.38; N, 9.86.

VI. Synthesis and Characterization of compound 4

2,2'-(1,3,8,10-tetraoxoanthra[2,1,9-def:6,5,10-d'e'f']diisoquinoline-2,9(1*H*,3*H*,8*H*,10*H*)-diyl)bis(3-(1*H*-imidazol-1-yl)propanoic acid) (4)

Perylenetetracarboxylic dianhydride (0.12 g; 0.31 mmol) was combined with amino acid **2d** (0.23 g; 0.62 mmol; 2.0 eq.) and imidazole (17 eq.). The reaction mixture was kept at 110 ºC under magnetic stirring for 48 h. The resulting mixture was dissolved in water and the unreacted perylene dianhydride was removed by filtration. The product was precipitated from solution by the dropwise addition of conc. HNO_3. The solid was the filtered and washed with water in order to eliminate the excess of imidazole. The product was dried for 24 h at 100 ºC in a vacuum pistol and identified as compound **4** (0.08 g; 0.12 mmol; 39%).

m.p. > 300 ºC. ^{1}H NMR (400 MHz, DMSO-d$_6$, ppm) δ 8.39-8.28 (m, 10H, H-4', H-5', H-4), 7.20 (s, 2H, H-6), 7.49 (s, 2H, H-7), 6.02 (dd, 2H, H-1, J = 4.8 and J = 8.4 Hz), 5.05 (dd, 2H, H-2, J = 4.8 and J = 14.4 Hz), 4.81 (dd, 2H, H-2, J = 8.4 and J = 14.4 Hz); ^{13}C NMR (100 MHz, DMSO-d$_6$, ppm) δ 168.05 (COOH), 161.64 (CO), 136.63 (C-4), 134.05 (C-6'), 131.09 (C-4'), 127.88 (C-8'), 123.62 (C-5'), 123.46 (C-6), 122.93 (C-7'), 121.50 (C-3'), 121.11 (C-7), 53.16 (C-1), 46.40 and 46.28 (C-2);

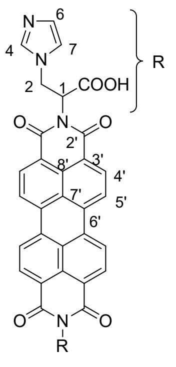

^{15}N NMR (40.6 MHz, DMSO-d_6, ppm) δ 181.6 (N-5), 179.2 (N-1'), 173.3 (N-3); FTIR-ATR (cm^{-1}) 3579, 3145, 2983, 2916, 2848, 1693, 1653, 1587, 1570, 1338, 1248, 1173, 1024, 858, 812, 642. Anal. Calcd for $C_{36}H_{22}N_6O_8$: C, 64.86; H, 3.33; N, 12.61. Found: C, 64.94; H, 3.38; N, 12.55.

VII. Synthesis and Characterization of compounds 9-12

Synthesis of 2,2'-(1,3,8,10-tetraoxoanthra[2,1,9-def:6,5,10-d'e'f']diisoquinoline-2,9(1*H*,3*H*,8*H*,10*H*)-diyl)bis(3-mercaptopropanoic acid) (9)

A mixture of compound **3b** (0.15 g, 0.14 mmol), 200 μL of TFA, and 200 μL triphenylsilane in 1 mL of dichloromethane was stirred overnight at room temperature. The solid was precipitated with ethanol, filtered and washed with ethanol and diethyl ether. The product was dried for 24 h at 100 ºC on a vacuum pistol and identified as compound **9** (0.08 g; 0.13 mmol; 93%).

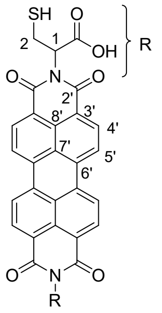

m.p. > 300 ºC; ^1H NMR (400 MHz, DMSO-d_6, 70 ºC, ppm) δ 8.27-8.12 (m, 8H, H-4' and H-5'), 5.71 (dd, 2H, H-1, J = 8.8 Hz and J = 5.2 Hz), 3.51-3.45 (m, 2H, H-2), 3.29-3.21 (m, 2H, H-2), 2.64 (t, 2H, SH, J = 8 Hz); ^{13}C NMR (100 MHz, DMSO-d_6, ppm) δ 169.86 (COOH), 162.18 (C-2'), 133.41 (C-6'), 131.01 (C-4'), 128.06 (C-8'), 124.85 (C-7'), 123.39 (C-5'), 121.64 (C-3'), 55.58 (C-1), 23.02 (C-2); FT-IR (KBr, cm^{-1}): 3487, 2916, 1735, 1697, 1658, 1593, 1573, 1434, 1400, 1361, 1346, 1253, 1176, 1126, 860, 810, 748, 640. Anal. Calcd for $C_{30}H_{18}N_2O_8S_2$: C, 60.19; H, 3.03; N, 4.68; S, 10.71. Found: C, 60.23; H, 3.09; N, 4.73; S, 10.79.

Synthesis of 6,6'-(1,3,8,10-tetraoxoanthra[2,1,9-def:6,5,10-d'e'f']diisoquinoline-2,9(1*H*,3*H*,8*H*,10*H*)-diyl)bis(2-aminohexanoic acid) (10)

Trifluoroacetic acid (500 μL) was added to a suspension of compound **3e** (0.11 g, 0.13 mmol) in DCM (1 mL). After the addition of acid, the solution turned pink. The mixture was stirred at room temperature for 19 h. Precipitation of the product was promoted by addition of 1 mL of diethyl ether. The solid was filtered and washed with ethanol and ethyl ether, dried under vacuum at 100 °C for 24 h and identified as compound **10** (0.07 g, 0.12 mmol, 92%).

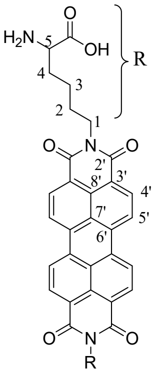

m.p. > 300 ºC; ^{1}H NMR (400 MHz, DMSO-d_6 + TFA, ppm) δ 8.35 (d, 4H, H-5', J = 8.4 Hz), 8.15 (d, 4H, H-4', J = 7.6 Hz), 4.00-3.92 (m, 6H, H-1 and H-5), 1.92-1.80 (m, 4H, H-4), 1.72-1.67 (m, 4H, H-2), 1.56-1.39 (m, 4H, H-3); ^{13}C NMR (100 MHz, DMSO-d_6 + TFA, ppm) δ 171.15 (COOH), 162.44 (C-2'), 133.08 (C-6'), 130.43 (C-4'), 127.82 (C-8'), 124.48 (C-7'), 123.79 (C-5'), 122.09 (C-3'), 51.99 (C-5), 39.50 (C-1), 29.87 (C-4), 27.09 (C-2), 22.07 (C-3); FT-IR (KBr, cm^{-1}) 3506, 3444, 3093, 2931, 2866, 1689, 1650, 1593, 1574, 1504, 1442, 1384, 1353, 1253, 1172, 1130, 1091, 1041, 852, 810, 744, 632. Anal. Calcd for $C_{36}H_{32}N_4O_8$: C, 66.66; H, 4.97; N, 8.64. Found: C, 66.54; H, 4.90; N, 8.58.

Synthesis of (*Z*)-6,6'-(1,3,8,10-tetraoxoanthra[2,1,9-def:6,5,10-d'e'f']diisoquinoline-2,9(1*H*,3*H*,8*H*,10*H*)-diyl)bis(2-((*Z*)-3-carboxyacrylamido)hexanoic acid) (11)

Maleic anhydride (0.20 g, 2.06 mmol) and triethylamine (230 µL, 1.66 mmol) were added to a mixture of compound **10** (0.72 g, 0.83 mmol) in dimethylsulfoxide (1 mL). The reaction mixture was stirred at room temperature for 5 h. The product was precipitated from solution by addition of distilled water and a few drops of concentrated nitric acid. The solid was filtered and washed thoroughly with distilled water. The product was dried under vacuum at 100 °C overnight and identified as compound **11** (0.68 g; 0.80 mmol, 96%).

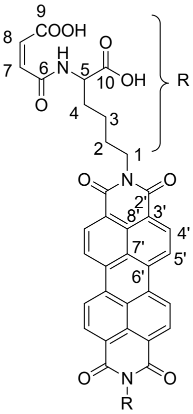

m.p. > 300 ºC; ^{1}H NMR (400 MHz, DMSO-d_6, 80 ºC, ppm) δ 9.36 (brs, 2H, NH), 7.41-7.33 (m, 8H, H-4' and H-5'), 6.47 (d, 2H, H-8, J = 12 Hz), 6.29 (d, 2H, H-7, J = 12 Hz), 4.34-4.33 (m, 2H, H-5), 3.73-3.43 (m, 4H, H-1), 2.0-1.76 (m, 4H, H-4), 1.75-1.56 (m, 4H, H-2), 1.50-1.30 (m, 4H, H-3); ^{13}C NMR (100 MHz, DMSO-d_6, 80 ºC, ppm) δ 172.84 (C-10), 166.03 (C-9), 161.62 (C-2'), 133.40 (C-7), 131.92 (C-6'), 130.28 (C-8), 129.19 (C-4'), 126.50 (C-8'), 123.19 (C-7'), 122.74 (C-5'), 120.82 (C-3'), 52.70 (C-5), 40.41 (C-1), 30.64 (C-4), 26.93 (C-2), 23.05 (C-3); FT-IR (KBr, cm^{-1}) 3503, 3056, 2954, 1693, 1650, 1594, 1576, 1507, 1443, 1402, 1348, 1248, 1166, 855, 809, 746, 628. Anal. Calcd for $C_{44}H_{36}N_4O_{14}$: C, 62.56; H, 4.30; N, 6.63. Found: C, 62.49; H, 4.28; N, 6.60.

Synthesis of 6,6'-(1,3,8,10-tetraoxoanthra[2,1,9-def:6,5,10-d'e'f']diisoquinoline-2,9(1*H*,3*H*,8*H*,10*H*)-diyl)bis(2-(2,5-dioxo-2,5-dihydro-1*H*-pyrrol-1-yl)hexanoic acid) (12)

Compound **11** (0.19 g, 0.22 mmol) was combined with 5 ml of toluene, trimethylamine (160 µL, 1.2 mmol) and maleic anhydride (0.02 g, 0.22 mmol) (fractionated addition: 0.11 mmol at beginning and 0.11 mmol after 1.5 h). After 3 h under reflux the solvent was removed and addition of approximately 1 ml of dimethyl sulfoxide led to a solid suspension. The insoluble impurities were eliminated by filtration and washed with 1 ml of dimethyl sulfoxide. Approximately 3 ml of aqueous 2M HCl (pre-cooled in an ice bath) were added the dimethyl sulfoxide solution leading to a solid product. The product was filtered, washed with a copious amount of distilled water, dried under vacuum at 60 ºC for 24 h and identified as compound **12** (0.13 g; 0.16 mmol, 73%).

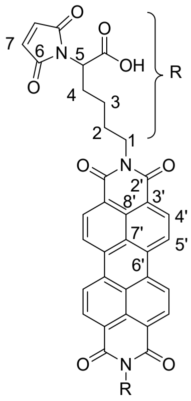

m.p. > 300 ºC; ^{1}H NMR (400 MHz, DMSO-d$_6$, 80 ºC, ppm) δ 7.67-7.18 (m, 8H, H-4' and H-5'), 6.96 (s, 4H, H-7), 4.56-4.52 (m, 2H, H-5), 3.90-3.77 (m, 4H, H-1), 2.14-2.09 (m, 4H, H-4), 1.71-1.57 (m, 4H, H-2), 1.41-1.28 (m, 4H, H-3); ^{13}C NMR (100 MHz, DMSO-d$_6$, 80 ºC, ppm) δ 170.23 (COOH), 170.18 (C-6), 161.62 (C-2'), 134.34 (C-7), 132.12 (C-6'), 129.35 (C-4'), 127.70 (C-8'), 122.60 (C-5'), 123.86 (C-7'), 121.11 (C-3'), 39.08 (C-1), 27.61 (C-4), 26.47 (C-2), 23.77 (C-3); FT-IR (KBr, cm^{-1}) 3508, 3045, 2956, 1691, 1649, 1592, 1577, 1441, 1401, 1343, 1252, 1166, 1092, 1017, 856, 830, 809, 745, 695, 627. Anal. Calcd for $C_{44}H_{32}N_4O_{12}$: C, 65.34; H, 3.99; N, 6.93. Found: C, 65.45; H, 4.08; N, 7.06.

VIII. Synthesis and Characterization of compounds 14-17

Synthesis of 4-((9,10-dioxo-9,10-dihydroanthracen-2-yl)amino)-4-oxobutanoic acid (14)

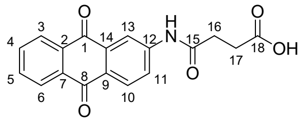

A mixture of 2-aminoanthraquinone **13** (10.15 g, 45.5 mmol) and a soloution of succinic anhydride (1.05 eq.) in 37 mL of acetic acid, was refluxed for 1 h. The reaction mixture was cooled down to room temperature and the solid suspension was filtered, washed repeatedly with ethyl ether and left to dry overnight at 100 ºC under vacuum. A dark green solid was isolated and identified as compound **14** (11.70 g, 36 mmol, 79 %).
m.p. 228-230 ºC; ^{1}H NMR (400 MHz, DMSO-d$_6$, ppm) δ 12.16 (s, 1H, COOH), 10.62 (s, 1H, NH), 8.43 (d, 1H, AQ-H-13, J = 2.1 Hz), 8.20-8.15 (m, 2H, AQ-H-3 and AQ-H-6), 8.12 (d, 1H, AQ-H-10, J = 8.8 Hz), 8.04 (dd, 1H, AQ-H-11, J = 8.8 Hz and J = 2.1 Hz), 7.94-7.85 (m, 2H, AQ-H-4 and AQ-H-5), 2.66 (t, 2H, H-17, J = 6.3 Hz), 2.57 (t, 2H, H-16, J = 6.3 Hz); ^{13}C NMR (100 MHz, DMSO-d$_6$, ppm) δ 182.40 (AQ-1-CO), 181.28 (AQ-8-CO), 173.69 (COOH), 171.17 (CO-15), 144.67 (AQ-12), 134.13 (AQ-14), 134.52 (AQ-4 or AQ-5), 134.16 (AQ-4 or AQ-5), 133.11 (AQ-2 or AQ-7), 133.07 (AQ-2 or AQ-7), 128.47 (AQ-10), 127.68 (AQ-9), 126.69 (AQ-3 or AQ-6), 126.61 (AQ-3 or AQ-6), 123.53 (AQ-11), 115.60 (AQ-13), 31.27 (C-17), 28.56 (C-16); FT-IR (KBr, cm^{-1}) 3346, 1868, 1660, 1587, 1528, 1461, 1408, 1376, 1337, 1294, 1228, 1171, 1153, 932, 919, 864, 715. Anal. Calcd for $C_{18}H_{13}NO_5$: C, 66.87; H, 4.05; N, 4.33. Found: C, 66.91; H, 4.11; N, 4.35.

Synthesis of 1-(9,10-dioxo-9,10-dihydroanthracen-2-yl)pyrrolidine-2,5-dione (15)

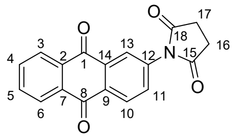

Compound **14** (1.00 g, 3.10 mmol) was refluxed in 4 mL of acetic anhydride for 50 min.. The green solid that precipitated after cooling to room temperature was washed with an abundant amount of diethyl ether. The product was dried under vacuum at 100 ºC for 15 h and was identified as compound **15** (0.78 g, 2.56 mmol, 83%).

m.p. 290-294 °C; ^{1}H NMR (400 MHz, DMSO-d_6, ppm) δ 8.30 (d, 1H, AQ-H-10, J = 8.3 Hz), 8.21-8.17 (m, 2H, AQ-H-3 and AQ-H-6), 8.16 (d, 1H, AQ-13-H, J = 2.1 Hz), 7.95-7.88 (m, 2H, AQ-H-5 and AQ-H-4), 7.83 (dd, 1H, AQ-H-11, J = 8.3 Hz and J = 2.1 Hz), 2.83 (s, 4H, H-16 and H-17); ^{13}C NMR (100 MHz, DMSO-d_6, ppm) δ 181.99 (AQ-1-CO), 181.87 (AQ-8-CO), 176.61 (CO-15 and CO-18), 134.68 (AQ-5 or AQ-4), 134.58 (AQ-5 or AQ-4), 133.67 (AQ-14), 133.04 (AQ-2 and AQ-7), 132.36 (AQ-11), 132.10 (AQ-9), 127.72 (AQ-10), 126.84 (AQ-6 or AQ-3), 126.82 (AQ-6 or AQ-3), 124.78 (AQ-13), 28.68 (C-17 and C-18); FT-IR (KBr, cm^{-1}) 1707, 1676, 1590, 1530, 1490, 1461, 1378, 1332, 1294, 1178, 997, 854, 819, 709, 670. Anal. Calcd for $C_{18}H_{11}NO_4$: C, 70.82; H, 3.63; N, 4.59. Found: C, 70.80; H, 3.79; N, 4.53.

Synthesis of *N*^{1}-(2-aminoethyl)-*N*^{4}-(9,10-dioxo-9,10-dihydroanthracen-2-yl)succinamide (16)

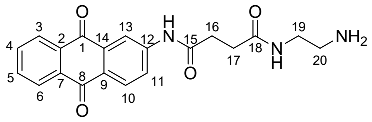

Ethylenediamine (110 µL, 1.2 eq.) was added to a suspension of **15** (0.50 g, 1.65 mmol) in 10 mL of THF. The mixture was stirred for 24 h at room temperature. The precipitate was then filtered and washed with THF and diethyl ether. The green solid was dried under vacuum overnight at 100 ºC and identified as compound **16** (0.31 g, 0.85 mmol, 52%).

m.p. 204-206 °C; ^{1}H NMR (400 MHz, DMSO-d_6 + TFA, ppm) δ 10.63 (s, 1H, NH), 8.47 (d, 1H, AQ-H-13, J = 2 Hz), 8.20-8.14 (m, 2H, AQ-H-6 and AQ-H-3), 8.13 (d, 1H, AQ-H-10, J = 8.6 Hz), 8.05 (dd, 1H, AQ-H-11, J = 8.6 Hz and J = 2 Hz), 7.92-7.85 (m, 3H, AQ-H-5, AQ-H-4 and NH), 3.07-3.02 (m, 2H, H-19), 2.64 (t, 2H, H-17, J = 7.2 Hz), 2.55 (t, 2H, H-20, J = 6.4 Hz), 2.46 (t, 2H, H-16, J = 7.2 Hz). After the addition of few drops of TFA into the NMR tube appears in the ^{1}H NMR spectra a broad singlet at 7.72 ppm that corresponds to the protonated amine ($NH_3^+ \ ^-OOCF_3C$). ^{13}C NMR (100 MHz, DMSO-d_6 + TFA, ppm) δ 182.53 (AQ-1-CO), 181.37 (AQ-8-CO), 171.70 (AQ-12), 171.26 (CO-15), 144.84 (C-17), 134.59 (AQ-4 or AQ-5), 134.23 (AQ-4 or AQ-5), 134.17 (AQ-14), 133.18 (AQ-2 and AQ-7), 133.14 (AQ-2 and AQ-7), 128.51 (AQ-10), 127.70 (AQ-9), 126.75 (AQ-3 or AQ-6), 126.67 (AQ-3 or AQ-6), 123.64 (AQ-11), 115.70 (AQ-13), 41.42 (C-19), 40.85 (C-20), 31.88 (C-17), 30.06 (C-16); FT-IR (KBr, cm^{-1}) 3380, 1692, 1659, 1587, 1533, 1461, 1377, 1348, 1299, 1238, 1173, 1148, 994, 933, 869, 719. Anal. Calcd for $C_{20}H_{19}N_3O_4$: C, 65.74; H, 5.24; N, 11.50. Found: C, 65.79; H, 5.21; N, 11.65.

Synthesis of *N*[1]-(3-aminopropyl)-*N*[4]-(9,10-dioxo-9,10-dihydroanthracen-2-yl)succinamide (17)

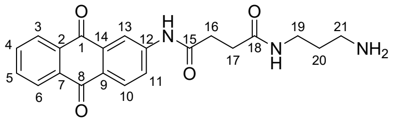

1,3-Diaminopropane (120 µL; 1.1 eq.) was added to a suspension of **15** (0.50 g, 1.65 mmol) in THF (10 mL) and the mixture was stirred for 24 h at room temperature. The precipitate was then filtered and washed with THF and diethyl ether. The green solid was dried under vacuum overnight at 100 ºC and identified as compound **17** (0.31 g, 0.82 mmol, 51%).

m.p. 193-196 ºC; ^{1}H NMR (400 MHz, DMSO-d_6 + TFA, ppm) δ 10.61 (s, 1H, NH), 8.45 (brs, 1H, AQ-H-13), 8.18-8.16 (m, 2H, AQ-H-6 and AQ-H-3), 8.13 (d, 1H, AQ-H-10, J = 8.4 Hz), 8.03 (dd, 1H, AQ-H-11, J = 8.4 Hz and J = 2 Hz), 7.91-7.85 (m, 3H, AQ-H-5, AQ-H-4 and NH), 3.1-3.02 (m, 2H, H-19), 2.63 (t, 2H, H-17, J = 6.8 Hz), 2.49 (t, 2H, H-21, J = 3.6 Hz), 2.43 (t, 2H, H-16, J = 6 Hz and J = 6.8 Hz), 1.46 (t, 2H, H-20, J = 6 Hz and J = 6.8 Hz). After the addition of few drops of TFA into the NMR tube appears in the ^{1}H NMR spectra a broad singlet at 7.72 ppm that corresponds to the protonated amine (NH_3^+ $^-OOCF_3C$). ^{13}C NMR (100 MHz, DMSO-d_6 + TFA, ppm) δ 182.48 (AQ-1-CO), 181.32 (AQ-8-CO), 171.61 (CO-15), 170.92 (CO-18), 144.79 (AQ-12), 134.17 (AQ-4 or AQ-5), 134.13 (AQ-4 or AQ-5), 134.13 (AQ-14), 133.14 (AQ-2 and AQ-7), 133.1 (AQ-2 and AQ-7), 128.5 (AQ-10), 127.65 (AQ-9), 126.7 (AQ-3 or AQ-6), 126.6 (AQ-3 or AQ-6), 123.57 (AQ-11), 115.60 (AQ-13), 38.87 (C-21), 36.20 (C-19), 33.72 (C-20), 31.86 (C-17), 29.98 (C-16); FT-IR (KBr, cm^{-1}) 713, 1098, 1302, 1331, 1418, 1543, 1590, 1641, 1671, 1697, 3288, 3339. Anal. Calcd for $C_{21}H_{21}N_3O_4$: C, 66.48; H, 5.58; N, 11.08. Found: C, 66.59; H, 5.61; N, 11.15.

IX. Synthesis and Characterization of compounds 18 and 19

Synthesis of *N*[1],*N*[1']-(((2,2'-(1,3,8,10-tetraoxoanthra[2,1,9-def:6,5,10-d'e'f']diisoquinoline-2,9(1*H*,3*H*,8*H*,10*H*)-diyl)bis(3-phenylpropanoyl))bis(azanediyl))bis(ethane-2,1-diyl))bis(*N*4-(9,10-dioxo-9,10-dihydroanthracen-2-yl)succinamide) (18)

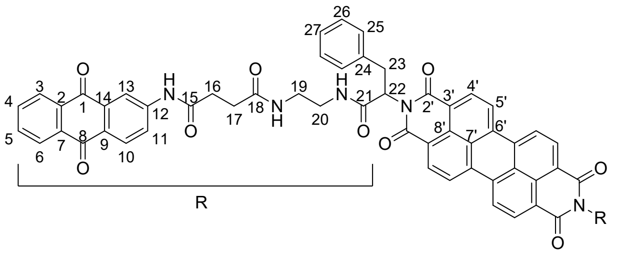

Compound **3d** (0.15 g, 0.22 mmol) was dissolved in 1 mL of DMF and stirred in an ice bath for 10 min. Hydroxybenzotriazole (0.06 g, 0.46 mmol, 2.05 eq.), *N,N'*-dicyclohexylcarbodiimide (0.09 g, 0.45 mmol, 2.05 eq.) and compound **7** (0.16 g, 0.45 mmol, 2.05 eq.) were added to the mixture with a 10 min delay between both reagents. The mixture was stirred for 24 h at room temperature and cooled to -18 ºC to promote

the precipitation of urea. The solid suspension was removed by filtration and washed with 1 mL of cold DMF. Acetone was added to the solution and the pink solid was filtered and washed with acetone and diethyl ether. The solid was dried under vacuum at 100 ºC for 15 h and identified as compound **18** (0.11 g, 0.08 mmol, 36%).

m.p. 223 °C – dec; ^{1}H NMR (400 MHz, DMSO-d_6, ppm) δ 10.40 (s, 2H, AQ-NH), 8.15-7.81 (m, 14H, AQ-H-3, AQ-H-6, AQ-H-13, H-5’, 2xNH), 7.74-7.5 (m, 12H, AQ-H-10, AQ-H-11, AQ-H-4 and AQ-H-5, H-4’), 7.24 (d, 4H, H-25, J = 7.2 Hz), 7.18 (t, 4H, H-26, J = 7.2 Hz), 7.07 (t, 2H, H-27, J = 7.2 Hz), 5.87 (dd, 2H, H-22, J = 9.2 Hz and J = 6 Hz), 3.70 (dd, 2H, H-23, J = 15 Hz and J = 6 Hz), 3.41-3.30 (m, 2H, H-23), 3.26-3.09 (m, 8H, H-19 and H-20), 2.54 (brs, 4H, H-16 or H-17), 2.37 (t, 4H, H-16 or H-17, J = 6 Hz); ^{13}C NMR (100 MHz, DMSO-d_6, ppm) δ 181.86 (AQ-1-CO), 180.77 (AQ-8-CO), 171.61 (CO-15 or CO-18), 171.55 (CO-15 or CO-18), 168.64 (CO-21), 162.52 (CO-2’), 144.26 (AQ-12), 138.25 (C-24), 134.27 (AQ-4 or AQ-5), 133.96 (AQ-4 or AQ-5), 133.51 (AQ-14), 133.22 (C-6’), 132.64 (AQ-2 or AQ-7), 132.57 (AQ-2 or AQ-7), 130.60 (C-4’), 129.07 (C-25), 128.16 (C-26), 128.04 (C-8’), 127.17 (AQ-9), 126.37 (C-27), 126.31 (AQ-3 and AQ-6), 124.86 (C-7’), 123.19 (C-5’), 123.14 (AQ-11), 122.17 (C-3’), 115.22 (AQ-13), 55.00 (C-22), 39.08 (C-19 or C-20), 38.22 (C-19 or C-20), 33.97 (C-23), 31.85 (C-16 or C-17), 30.35 (C-16 or C-17); FT-IR (KBr, cm^{-1}) 3374, 1697, 1661, 1591, 1534, 1402, 1363, 1340, 1295, 1252, 1171, 995, 855, 810, 746, 715. Anal. Calcd for $C_{82}H_{60}N_8O_{14}$: C, 71.30; H, 4.38; N, 8.11. Found: C, 71.33; H, 4.35; N, 8.10.

Synthesis of *N^1,$N^{1'}$*-(((2,2'-(1,3,8,10-tetraoxoanthra[2,1,9-def:6,5,10-d'e'f']diisoquinoline-2,9(1*H*,3*H*,8*H*,10*H*)-diyl)bis(3-phenylpropanoyl))bis(azanediyl))bis(propane-3,1-diyl))bis(*N*4-(9,10-dioxo-9,10-dihydroanthracen-2-yl)succinamide) (19)

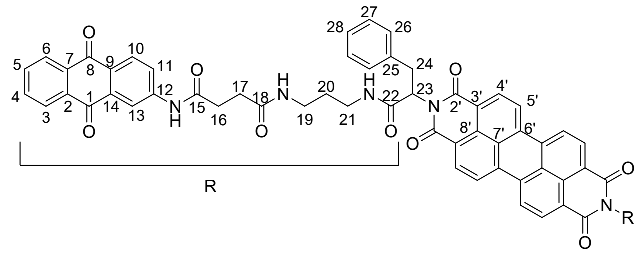

Compound **3d** (0.15 g, 0.22 mmol) was dissolved in 1 mL of DMF and stirred in an ice bath for 10 min. Hydroxybenzotriazole (0.06 g, 0.46 mmol, 2.05 eq.), *N,N'*-dicyclohexylcarbodiimide (0.09 g, 0.45 mmol, 2.05 eq.) and compound **17** (0.17 g, 0.45 mmol, 2.05 eq.) were added to the mixture with a 10 min delay between both reagents. The mixture was stirred for 24 h at room temperature and then cooled to -18 ºC in to promote the precipitation of urea. The solid suspension was removed by filtration and washed with 1 mL of cold DMF. Acetone was added to the solution and the pink solid was filtered and washed with acetone and diethyl ether. The solid was dried under vacuum at 100 ºC for 15 h and identified as compound **19** (0.15 g, 0.11 mmol, 50%).

m.p. 215 °C – dec; ^{1}H NMR (400 MHz, DMSO-d_6, ppm) δ 10.40 (s, 2H, AQ-NH), 8.20-7.88 (m, 14H, AQ-H-3, AQ-H-6, AQ-H-13, 2xNH, H-5’), 7.87-7.65 (m, 12H, AQ-H-10, AQ-H-11, AQ-H-4 and AQ-H-5, H-4’),

7.24 (d, 4H, H-26, J = 7.2 Hz), 7.16 (t, 4H, H-27, J = 7.2 Hz), 7.08 (t, 2H, H-28, J =7.2 Hz), 5.9-5.8 (m, 2H, H-23), 3.7-3.6 (m, 2H, H-24), 3.40-3.32 (m, 2H, H-24), 3.33-3.00 (m, 8H, H-19 and H-21), 2.57 (brs, 4H, H-16 or H-17), 2.40 (brs, 4H, H-16 or H-17), 1.60 (brs, 4H, H-20); ^{13}C NMR (100 MHz, DMSO-d_6, ppm) δ 180.83 (AQ-1-CO), 181.86 (AQ-8-CO), 171.45 (CO-15 or CO-18), 171.19 (CO-15 or CO-18), 168.21 (CO-22), 162.47 (CO-2'), 144.22 (AQ-12), 138.27 (C-25), 134.28 (AQ-14), 133.98 (AQ-4 and AQ-5), 133.22 (C-6'), 130.49 (C-4'), 129.09 (C-26), 128.14 (C-27), 128.04 (C-8'), 127.92 (AQ-10), 127.14 (AQ-9), 126.39 (AQ-3 or AQ-6 and C-28), 126.34 (AQ-3 or AQ-6), 124.86 (C-7'), 123.00 (AQ-11 and C-5'), 122.17 (C-3'), 115.21 (AQ-13), 54.97 (C-23), 37.10 (C-19 or C-21), 36.51 (C-19 or C-21), 33.98 (C-24), 31.65 (C-16 or C-17), 29.82 (C-16 or C-17), 28.97 (C-20); FT-IR (KBr, cm^{-1}) 3378, 1698, 1660, 1591, 1535, 1402, 1340, 1295, 1252, 1170, 994, 855, 810, 746, 715. Anal. Calcd for $C_{84}H_{64}N_8O_{14}$: C, 71.58; H, 4.58; N, 7.95. Found: C, 71.43; H, 4.65; N, 8.11.

X. NMR spectra of products

(2-{[(5-amino-5-carboxypentyl)amino]methyl}cyclopenta-2,4-dien-1-id-1-yl)(cyclopenta-2,4-dien-1-id-1-yl)ironbis(ylium) 2c

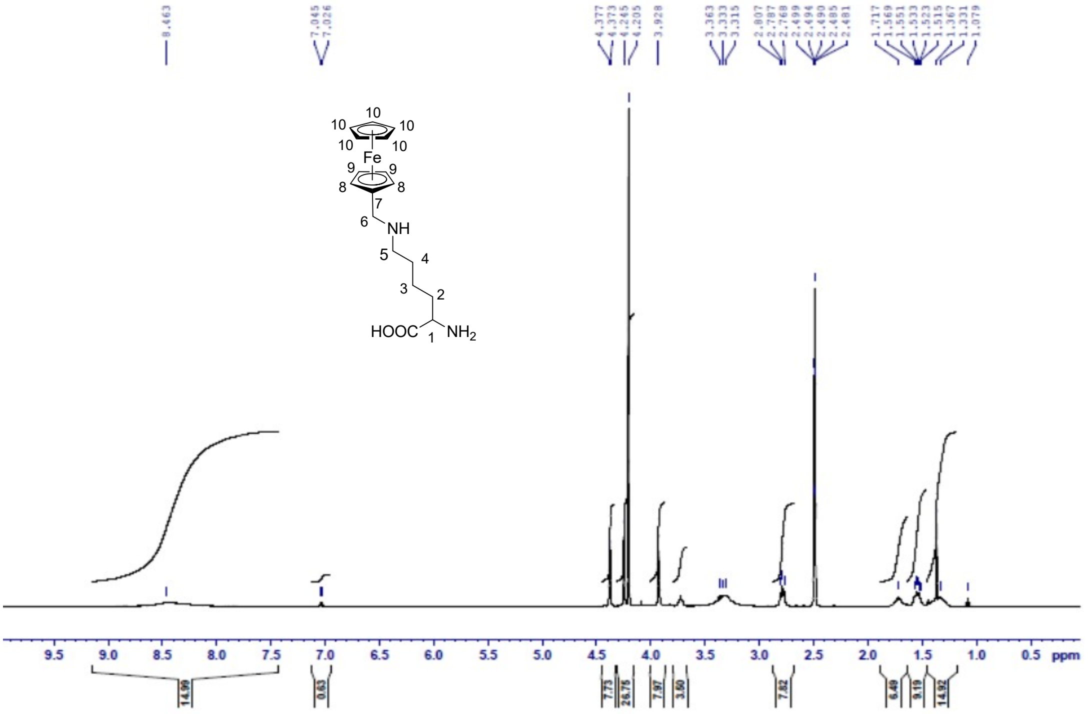

Figure 1. ^{1}H NMR spectrum of **2c**.

2,2'-(1,3,8,10-tetraoxo-1,3,8,10-tetrahydroanthra[2,1,9-def:6,5,10-d'e'f']diisoquinoline-2,9-diyl)bis(3-phenylpropanoic acid) (3a)

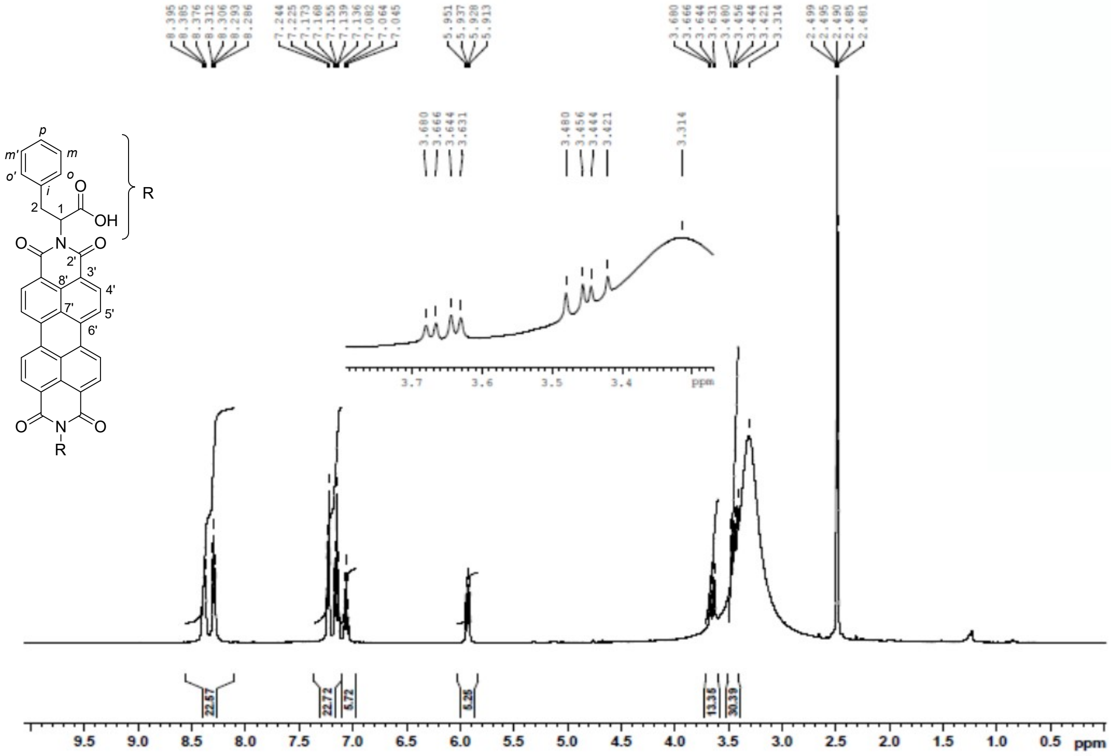

Figure 2. ^{1}H NMR correlation spectrum of **3a**.

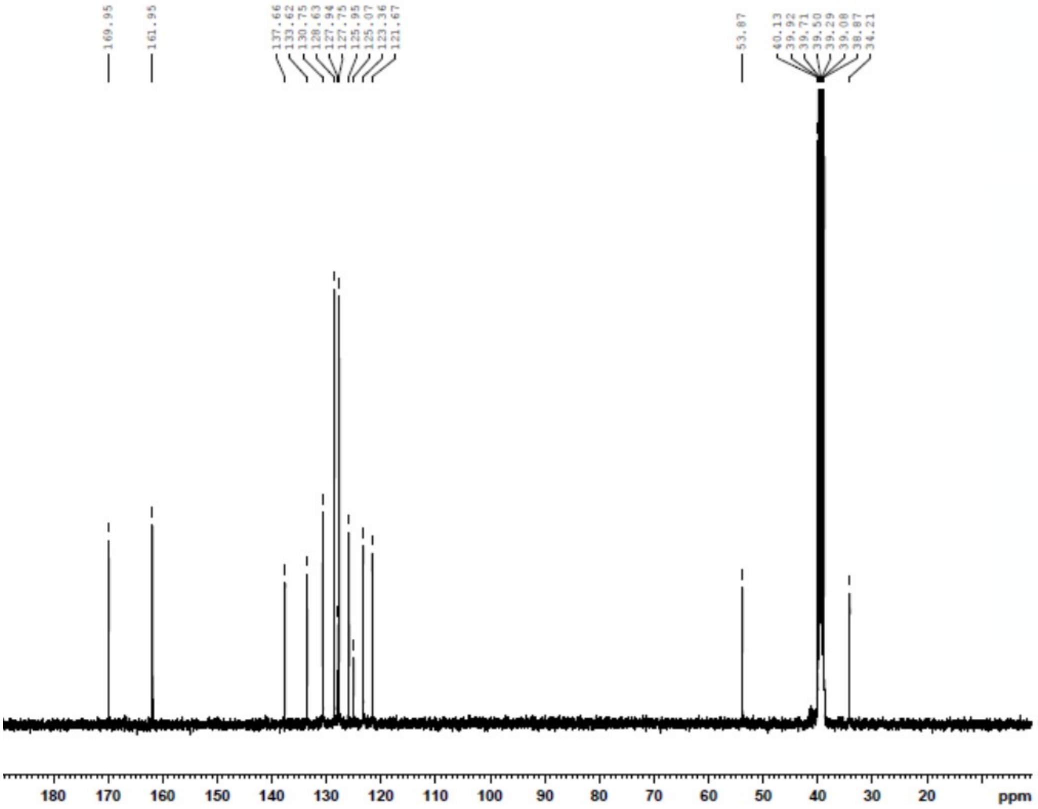

Figure 3. ^{13}C NMR spectrum of **3a**.

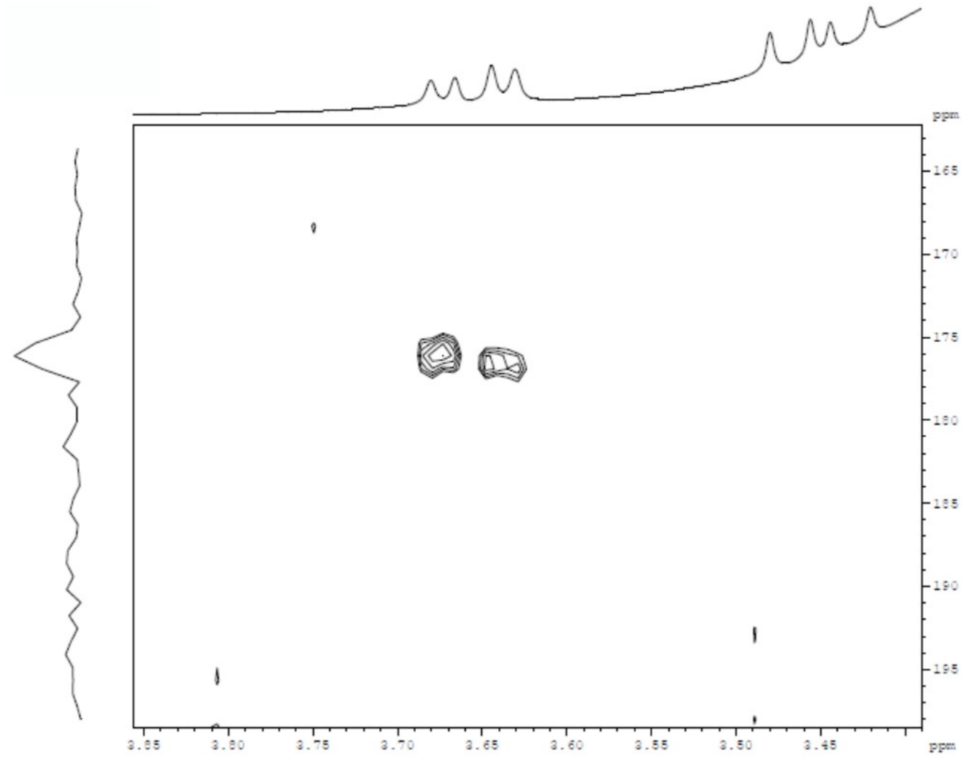

Figure 4. ^{15}N-^{1}H NMR correlation spectrum of **3a**.

6,6'-(1,3,8,10-tetraoxoanthra[2,1,9-def:6,5,10-d'e'f']diisoquinoline-2,9(1*H*,3*H*,8*H*,10*H*)-diyl)bis(2-((tert-butoxycarbonyl)amino)hexanoic acid) (3b)

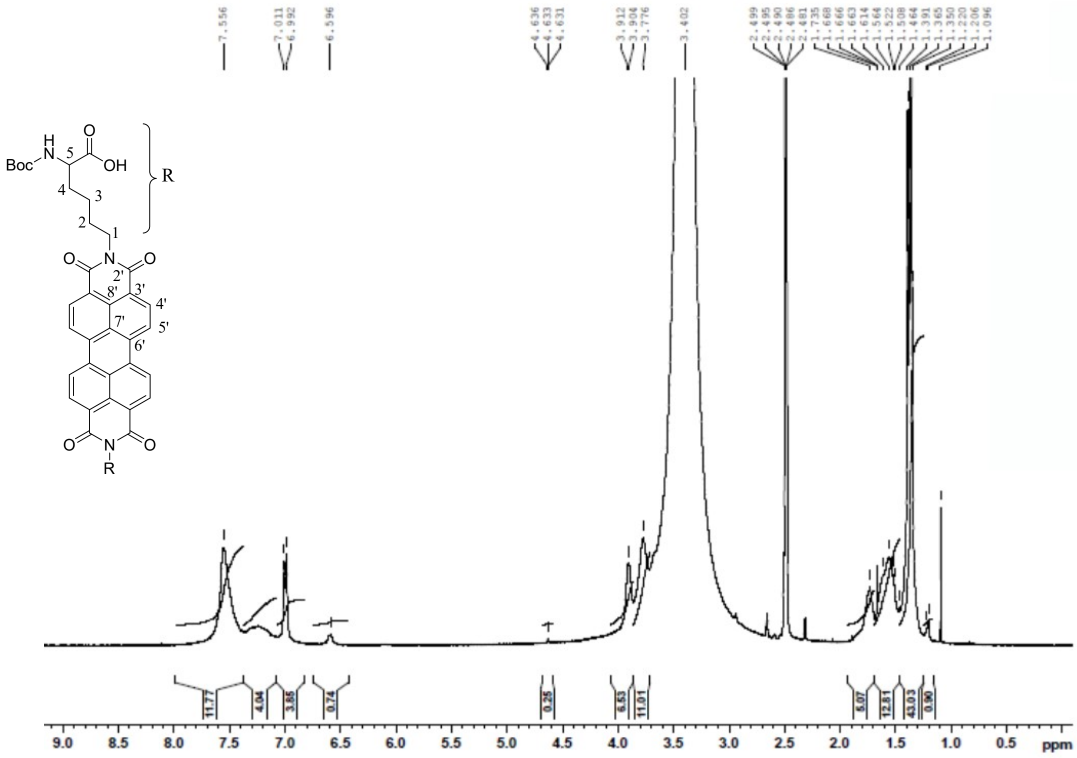

Figure 5. ^{1}H NMR spectrum of **3b**.

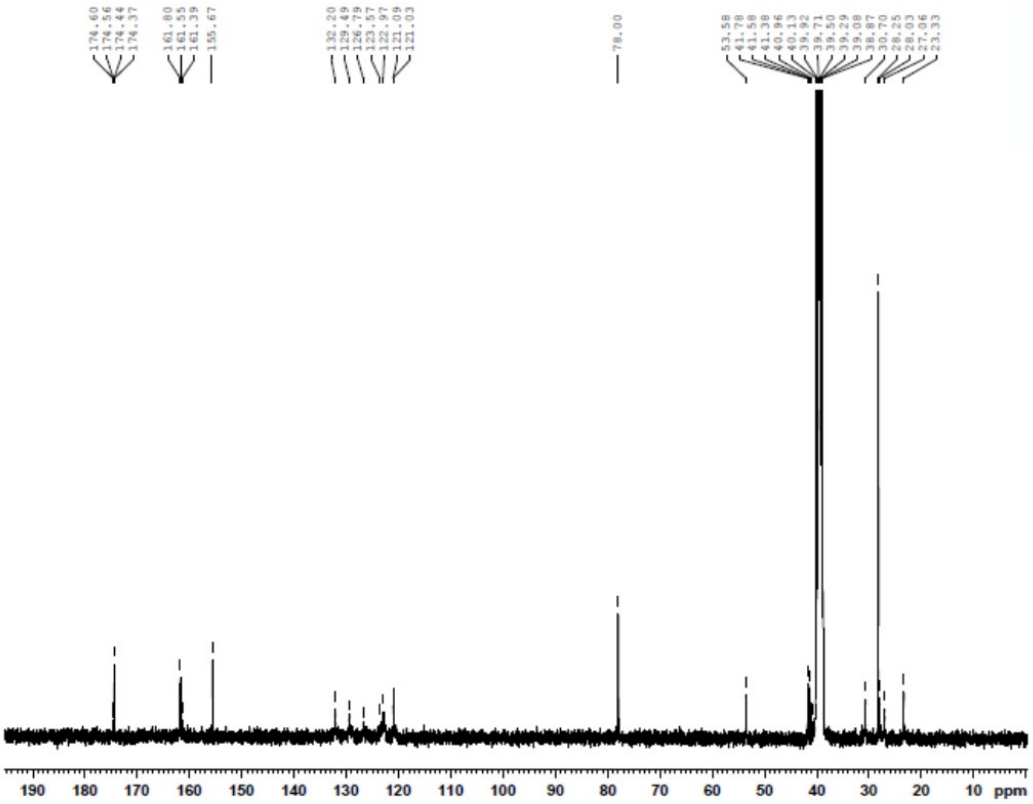

Figure 6. ^{13}C NMR spectrum of **3b**.

[2-({[5-carboxy-5-(18-{1-carboxy-5-[({2-[(cyclopenta-2,4-dien-1-id-1-yl)ferriobis(ylium)] cyclopenta-1(5),3-dien-2-id-1-yl}methyl)amino]pentyl}-6,8,17,19-tetraoxo-7,18-diazaheptacyclo [14.6.2.22,5.03,12.04,9.013,23.020,24]hexacosan-7-yl)pentyl]amino}methyl)cyclopenta-2,4-dien-1-id-1-yl](cyclopenta-2,4-dien-1-id-1-yl)ironbis(ylium (3c)

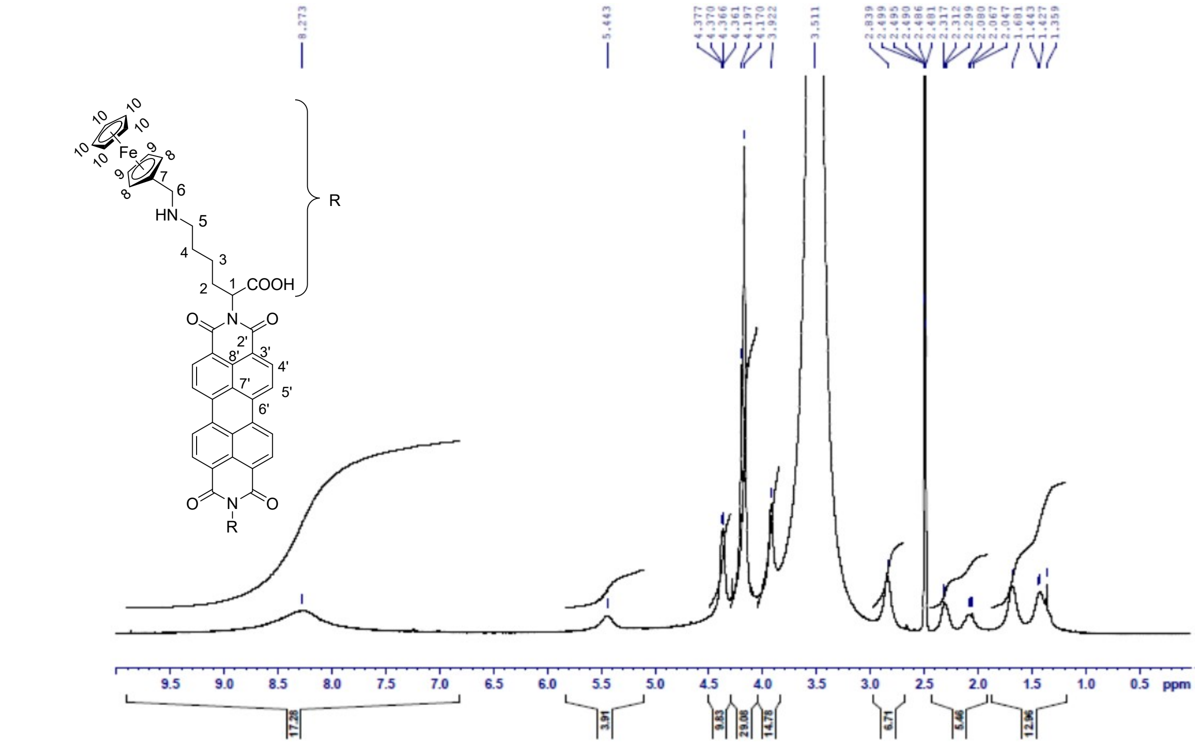

Figure 7. ^{1}H NMR spectrum of **3c**.

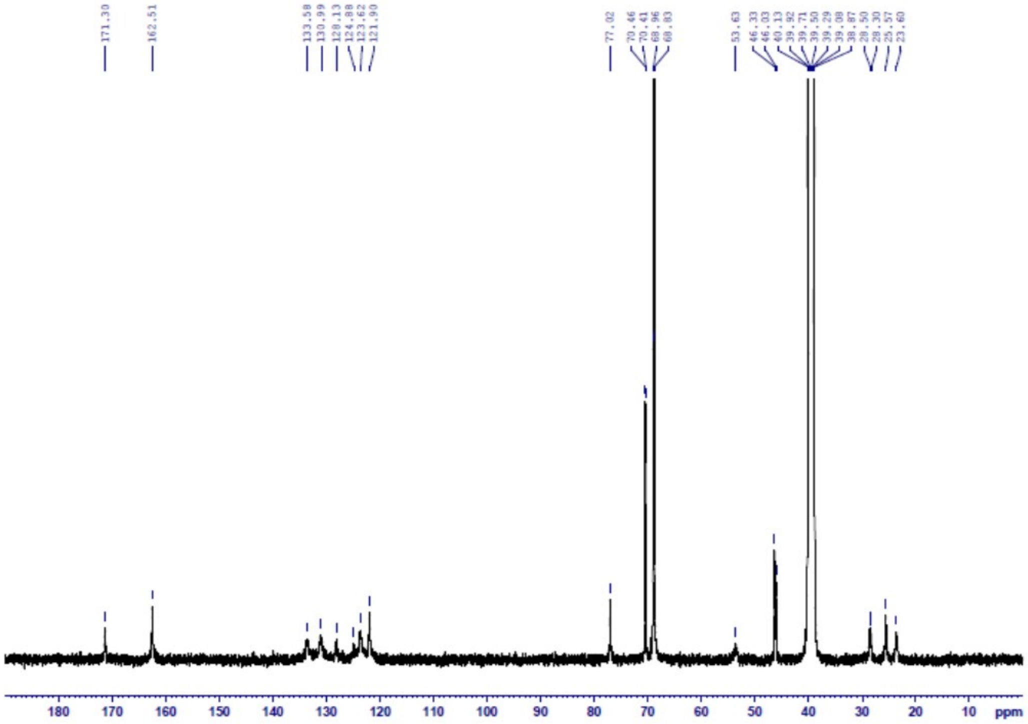

Figure 8. ^{13}C NMR spectrum of **3c**.

2,2'-(1,3,8,10-tetraoxoanthra[2,1,9-def:6,5,10-d'e'f']diisoquinoline-2,9(1*H*,3*H*,8*H*,10*H*)-diyl)bis(3-(tritylthio)propanoic acid) (3d)

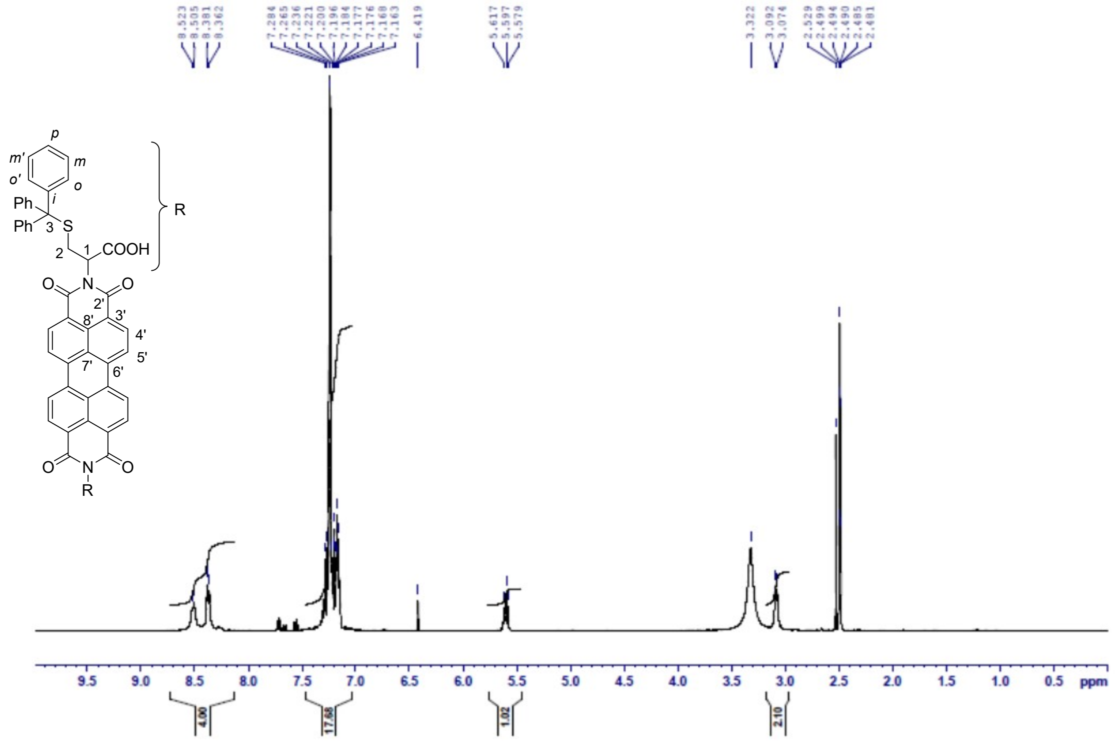

Figure 9. ^{1}H NMR spectrum of **3d**.

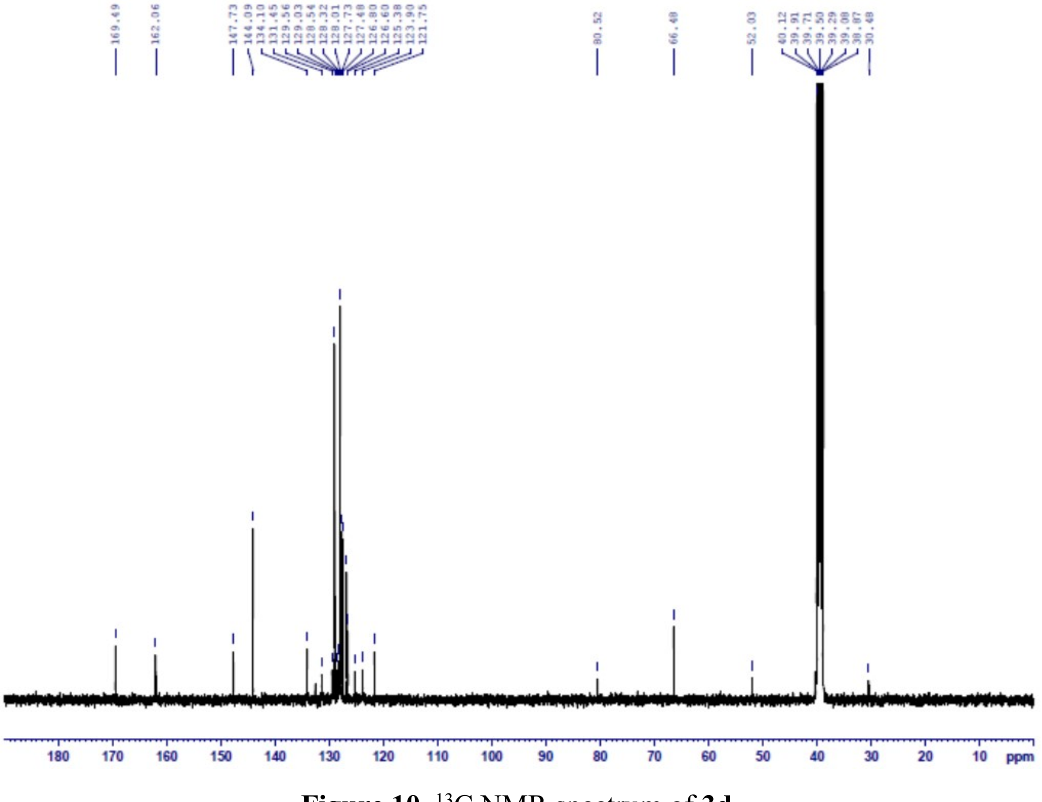

Figure 10. ^{13}C NMR spectrum of **3d**.

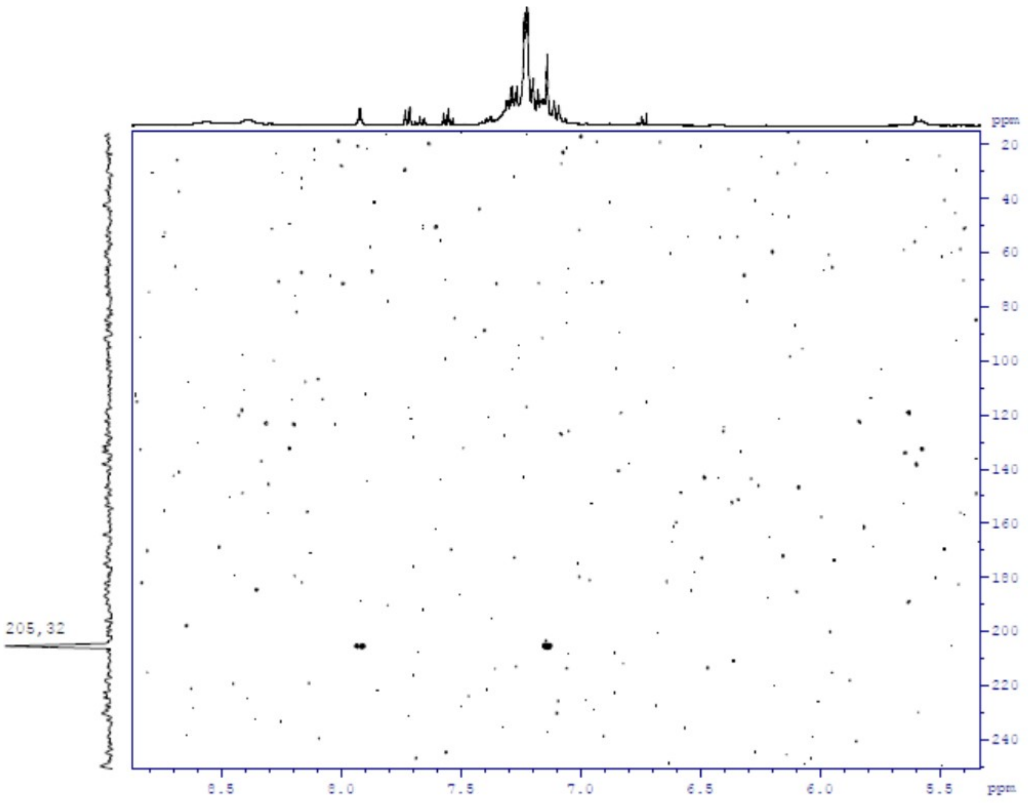

Figure 11. ^{15}N-^{1}H NMR correlation spectrum of **3d**.

2,2'-(1,3,8,10-tetraoxoanthra[2,1,9-def:6,5,10-d'e'f']diisoquinoline-2,9(1*H*,3*H*,8*H*,10*H*)-diyl)bis(3-(1*H*-indol-3-yl)propanoic acid) (3e)

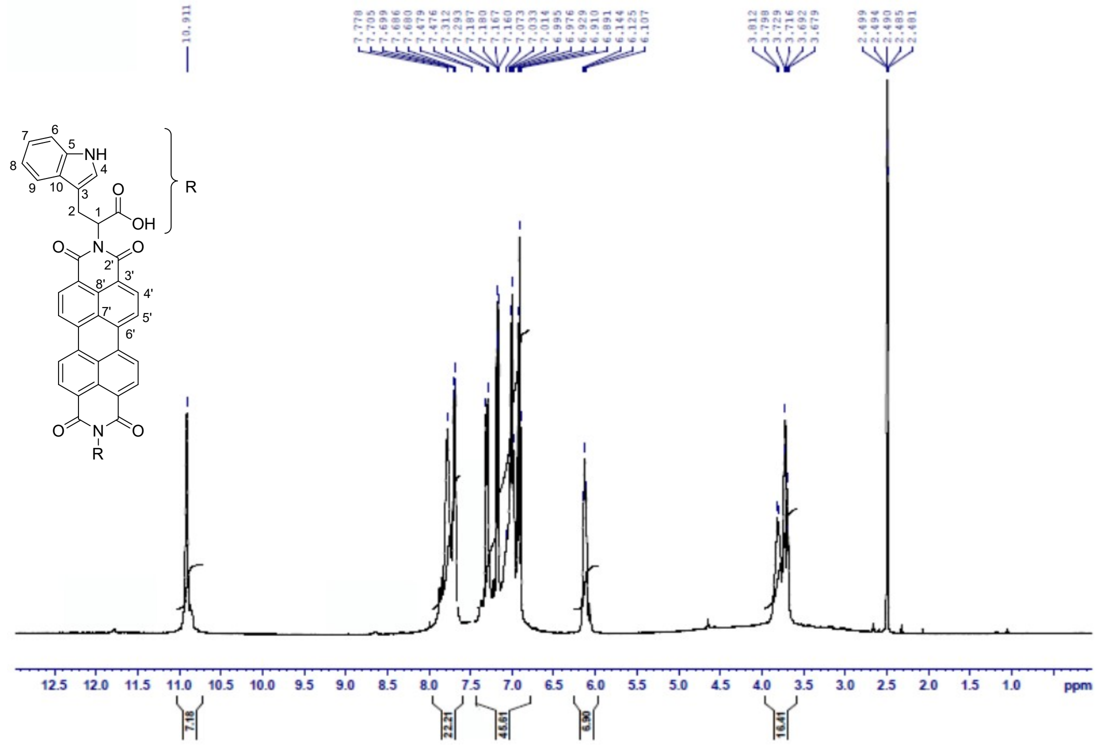

Figure 12. ^{1}H NMR spectrum of **3e**.

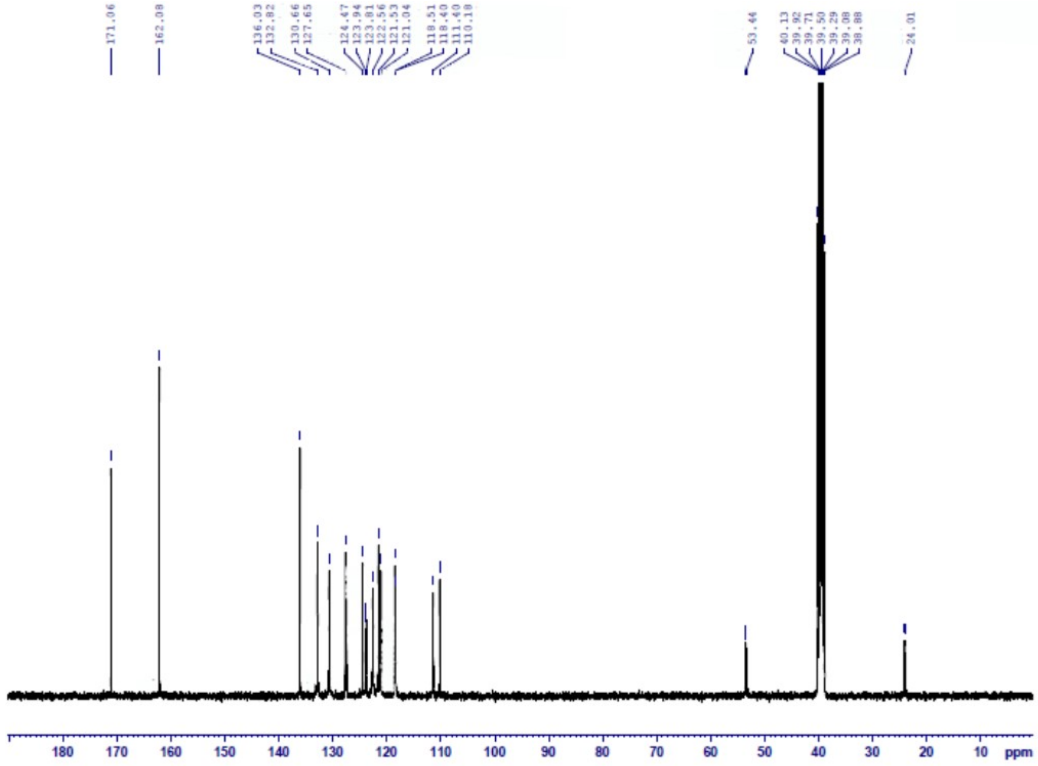

Figure 13. ^{13}C NMR spectrum of **3e**.

2,2'-(1,3,8,10-tetraoxoanthra[2,1,9-def:6,5,10-d'e'f']diisoquinoline-2,9(1*H*,3*H*,8*H*,10*H*)-diyl)disuccinic acid (3f)

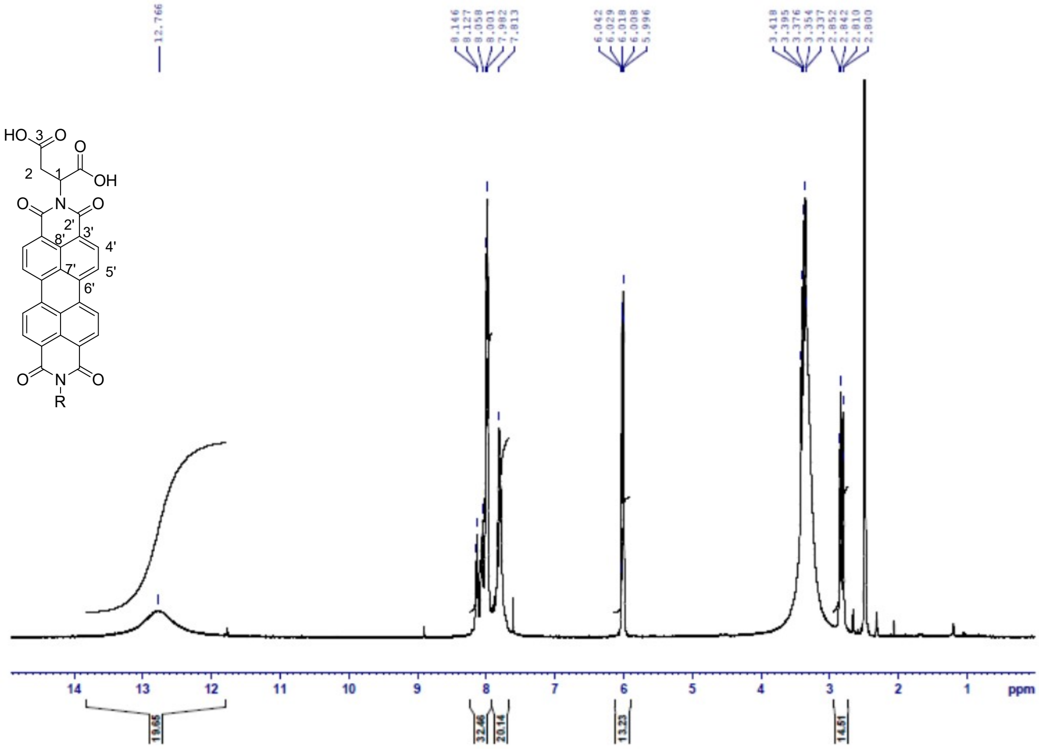

Figure 14. ^{1}H NMR spectrum of **3f**.

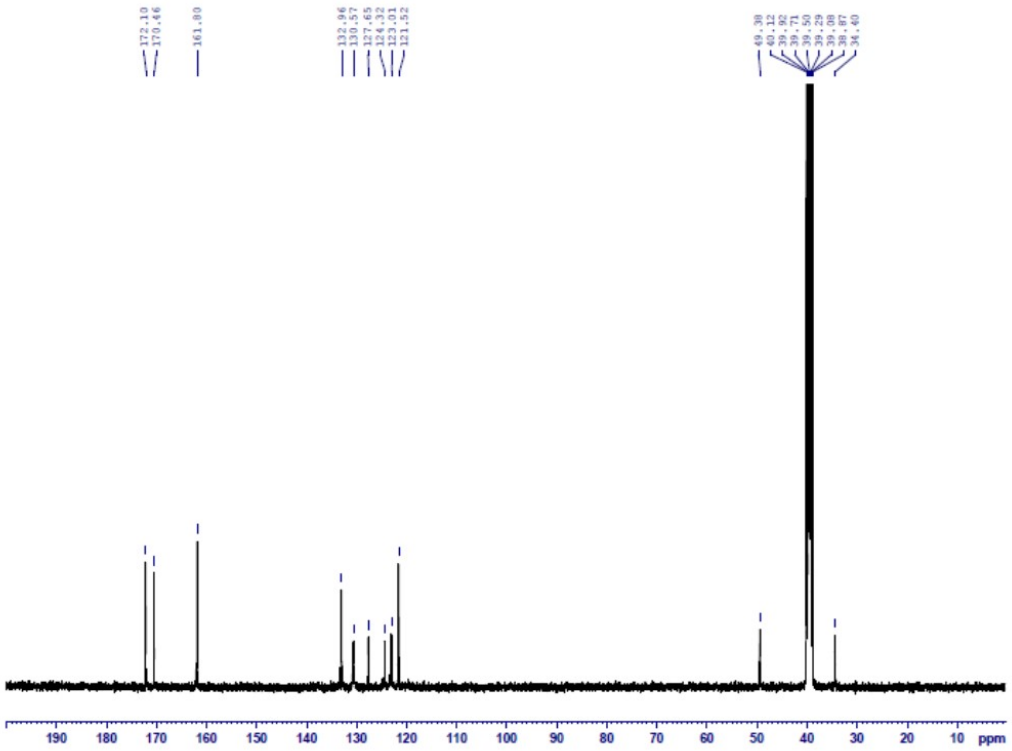

Figure 15. ^{13}C NMR spectrum of **3f**.

2,2'-(1,3,8,10-tetraoxoanthra[2,1,9-def:6,5,10-d'e'f']diisoquinoline-2,9(1*H*,3*H*,8*H*,10*H*)-diyl)bis(3-hydroxypropanoic acid) (3g)

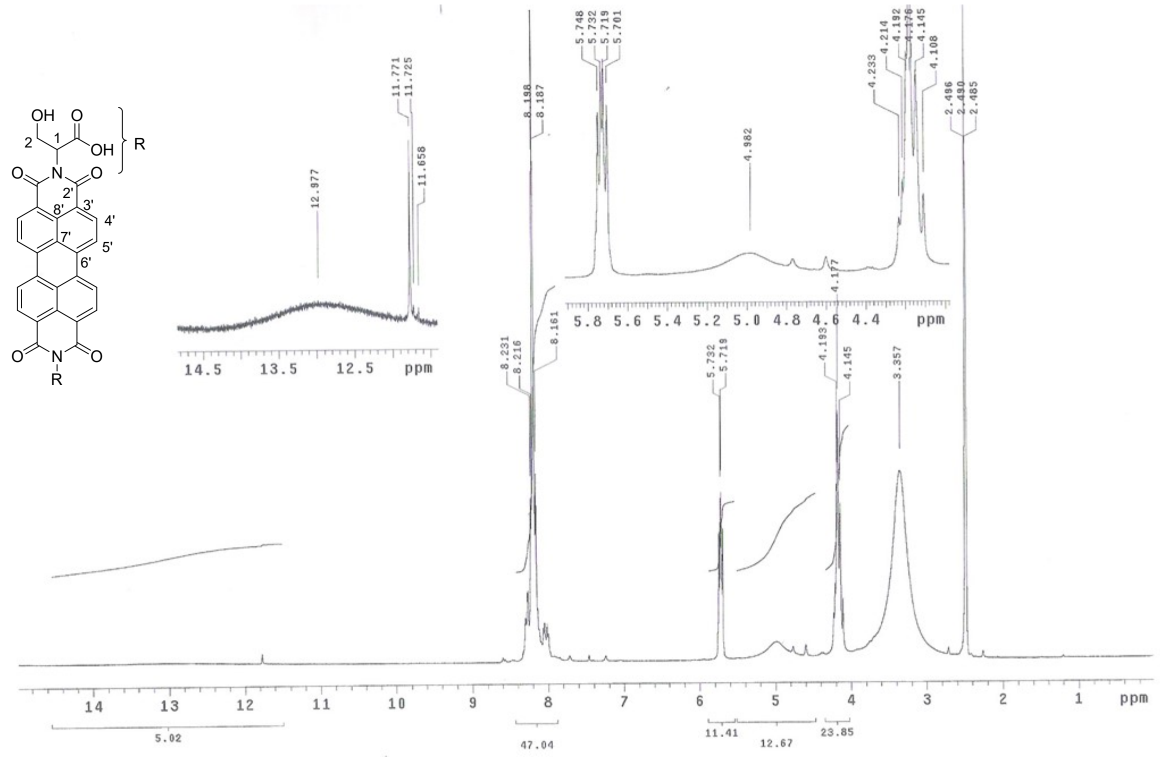

Figure 16. ^{1}H NMR spectrum of **3g**.

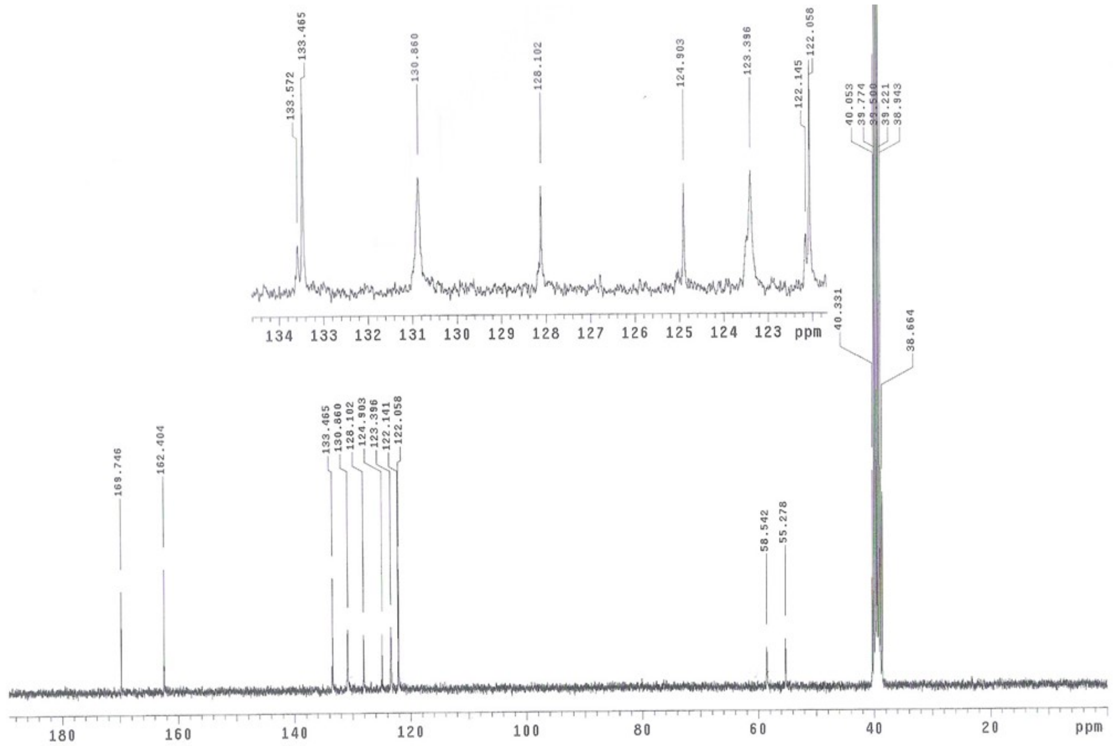

Figure 17. ^{13}C NMR spectrum of **3g**.

2,9-bis(2-aminoethyl)anthra[2,1,9-def:6,5,10-d'e'f']diisoquinoline-1,3,8,10(2H,9H)-tetraone (3h)

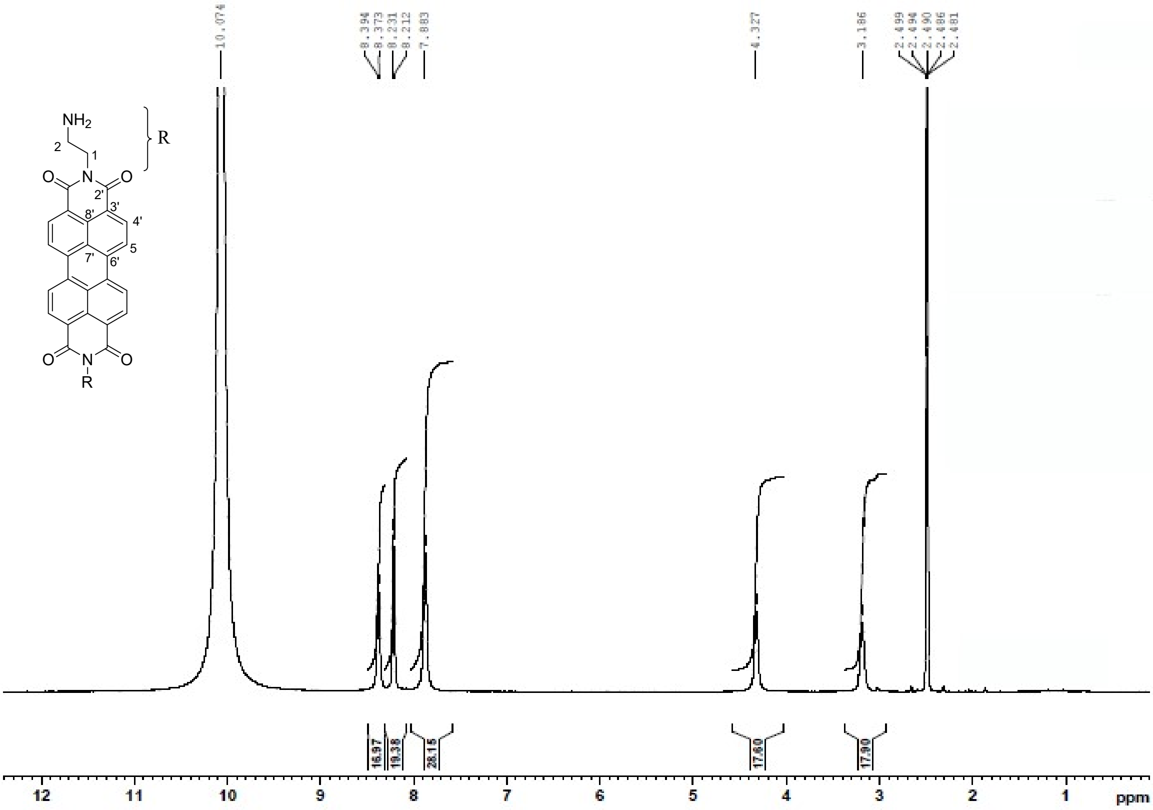

Figure 18. ^{1}H NMR spectrum of **3h**.

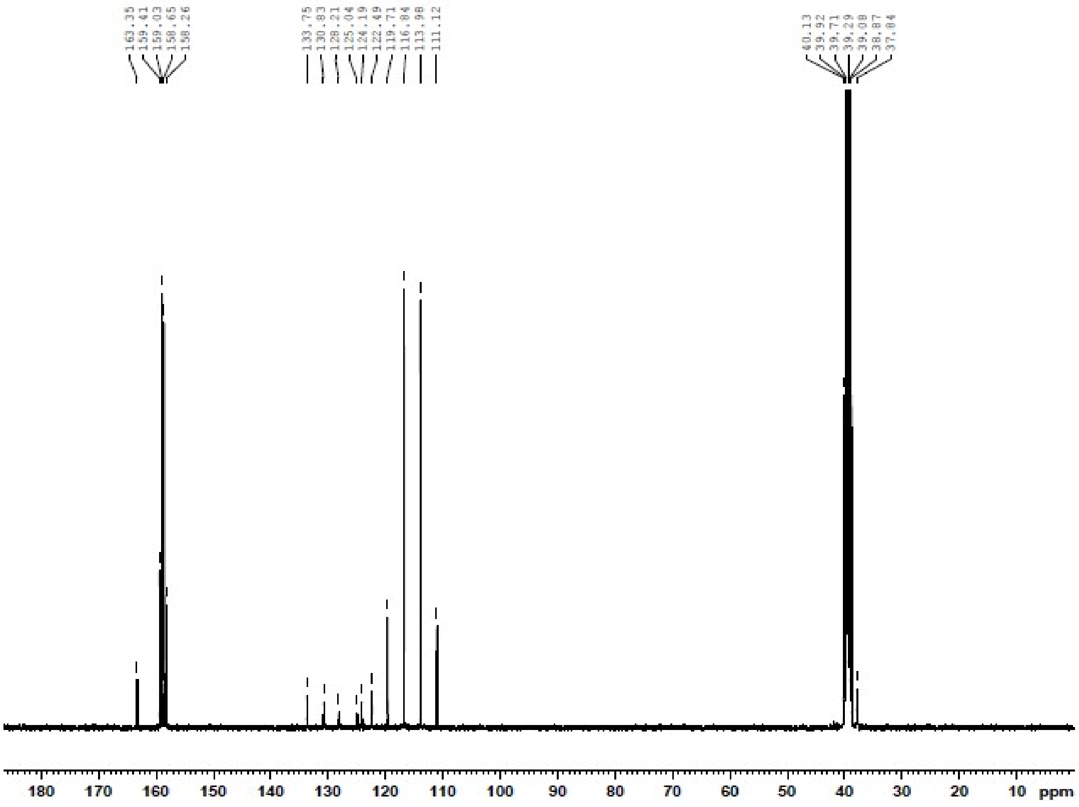

Figure 19. ^{13}C NMR spectrum of **3h**.

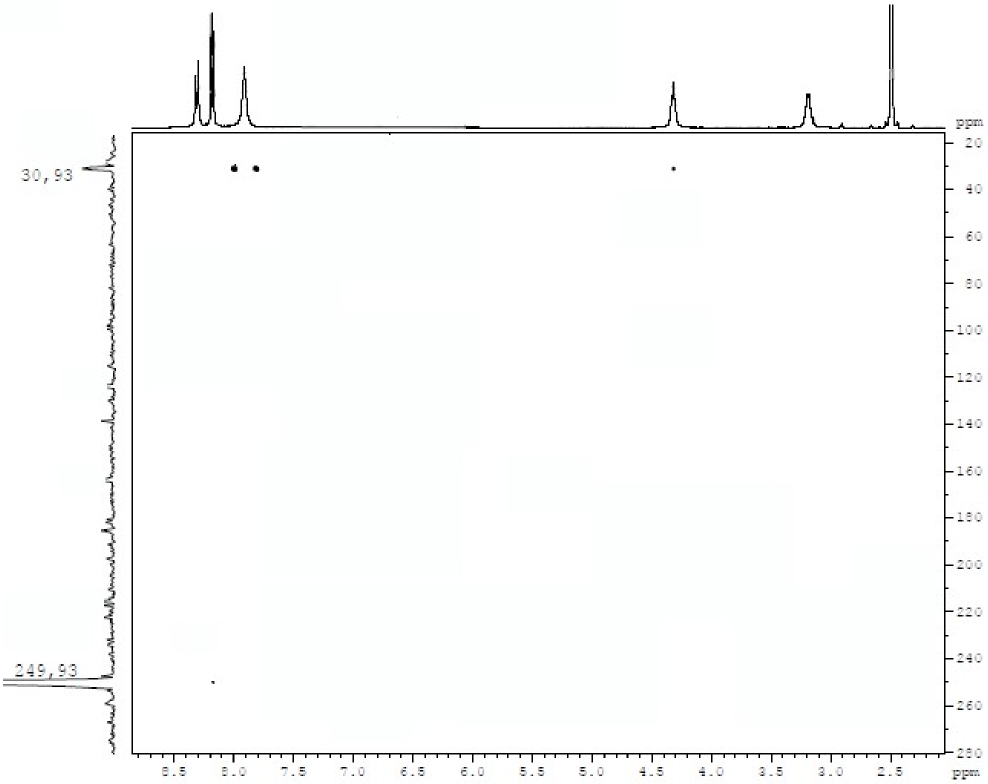

Figure 20. ^{15}N-^{1}H NMR correlation spectrum of **3h**.

2,9-bis(3-aminopropyl)anthra[2,1,9-def:6,5,10-d'e'f']diisoquinoline-1,3,8,10(2*H*,9*H*)-tetraone (3i)

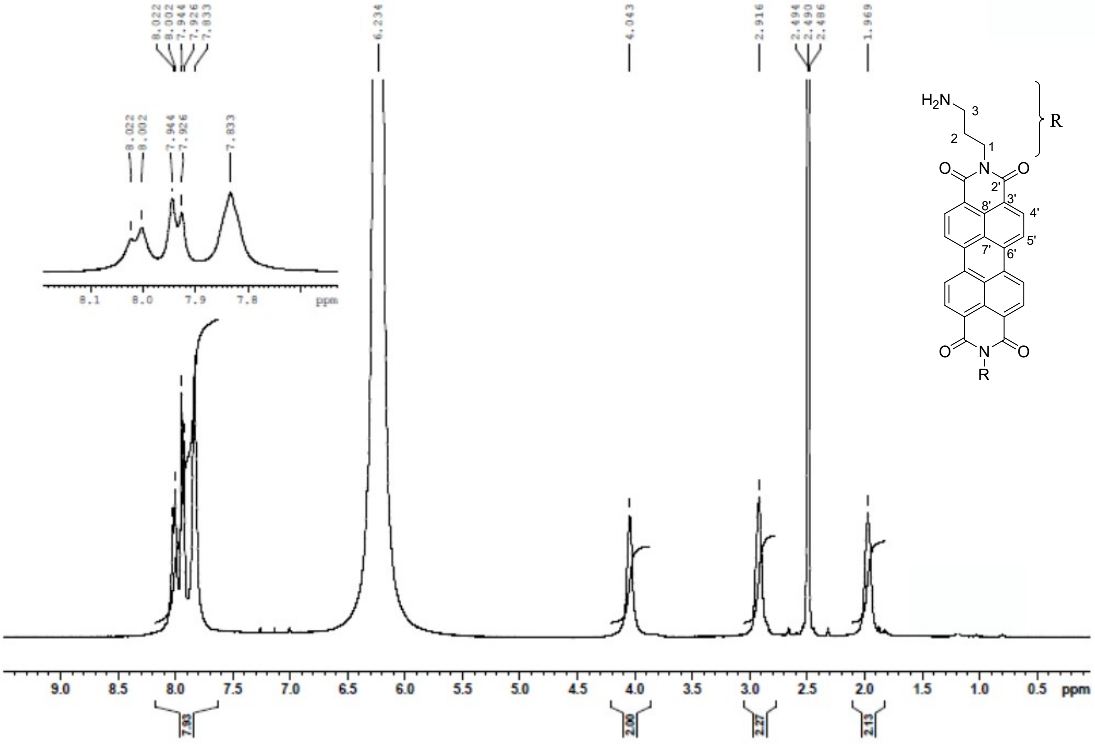

Figure 21. ^{1}H NMR spectrum of **3i**.

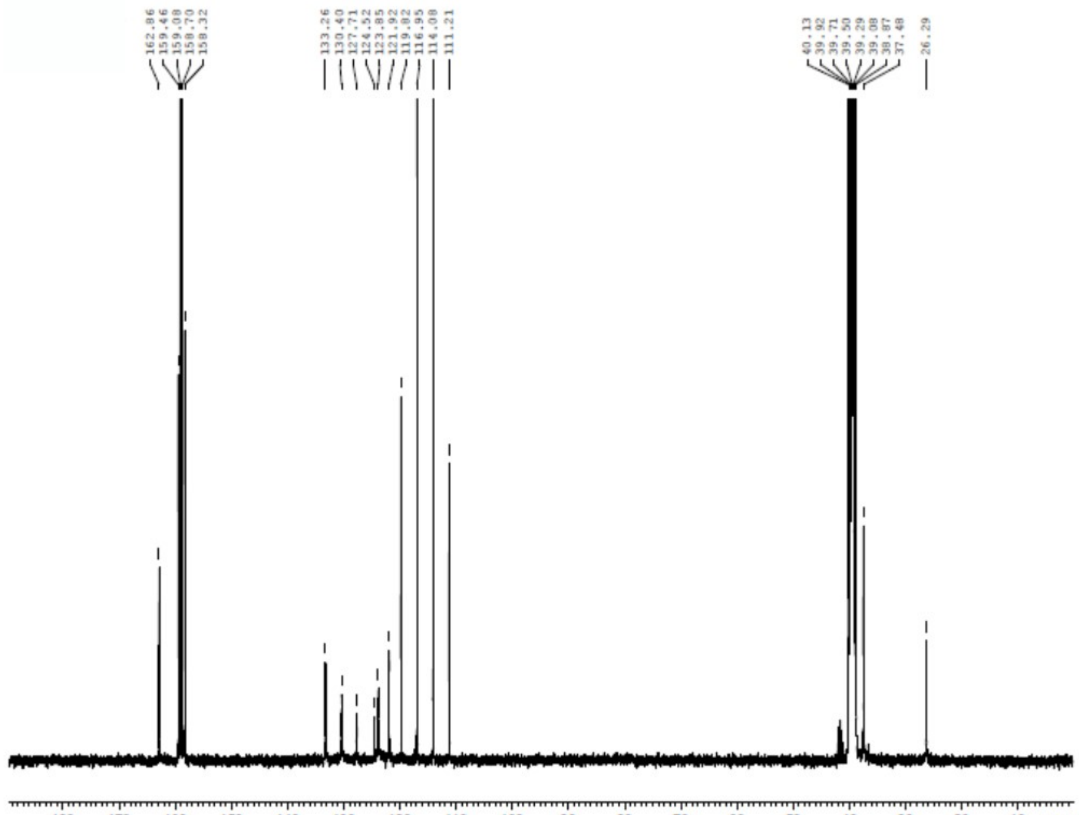

Figure 22. ^{13}C NMR spectrum of **3i**.

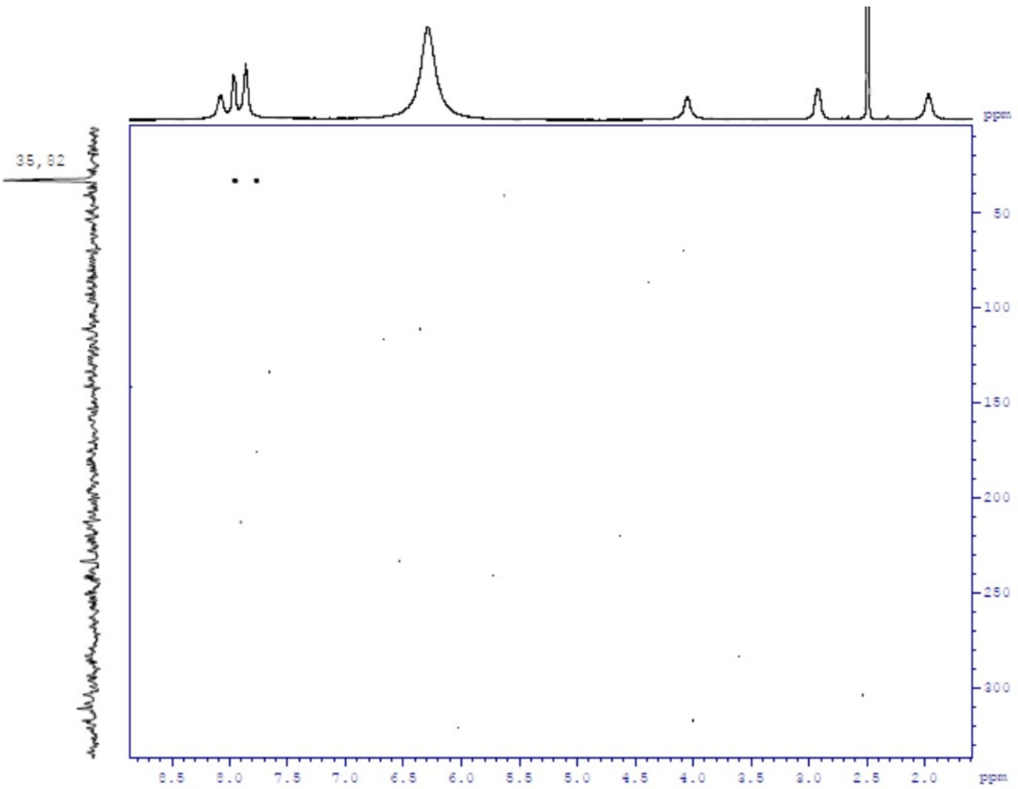

Figure 23. ^{15}N-^{1}H NMR correlation spectrum of **3i**.

2,9-bis(4-aminophenyl)anthra[2,1,9-def:6,5,10-d'e'f']diisoquinoline-1,3,8,10(2*H*,9*H*)-tetraone (3j)

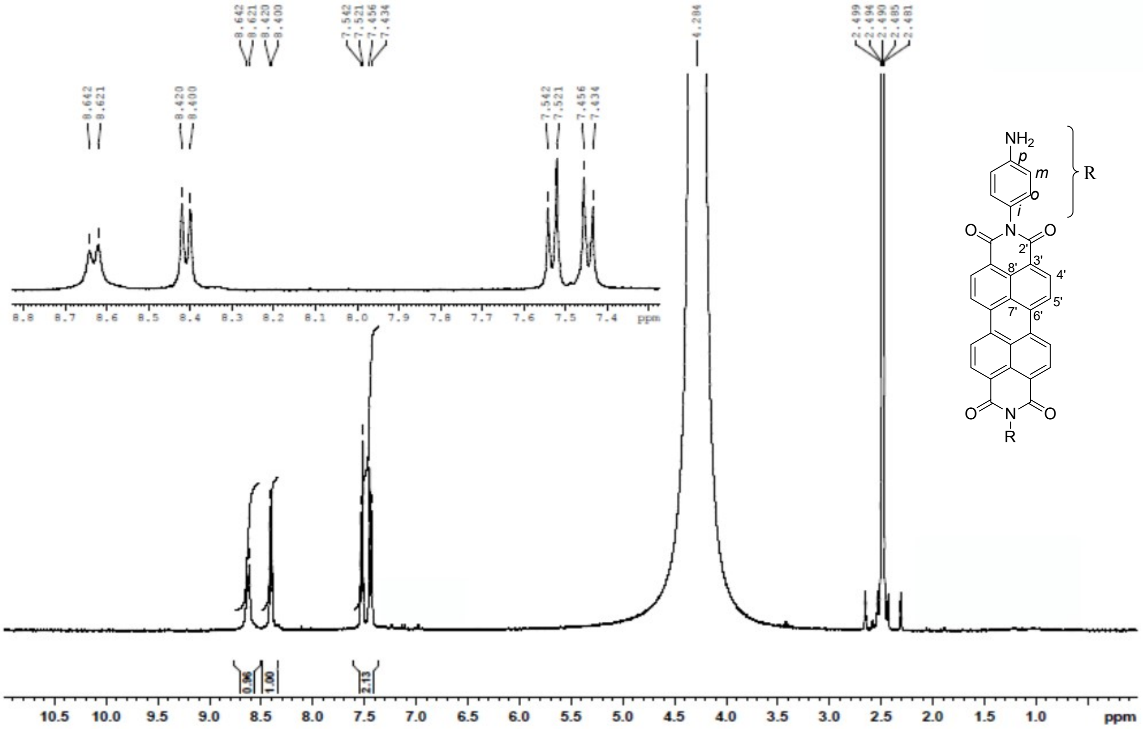

Figure 24. ^{1}H NMR spectrum of **3j**.

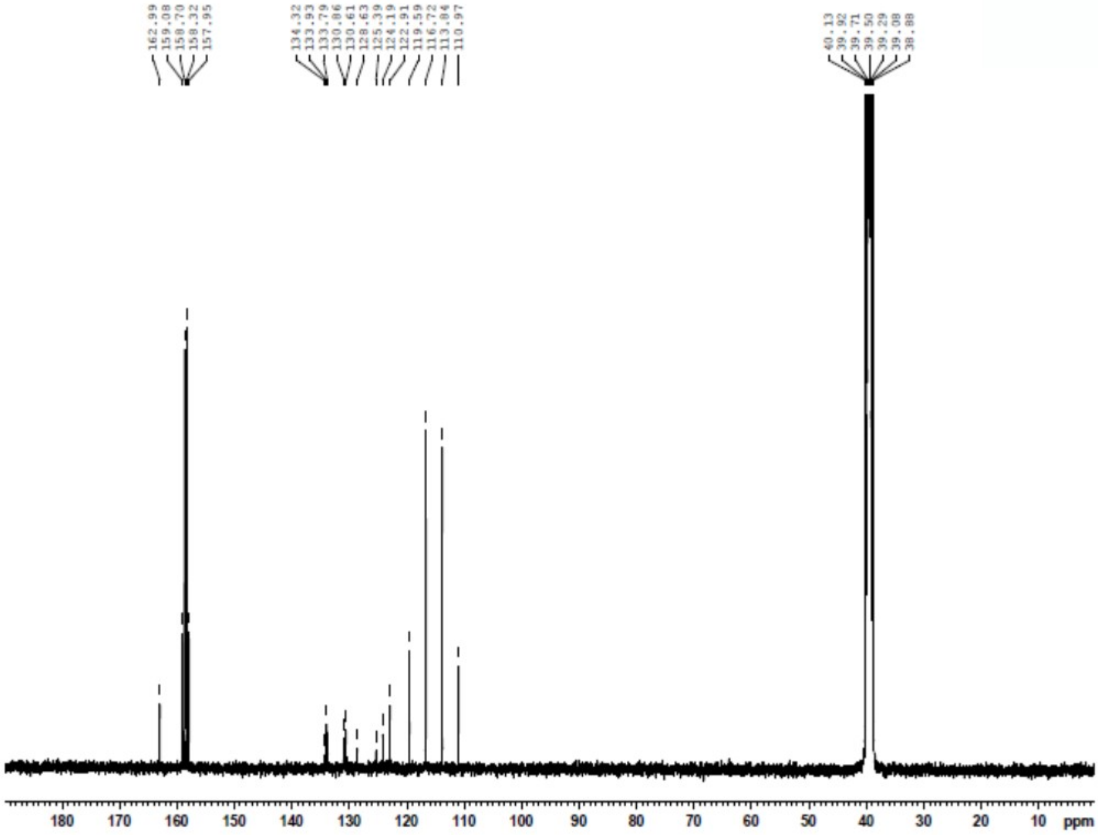

Figure 25. ^{13}C NMR spectrum of **3j**.

2,9-bis(3-hydroxypropyl)anthra[2,1,9-def:6,5,10-d'e'f']diisoquinoline-1,3,8,10(2*H*,9*H*)-tetraone (3k)

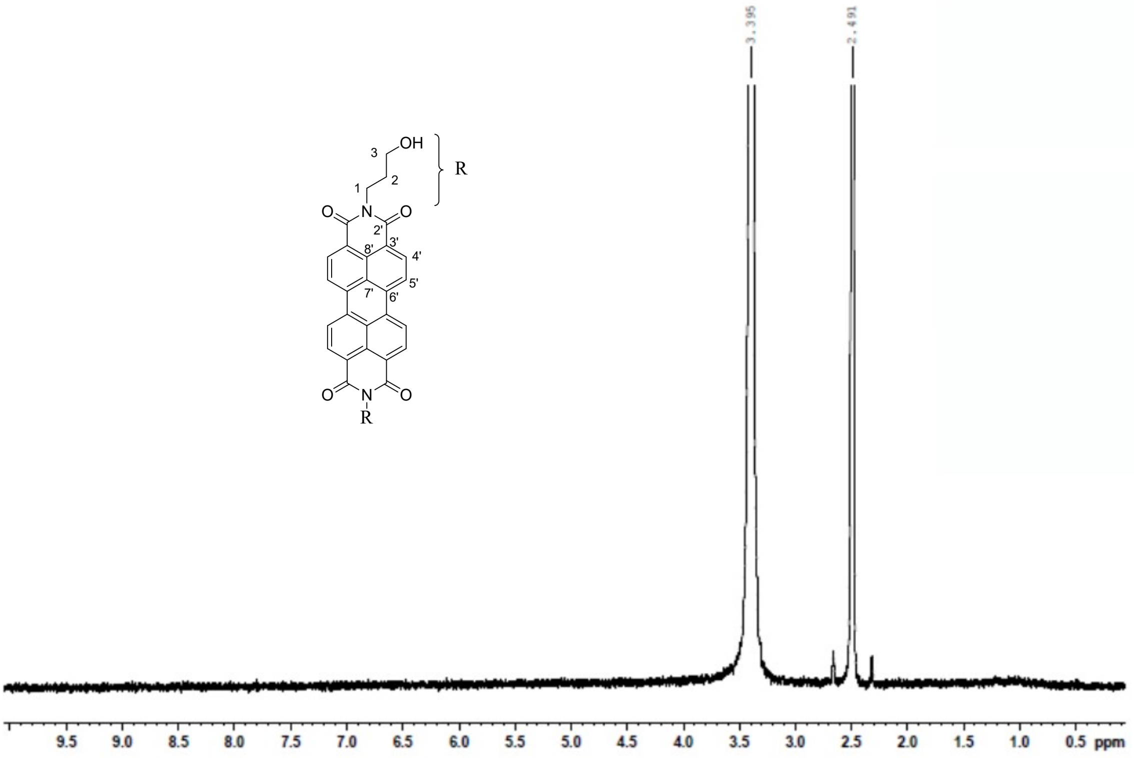

Figure 26. ¹H NMR spectrum of **3k**.

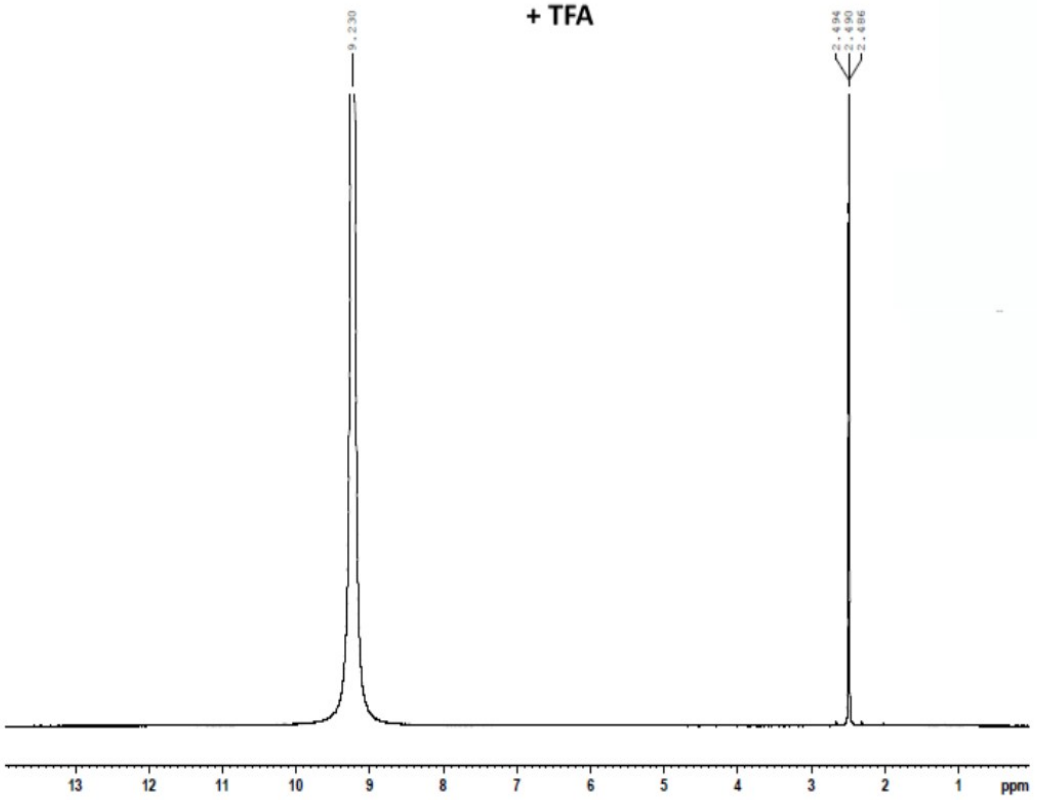

Figure 27. ¹H NMR spectrum of **3k** + TFA.

2,9-bis(1-hydroxybutan-2-yl)anthra[2,1,9-def:6,5,10-d'e'f']diisoquinoline-1,3,8,10(2*H*,9*H*)-tetraone (3l)

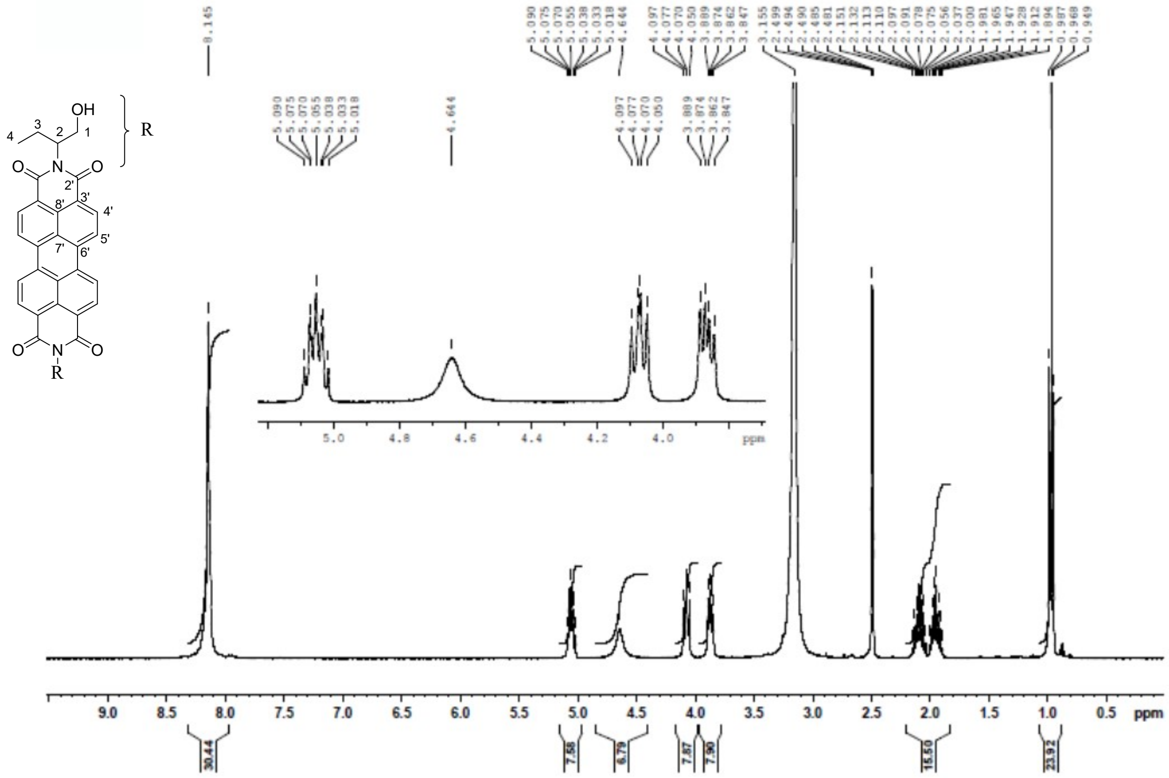

Figure 28. ^{1}H NMR spectrum of **3l**.

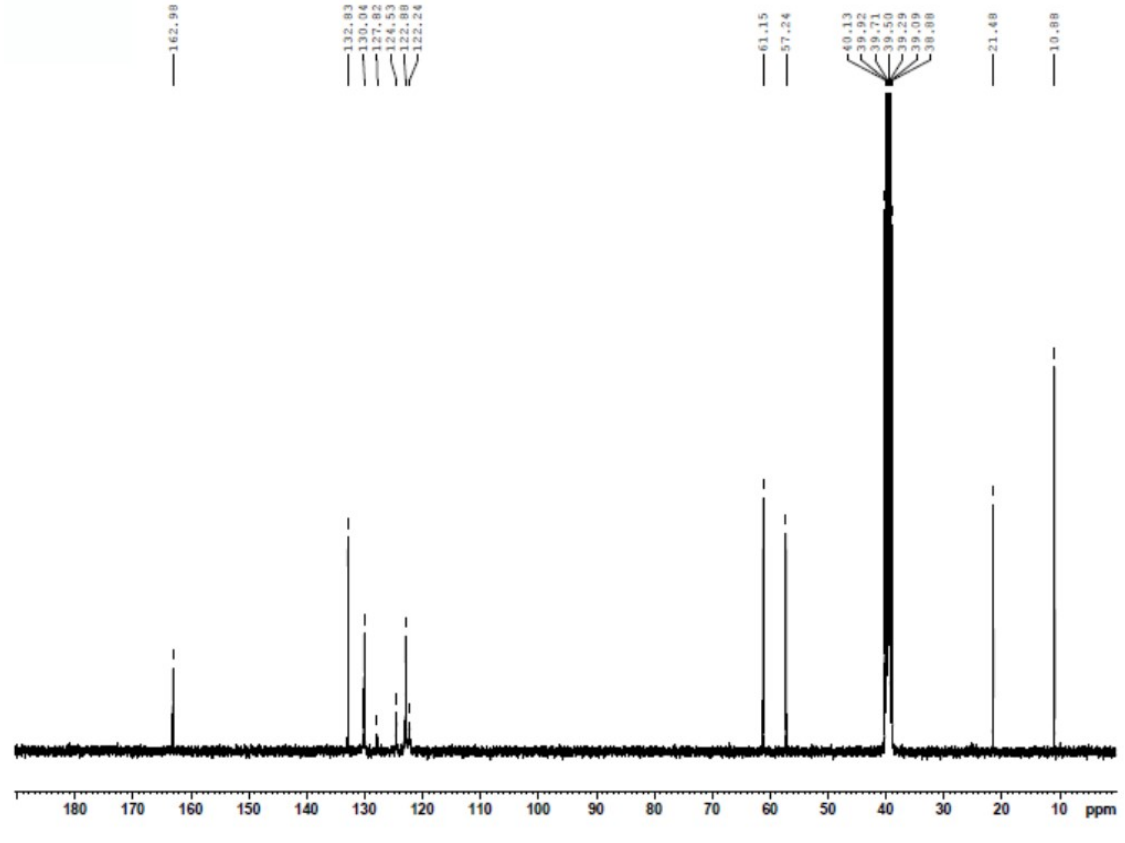

Figure 29. ^{13}C NMR spectrum of **3l**.

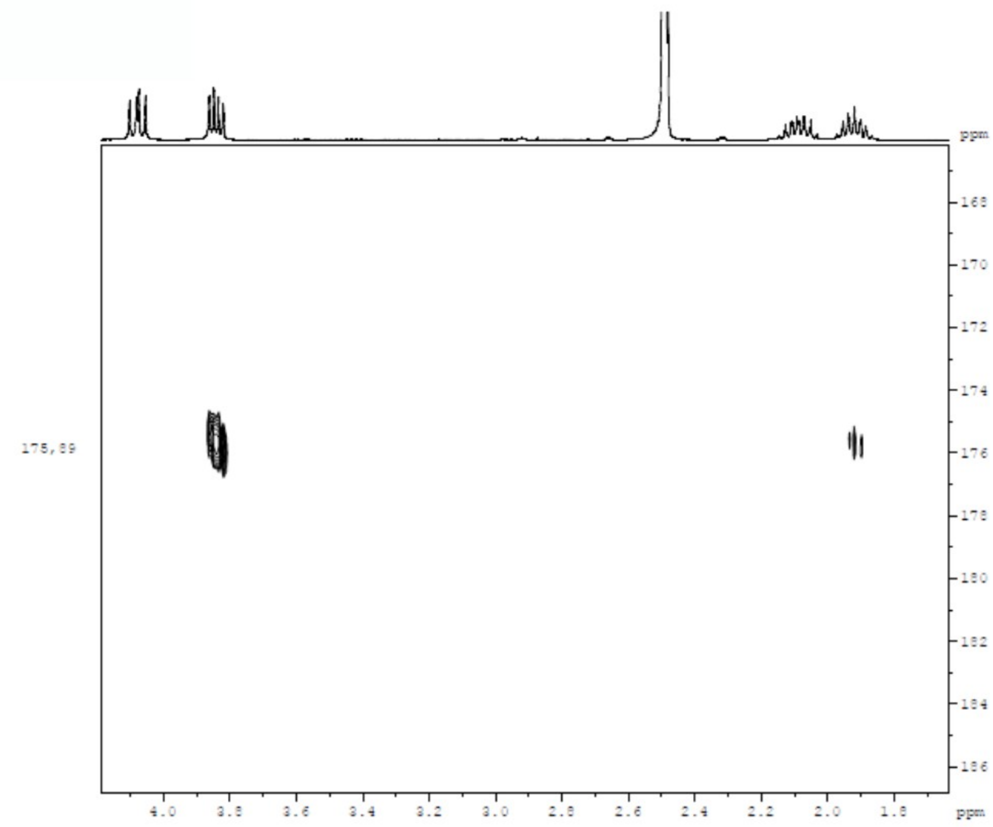

Figure 30. ^{15}N-^{1}H NMR correlation spectrum of **3l**.

2,9-bis(phenylamino)anthra[2,1,9-def:6,5,10-d'e'f']diisoquinoline-1,3,8,10(2*H*,9*H*)-tetraone (3m)

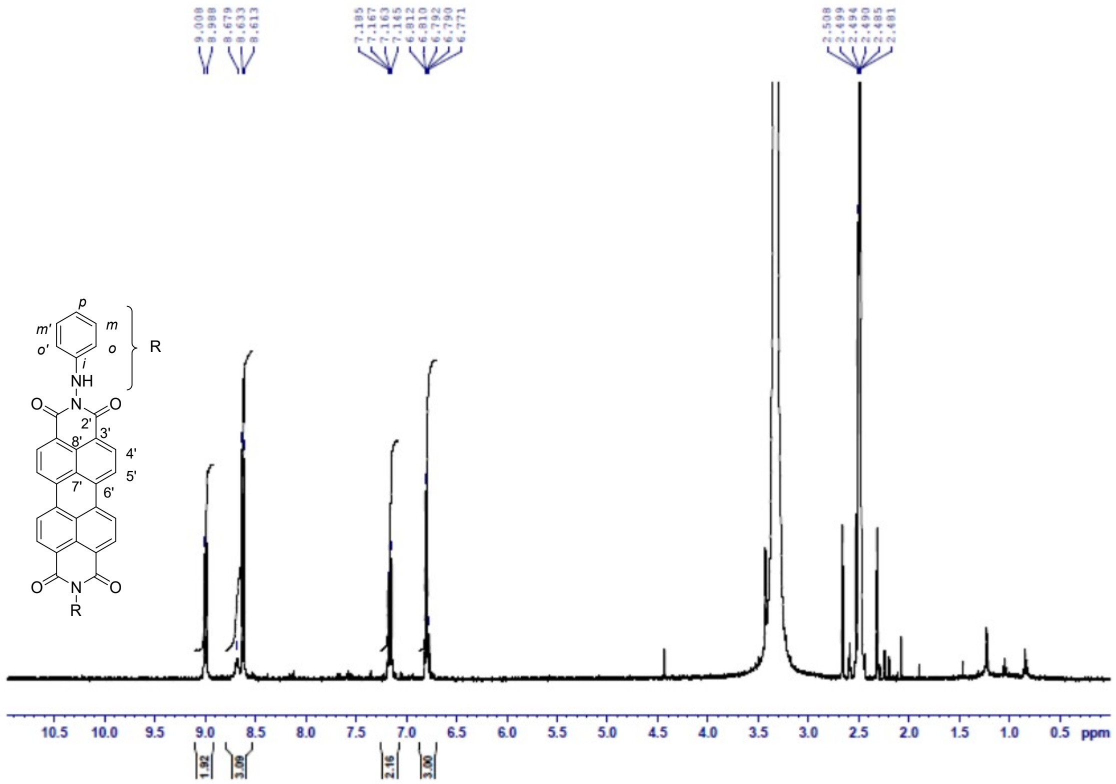

Figure 31. ^{1}H NMR spectrum of **3m**.

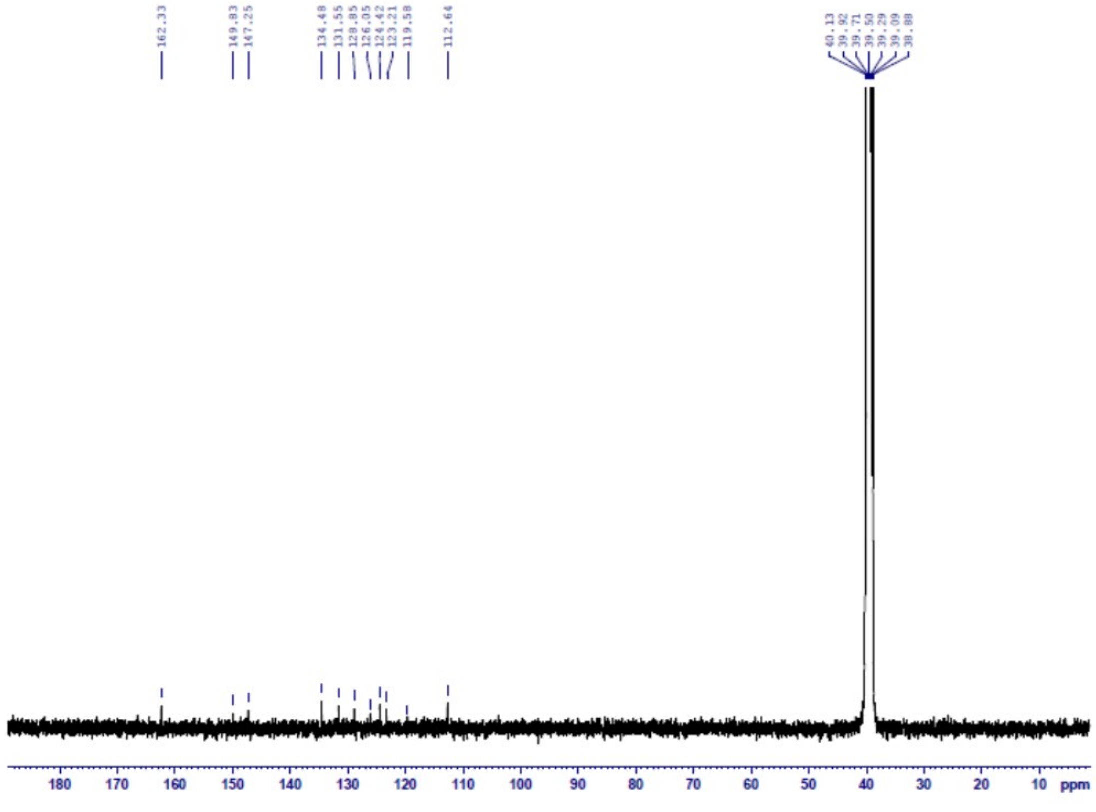

Figure 32. ^{13}C NMR spectrum of **3m**.

2,2'-(1,3,8,10-tetraoxoanthra[2,1,9-def:6,5,10-d'e'f']diisoquinoline-2,9(1*H*,3*H*,8*H*,10*H*)-diyl)bis(3-(1*H*-imidazol-1-yl)propanoic acid) (4)

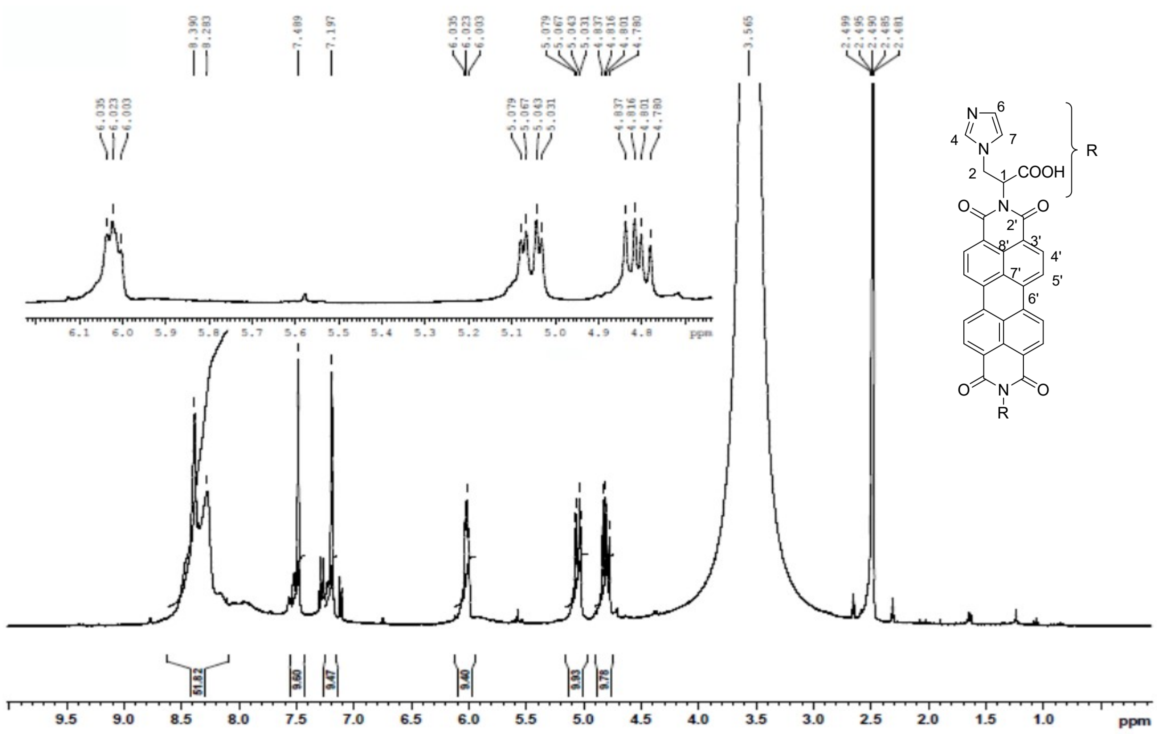

Figure 33. ^{1}H NMR spectrum of **4**.

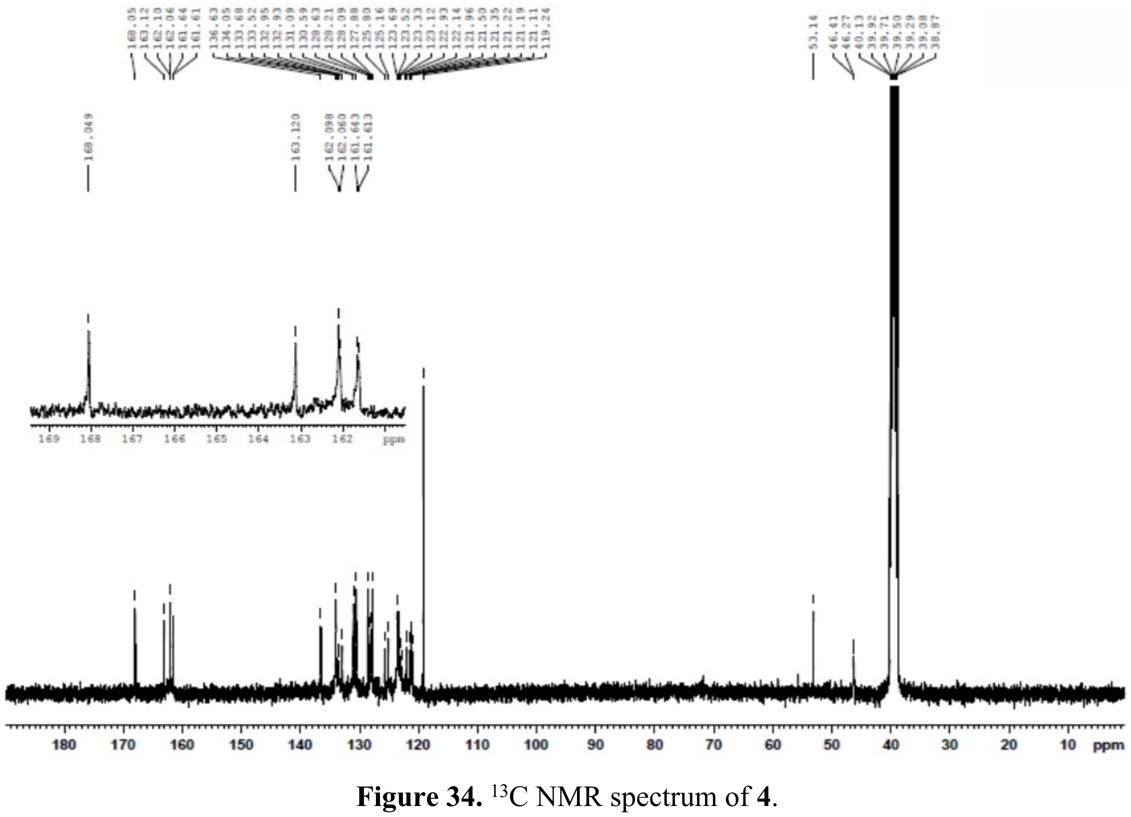

Figure 34. ^{13}C NMR spectrum of **4**.

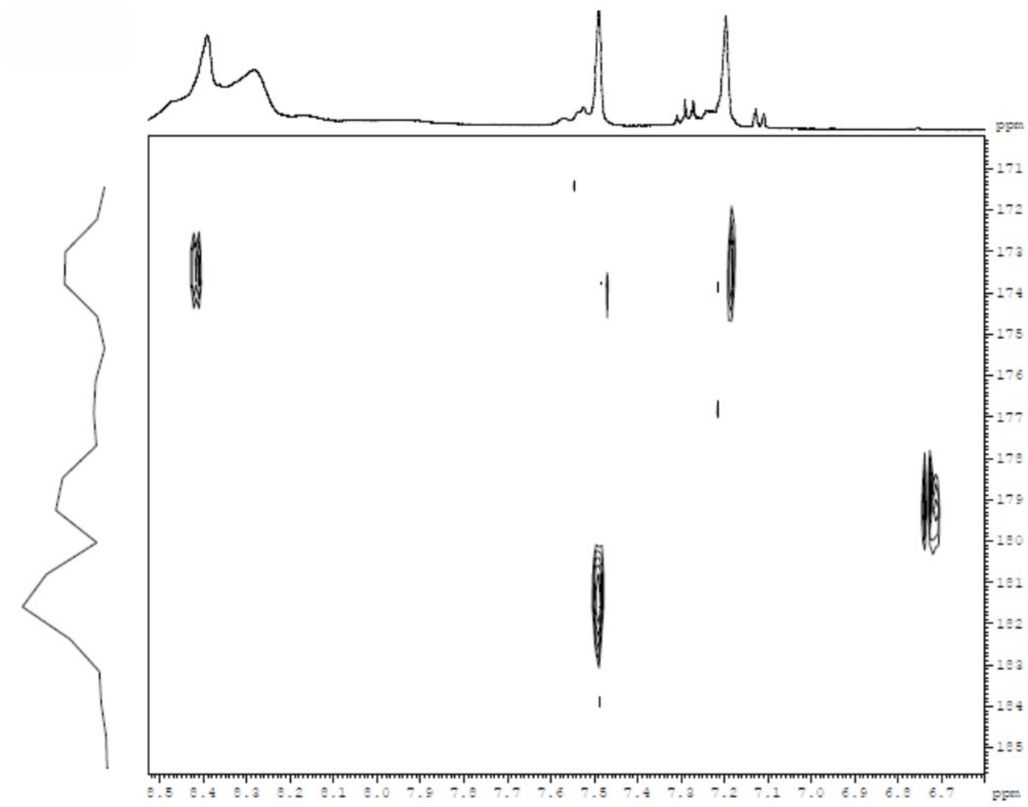

Figure 35. ^{15}N-^{1}H NMR correlation spectrum of **4**.

2,2'-(1,3,8,10-tetraoxoanthra[2,1,9-def:6,5,10-d'e'f']diisoquinoline-2,9(1*H*,3*H*,8*H*,10*H*)-diyl)bis(3-mercaptopropanoic acid) (9)

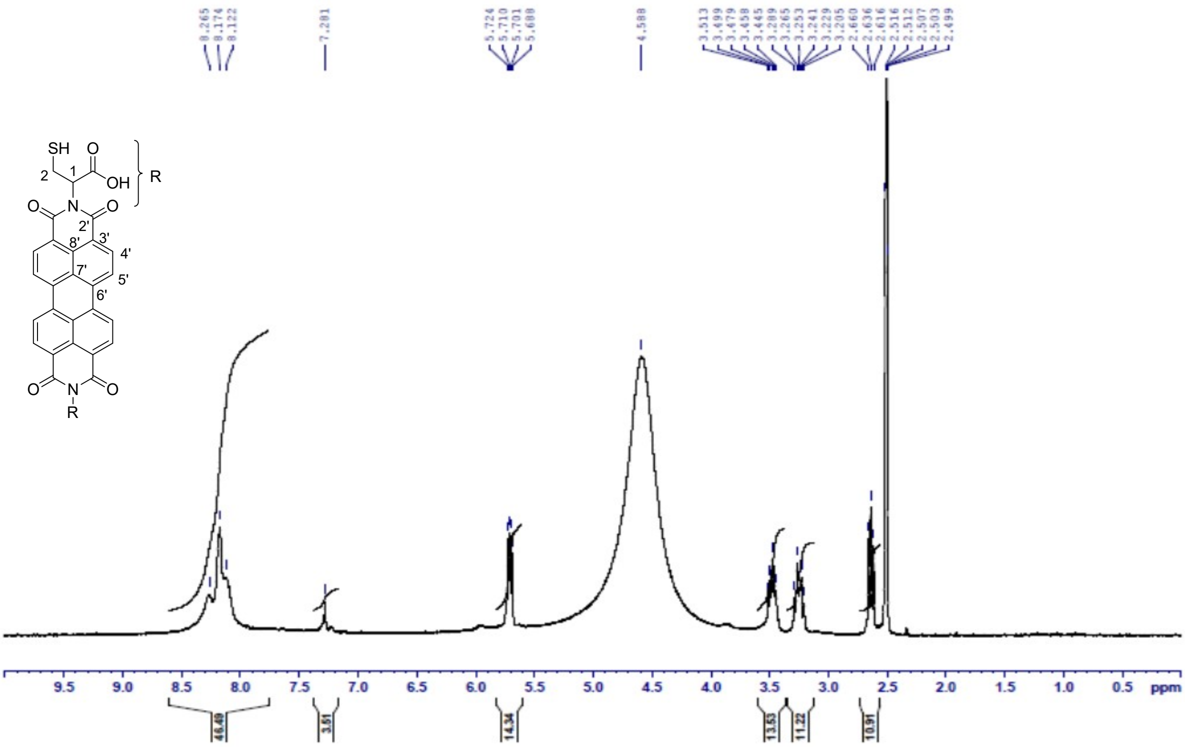

Figure 36. ^{1}H NMR spectrum of **9**.

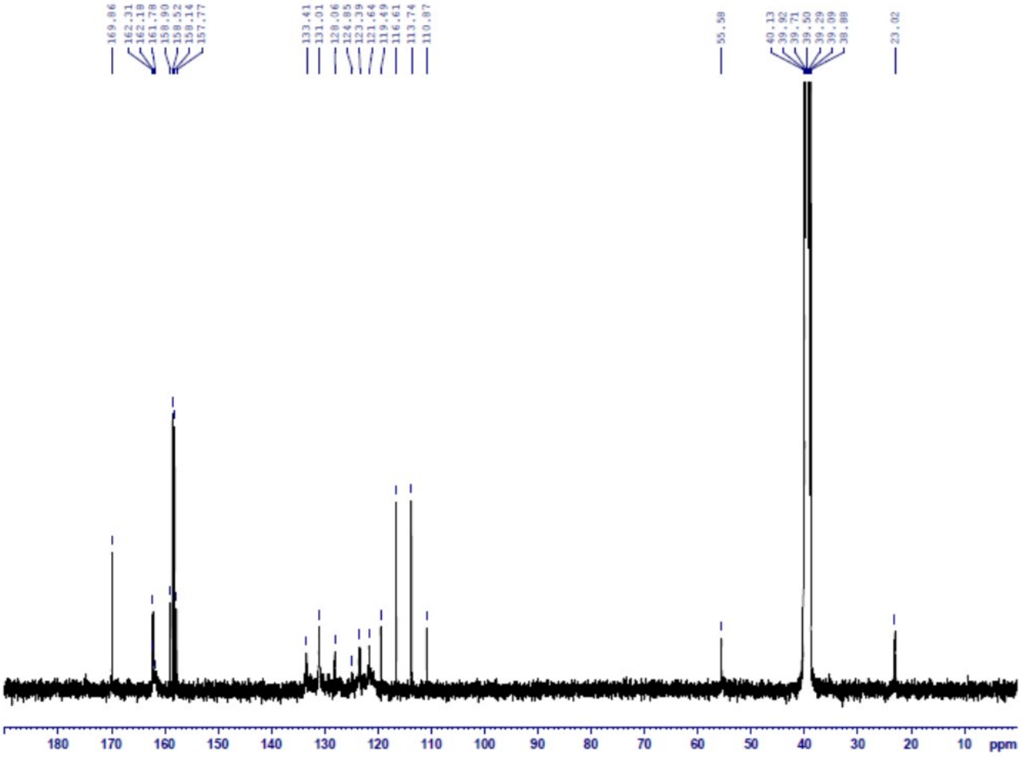

Figure 37. ^{13}C NMR spectrum of **9**.

6,6'-(1,3,8,10-tetraoxoanthra[2,1,9-def:6,5,10-d'e'f']diisoquinoline-2,9(1*H*,3*H*,8*H*,10*H*)-diyl)bis(2-aminohexanoic acid) (10)

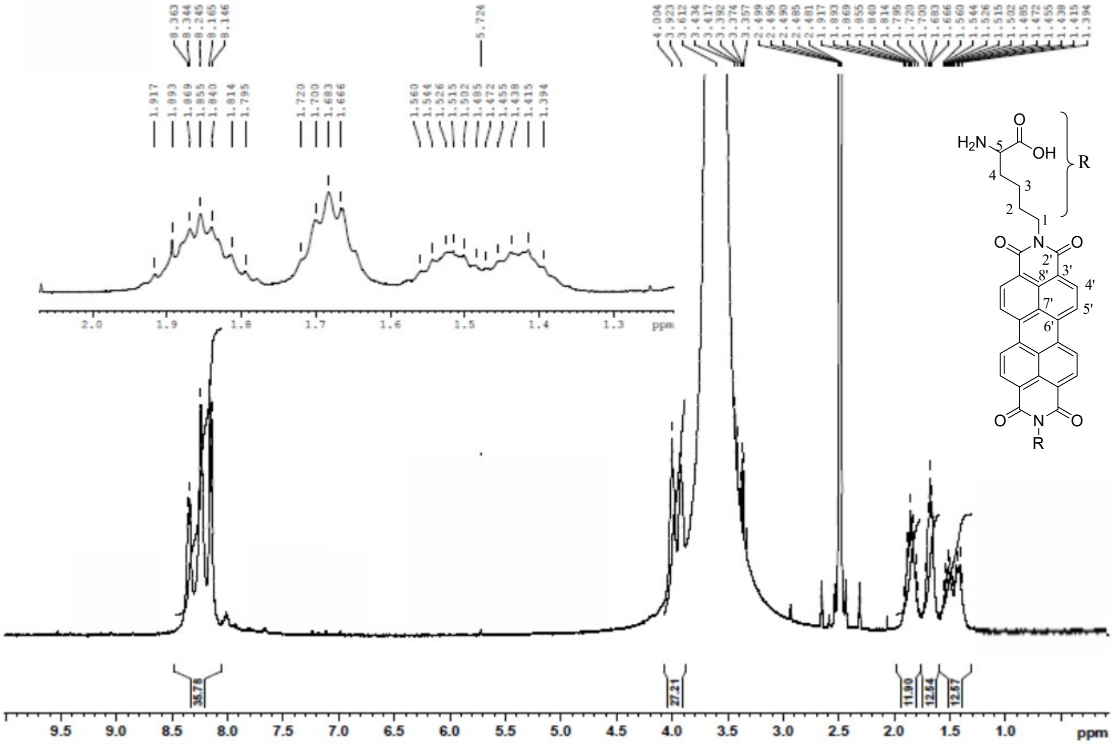

Figure 38. ^{1}H NMR spectrum of **10**.

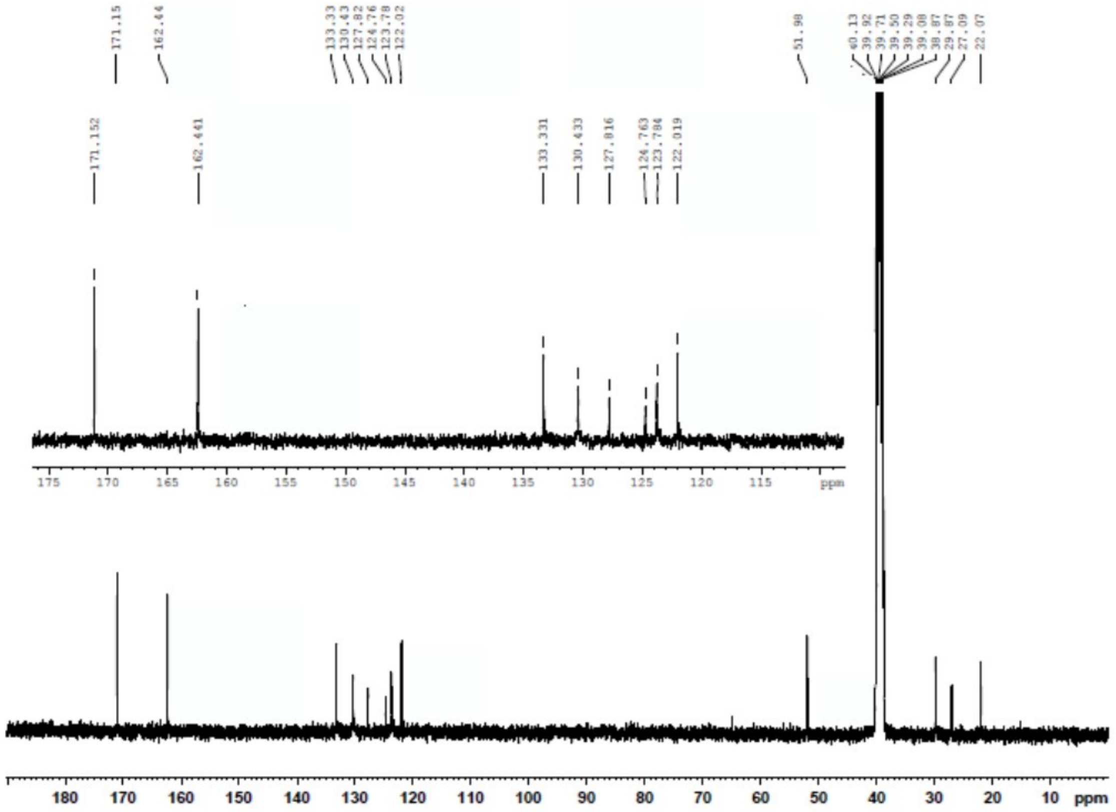

Figure 39. ^{13}C NMR spectrum of **10**.

(*Z*)-6,6'-(1,3,8,10-tetraoxoanthra[2,1,9-def:6,5,10-d'e'f']diisoquinoline-2,9(1*H*,3*H*,8*H*,10*H*)-diyl)bis(2-((*Z*)-3-carboxyacrylamido)hexanoic acid) (11)

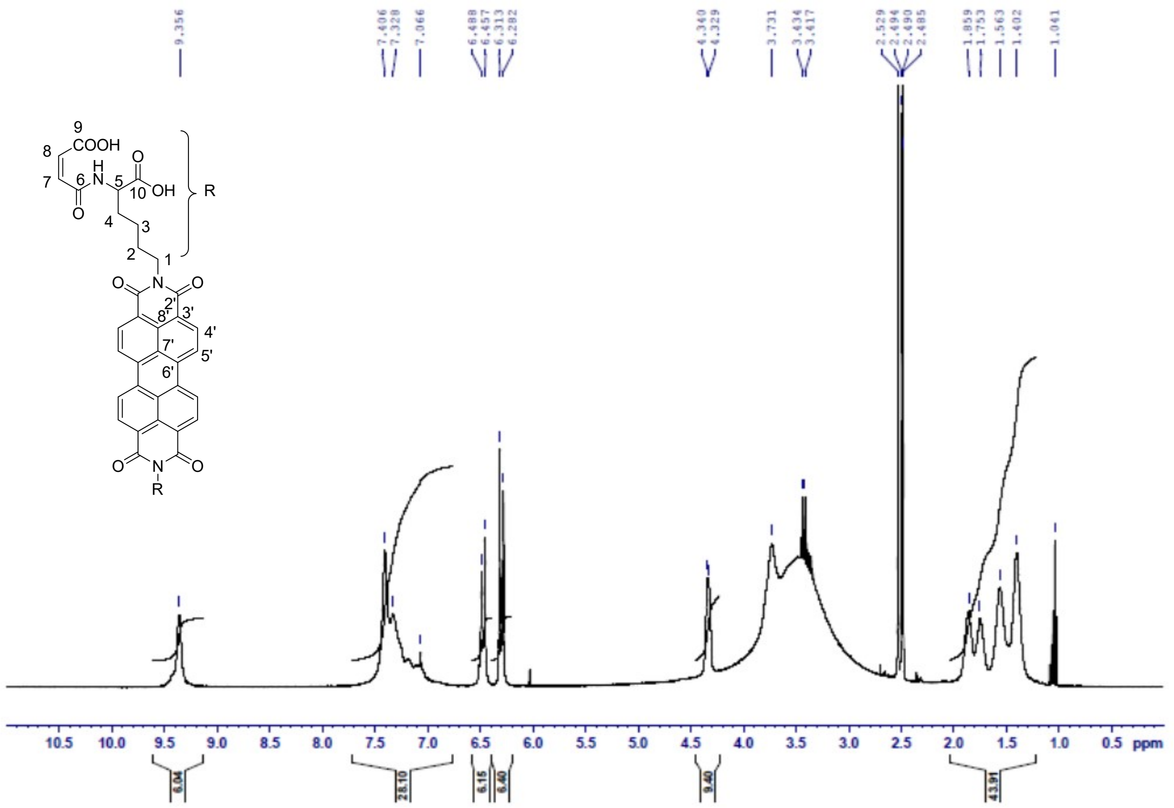

Figure 40. ^{1}H NMR spectrum of **11**.

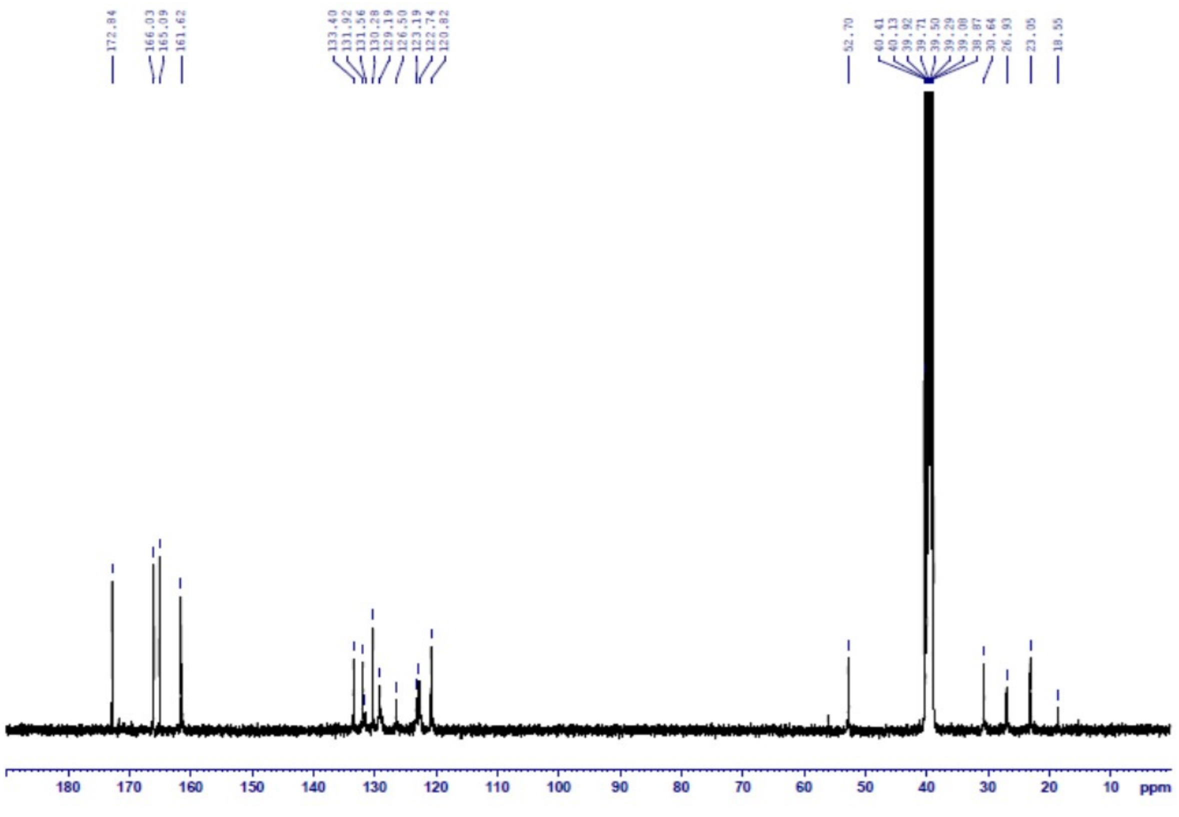

Figure 41. ^{13}C NMR spectrum of **11**.

6,6'-(1,3,8,10-tetraoxoanthra[2,1,9-def:6,5,10-d'e'f']diisoquinoline-2,9(1*H*,3*H*,8*H*,10*H*)-diyl)bis(2-(2,5-dioxo-2,5-dihydro-1*H*-pyrrol-1-yl)hexanoic acid) (12)

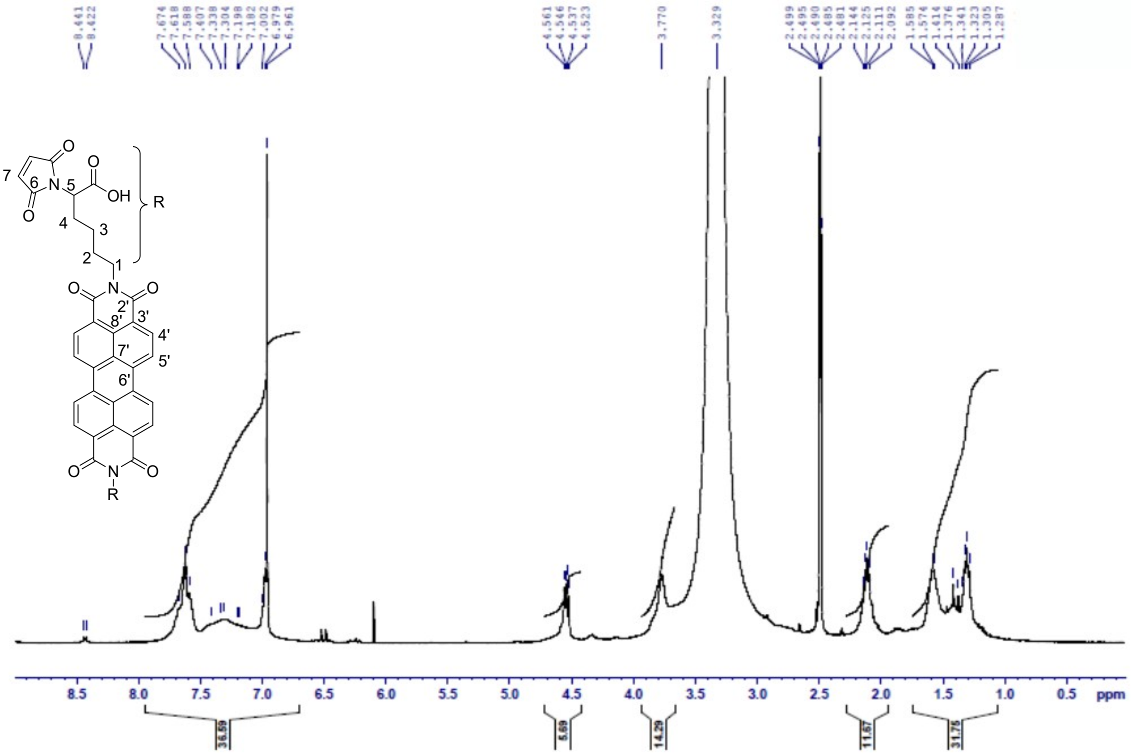

Figure 42. ^{1}H NMR spectrum of **12**.

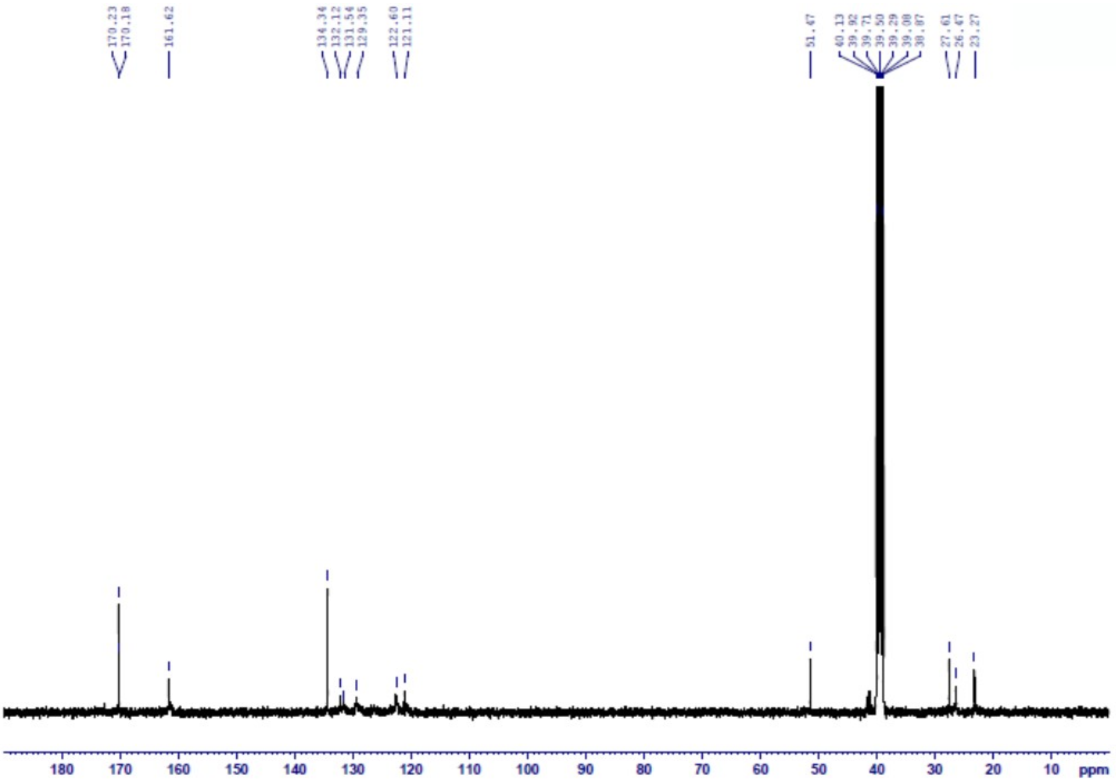

Figure 43. ^{13}C NMR spectrum of **12**.

4-((9,10-dioxo-9,10-dihydroanthracen-2-yl)amino)-4-oxobutanoic acid (14)

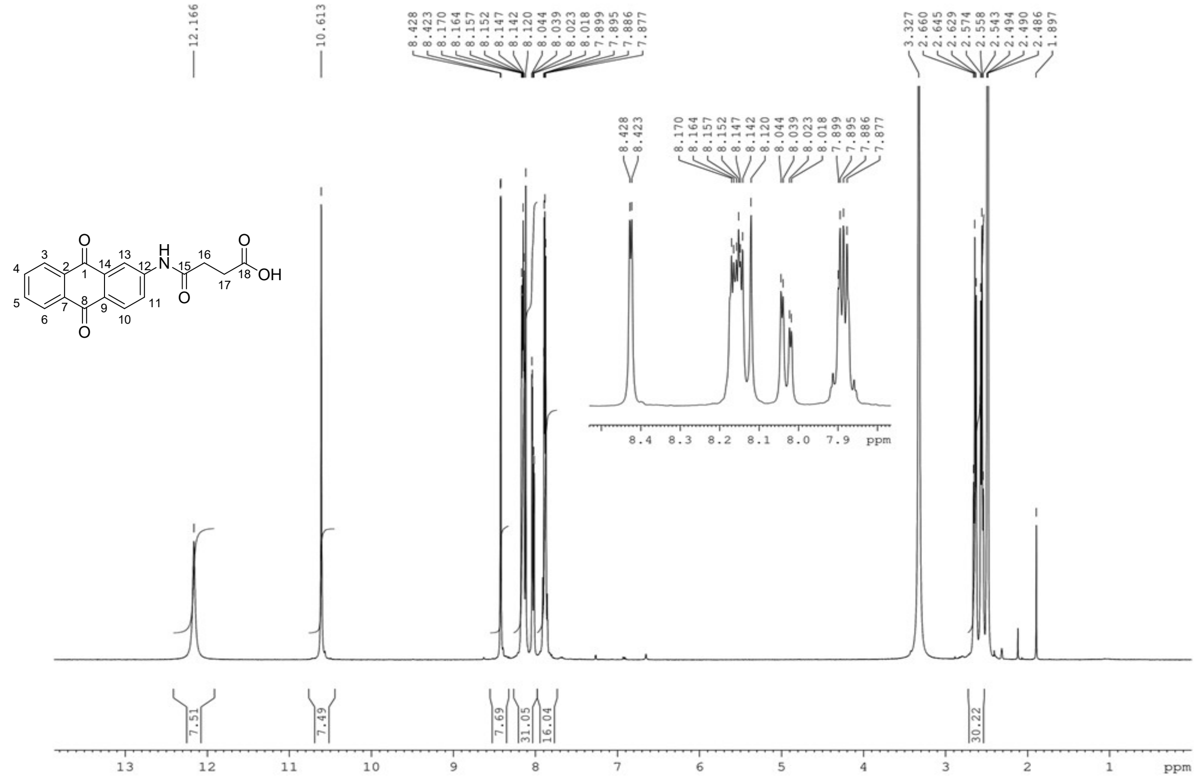

Figure 44. ^{1}H NMR spectrum of **14**.

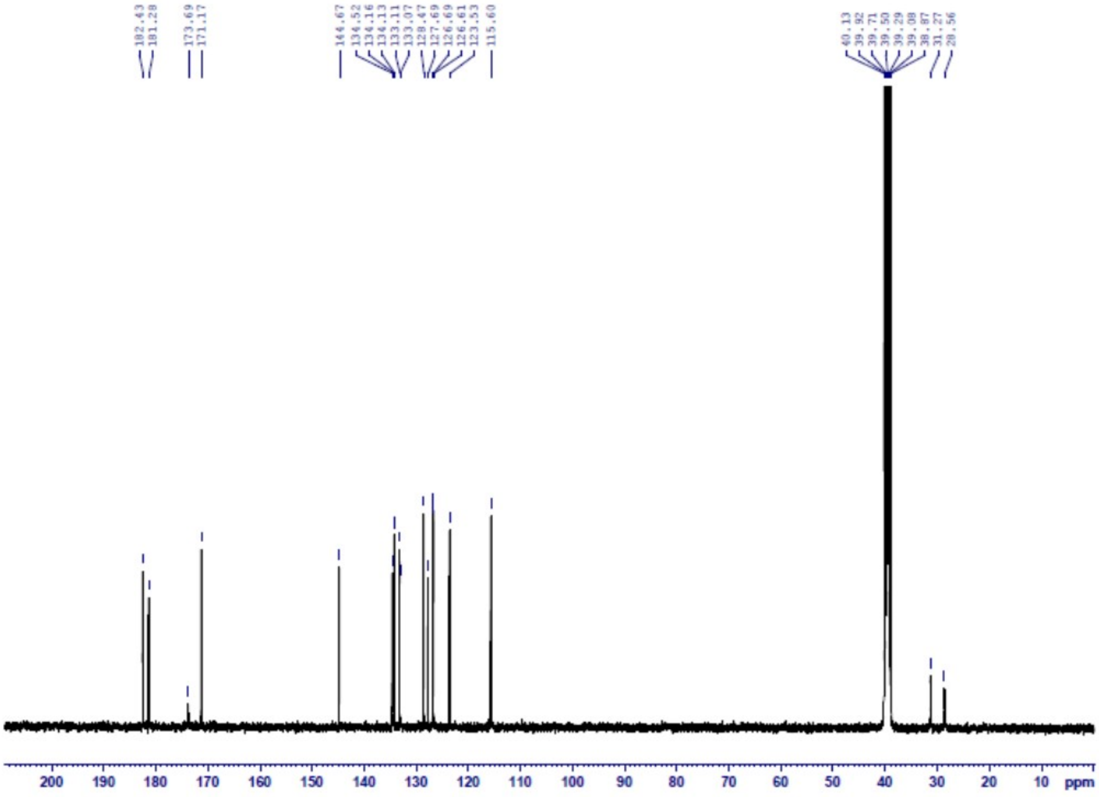

Figure 45. ^{13}C NMR spectrum of **14**.

1-(9,10-dioxo-9,10-dihydroanthracen-2-yl)pyrrolidine-2,5-dione (15)

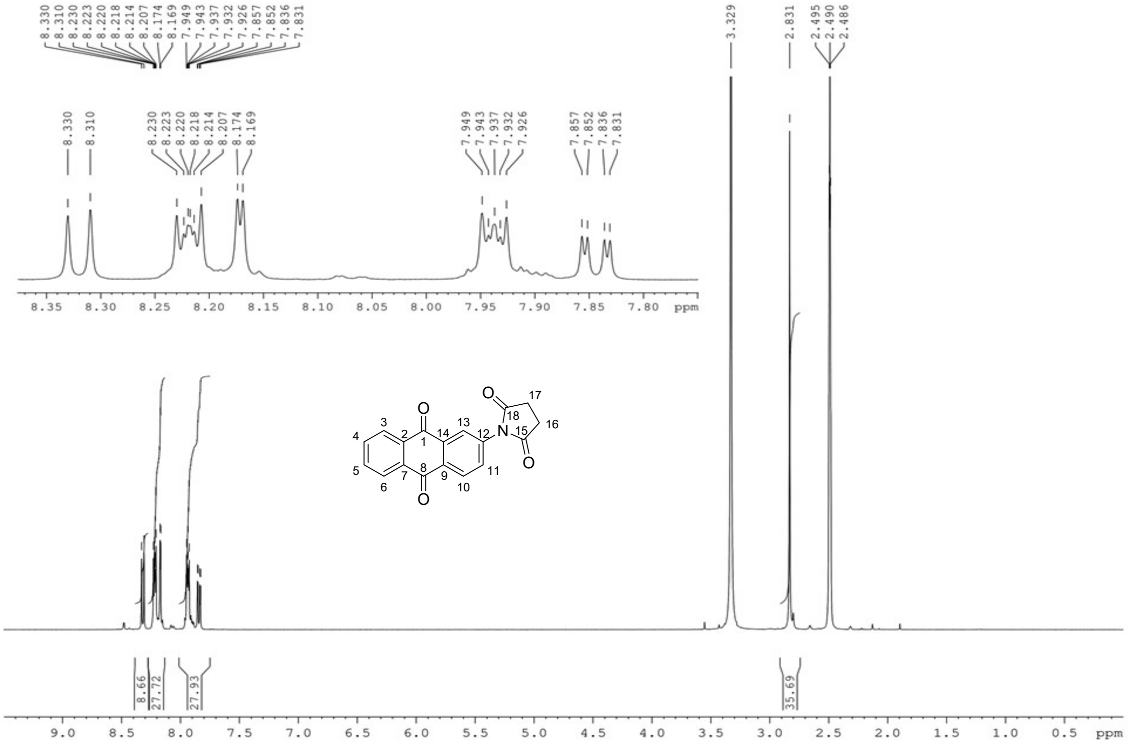

Figure 46. ^{1}H NMR spectrum of **15**.

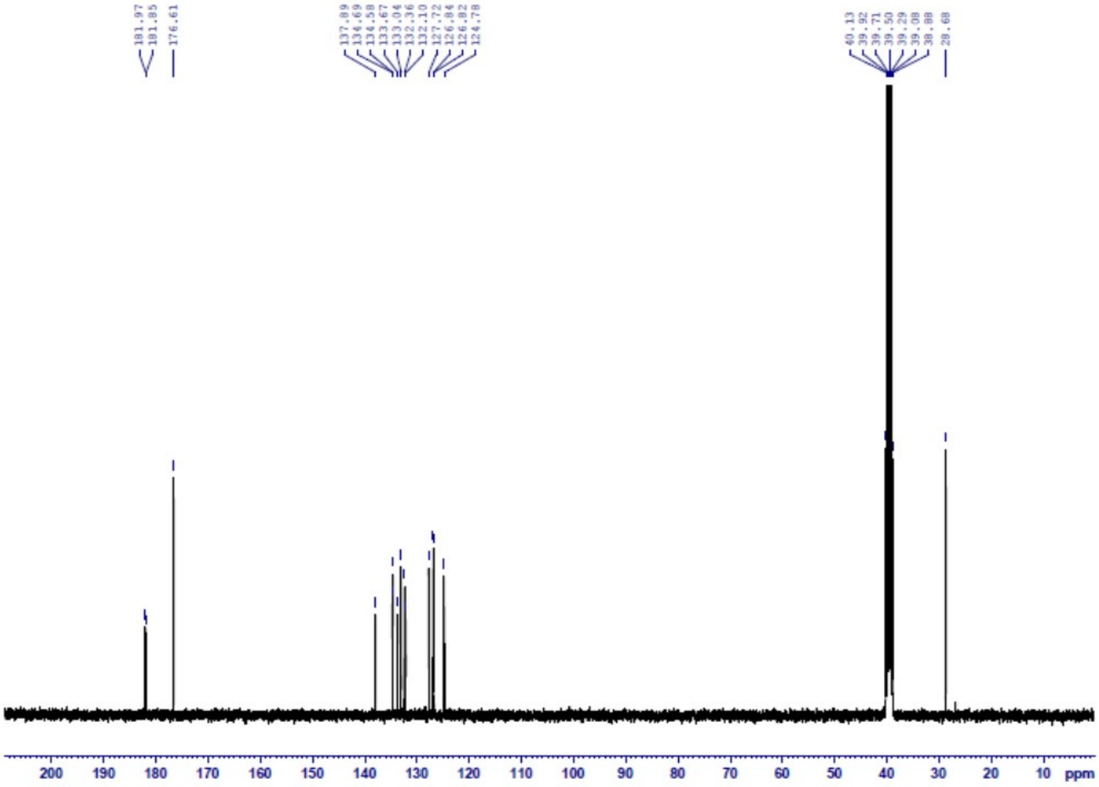

Figure 47. ^{13}C NMR spectrum of **15**.

N^1-(2-aminoethyl)-N^4-(9,10-dioxo-9,10-dihydroanthracen-2-yl)succinamide (16)

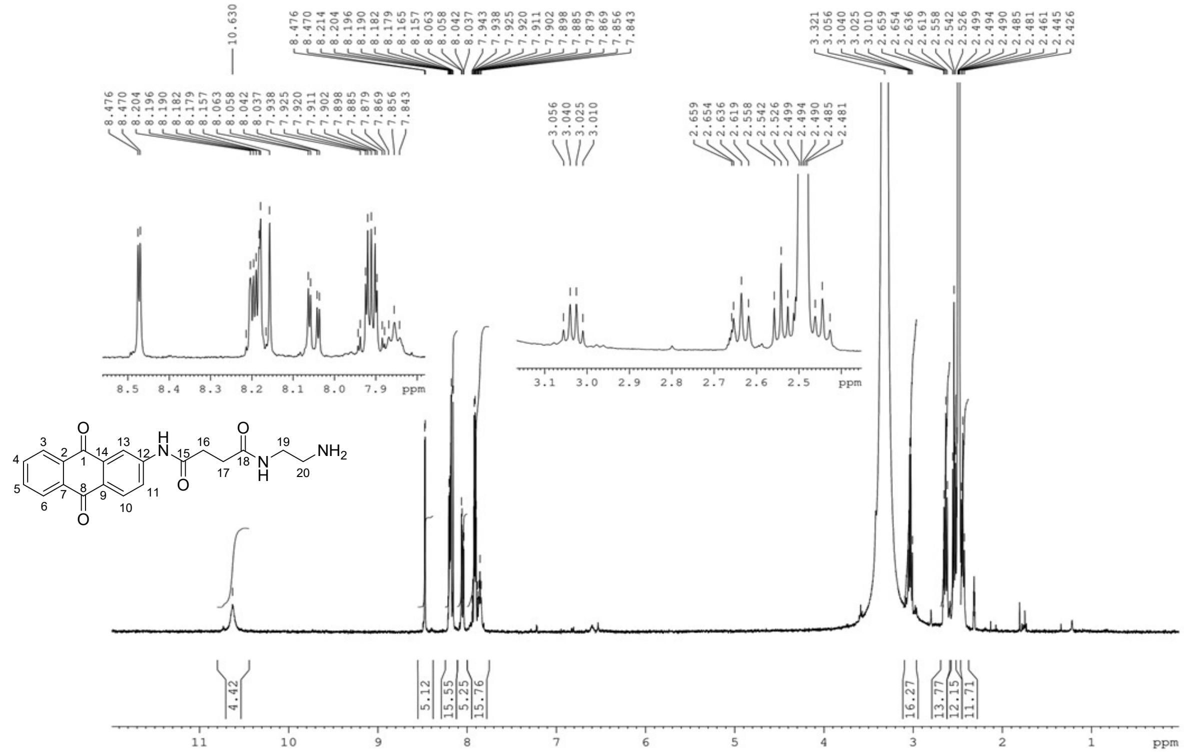

Figure 48. ^{1}H NMR spectrum of **16**.

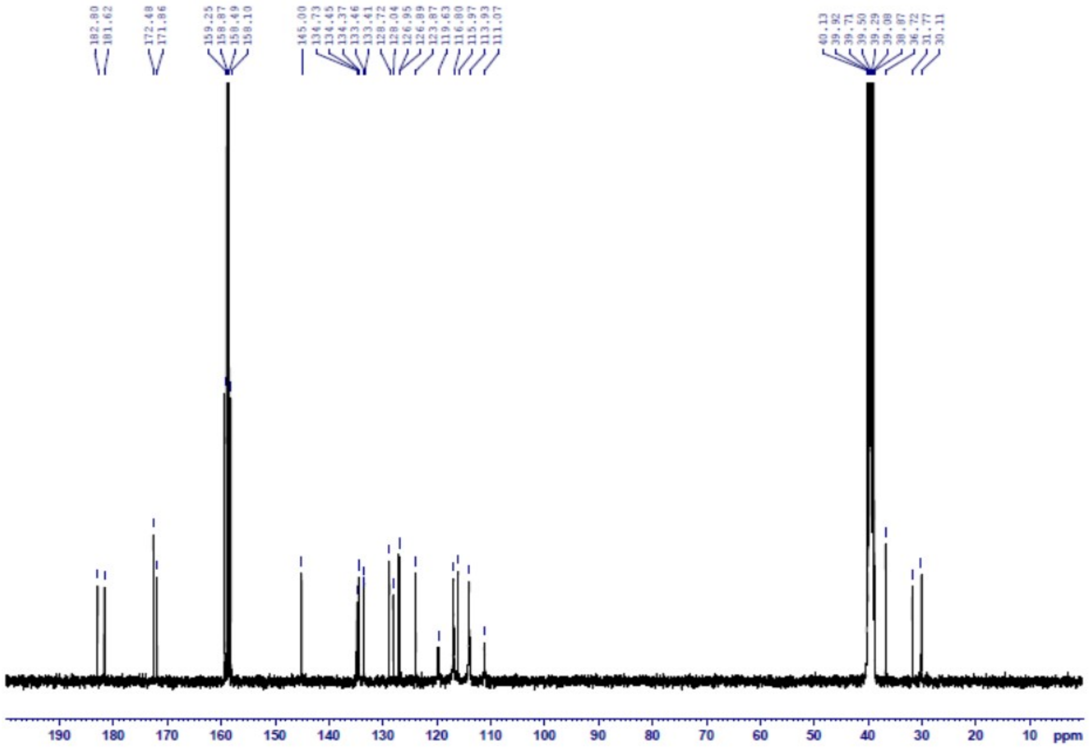

Figure 49. ^{13}C NMR spectrum of **16**.

N^1-(3-aminopropyl)-N^4-(9,10-dioxo-9,10-dihydroanthracen-2-yl)succinamide (17)

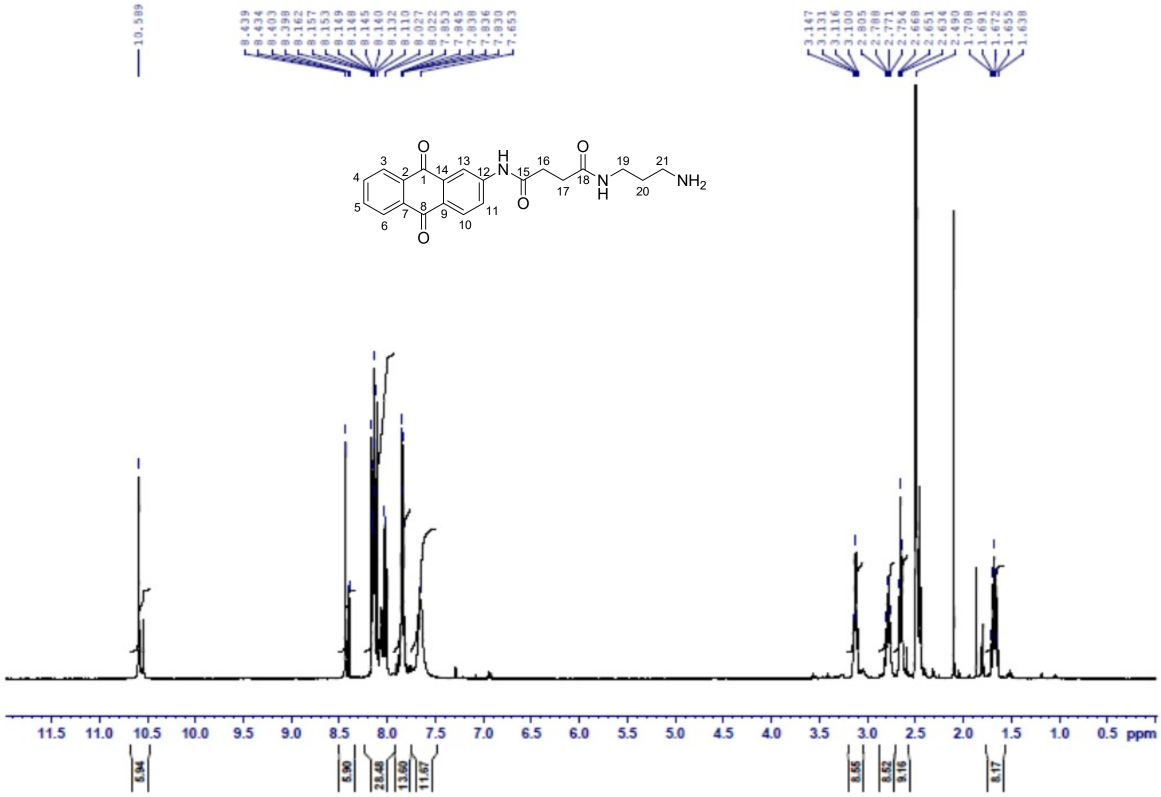

Figure 50. ^{1}H NMR spectrum of **17**.

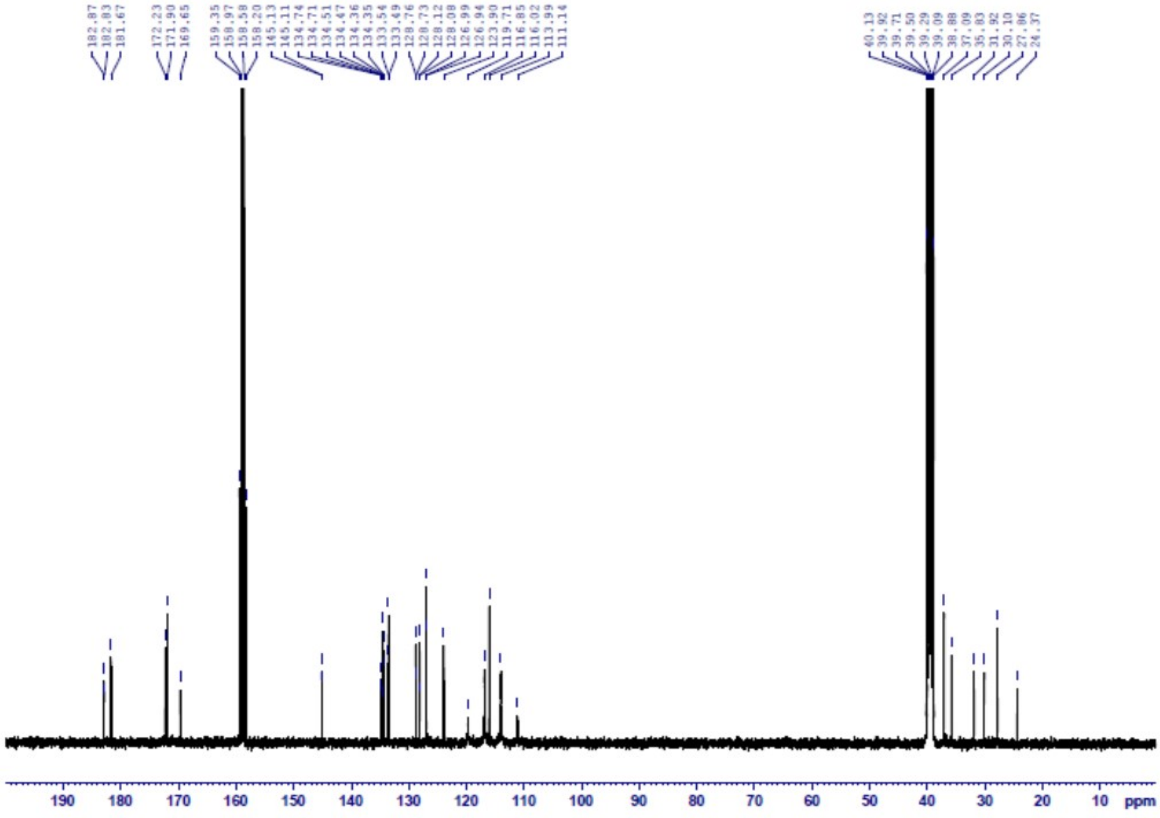

Figure 51. ^{13}C NMR spectrum of **17**.

N[1],N[1′]-(((2,2'-(1,3,8,10-tetraoxoanthra[2,1,9-def:6,5,10-d'e'f']diisoquinoline-2,9(1*H*,3*H*,8*H*,10*H*)-diyl)bis(3-phenylpropanoyl))bis(azanediyl))bis(ethane-2,1-diyl))bis(*N*4-(9,10-dioxo-9,10-dihydroanthracen-2-yl)succinamide) (18)**

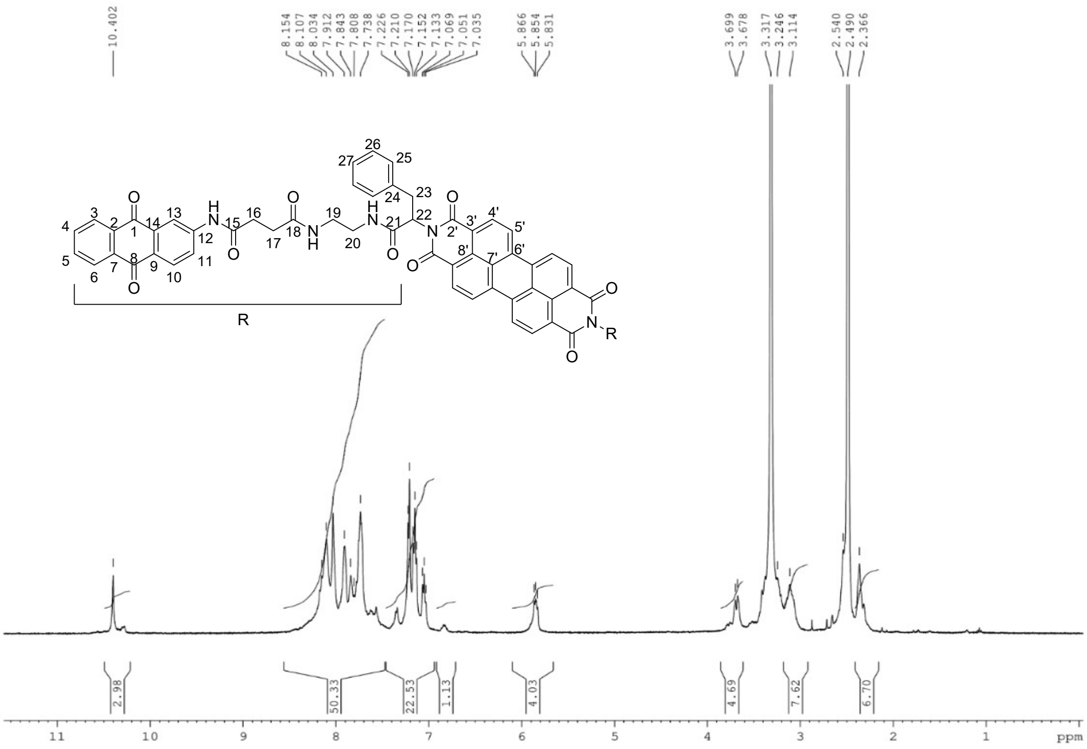

Figure 52. ^{1}H NMR spectrum of **18**.

***N*[1],*N*[1′]-(((2,2'-(1,3,8,10-tetraoxoanthra[2,1,9-def:6,5,10-d'e'f']diisoquinoline-2,9(1*H*,3*H*,8*H*,10*H*)-diyl)bis(3-phenylpropanoyl))bis(azanediyl))bis(propane-3,1-diyl))bis(*N*4-(9,10-dioxo-9,10-dihydroanthracen-2-yl)succinamide) (19)**

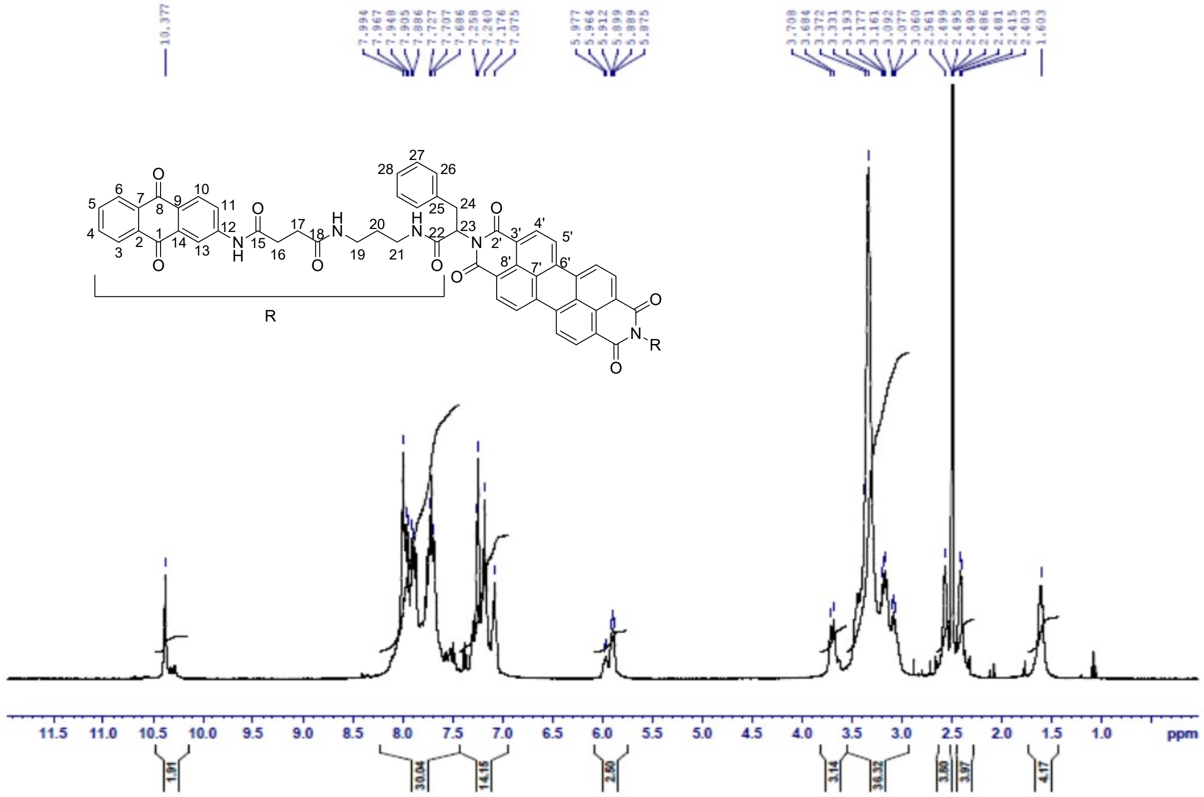

Figure 53. ^{1}H NMR spectrum of **19**.

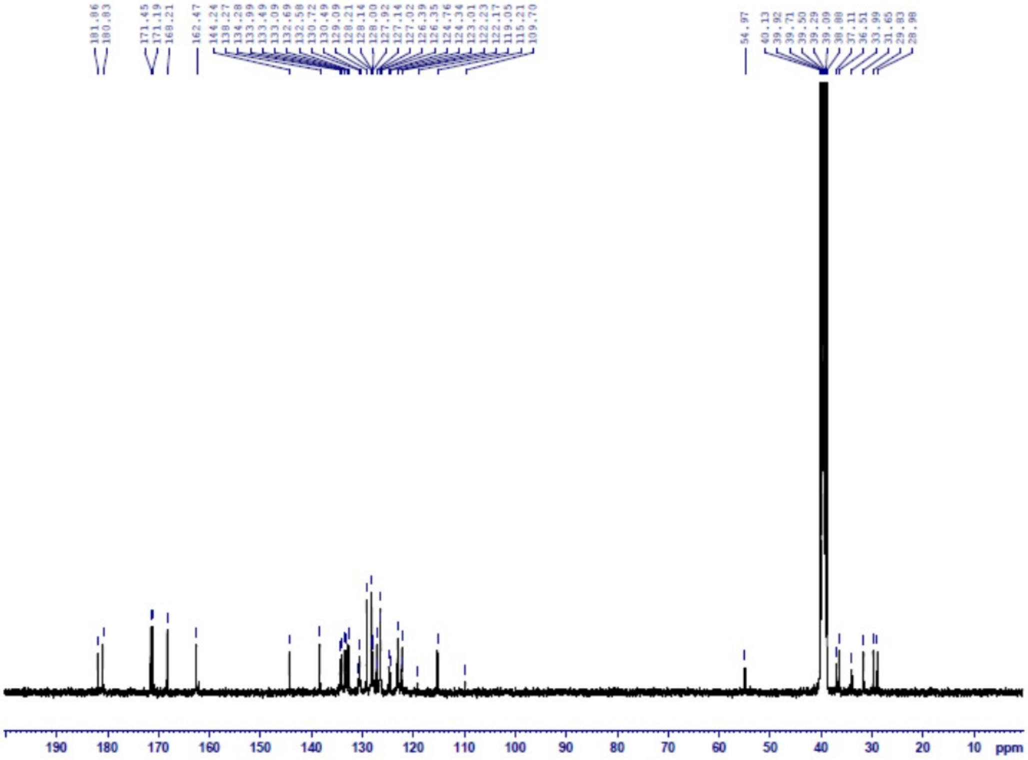

Figure 54. ^{13}C NMR spectrum of **19**.

XI. Evolution of S-trityl cysteine

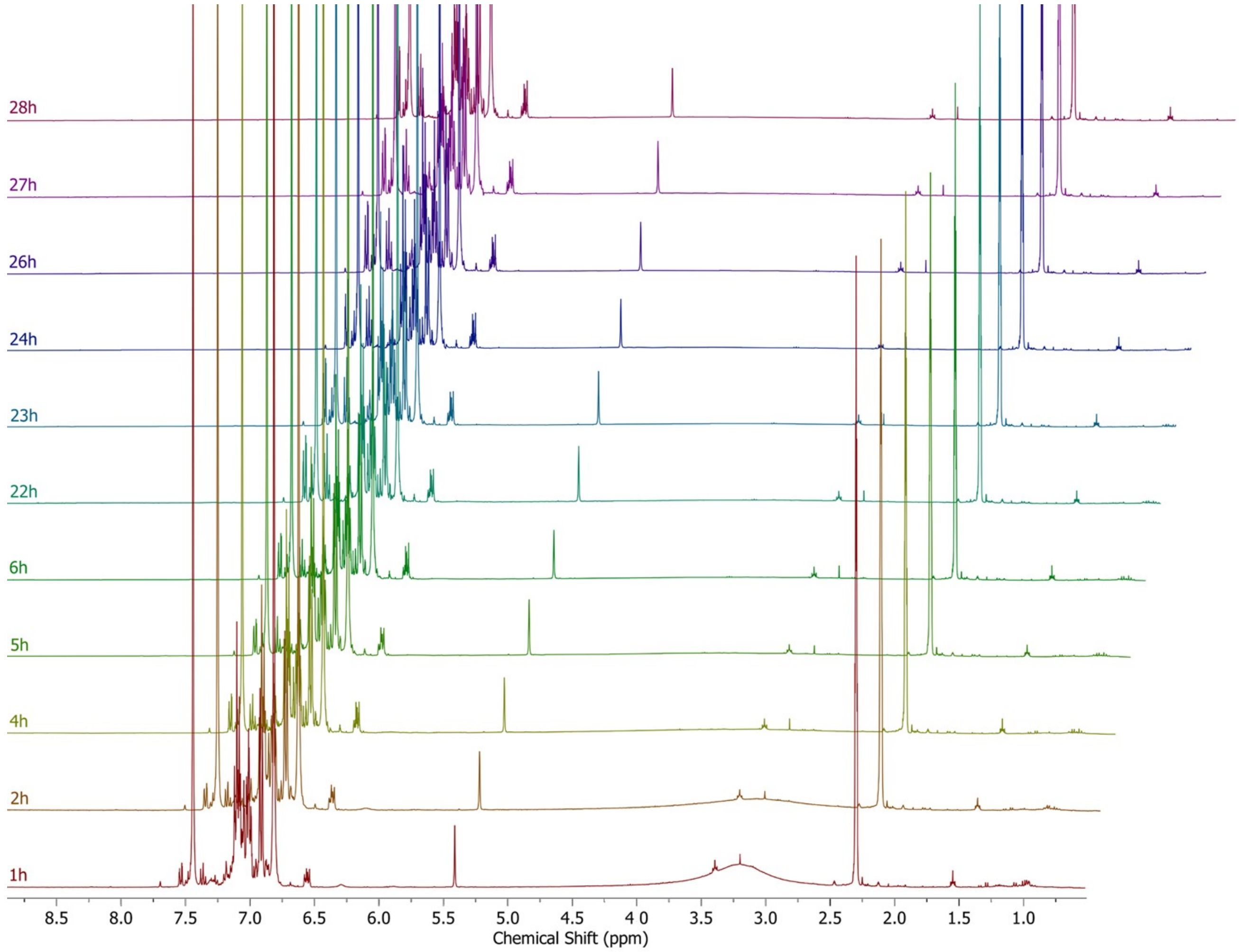

Figure 55. S-trityl cysteine (6.6 mg) was combined with imidazole (8 molar equivalents) in DMSO-d6 (600 μL). The NMR tube was heated at 110 ºC and the reaction was followed at regular intervals for 28h.

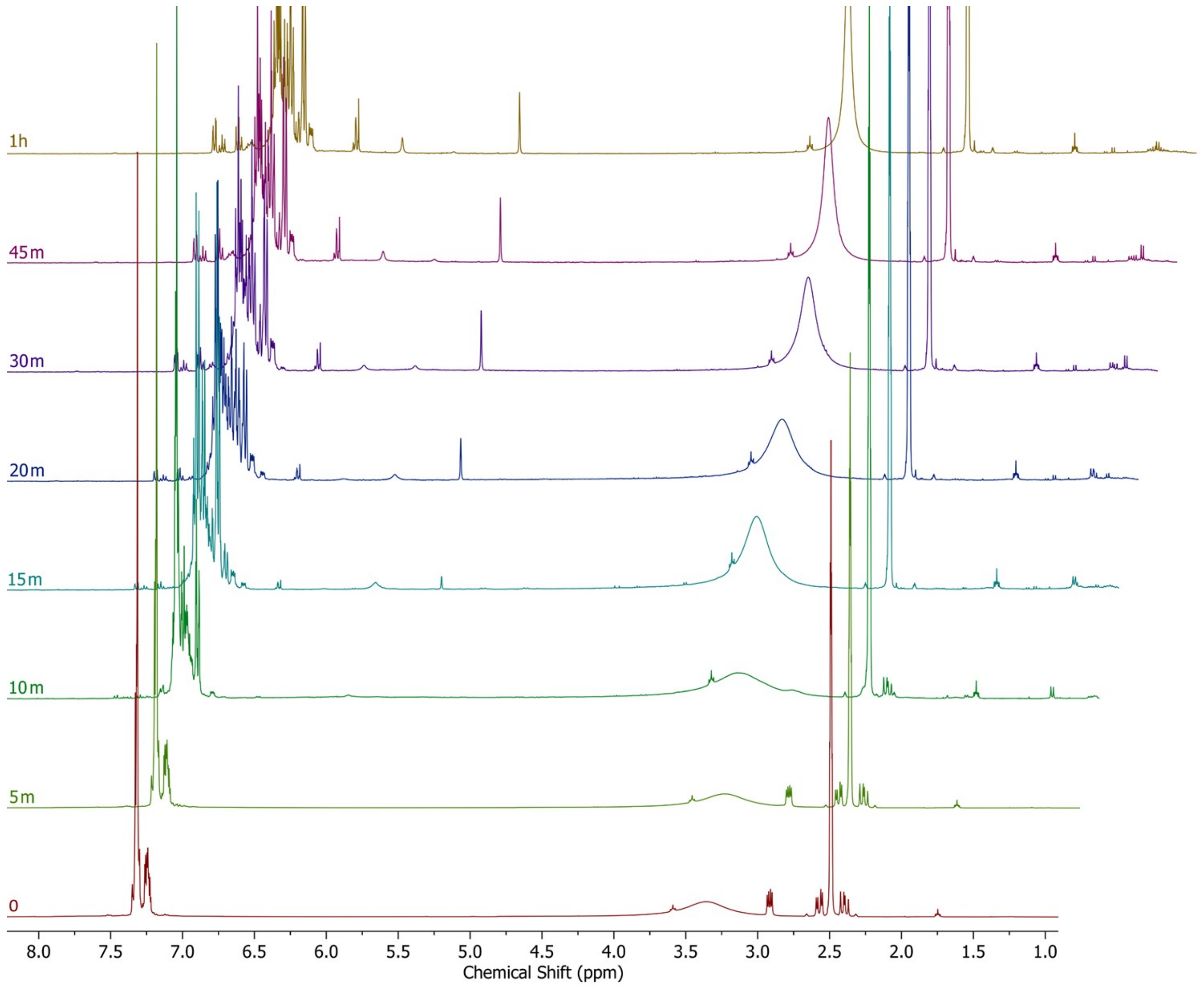

Figure 56. S-trityl cysteine (6.6 mg) was heated in DMSO-d6 (600 μL) at 110 ºC for 1 h, in an NMR tube and the spectrum was registered at regular intervals.

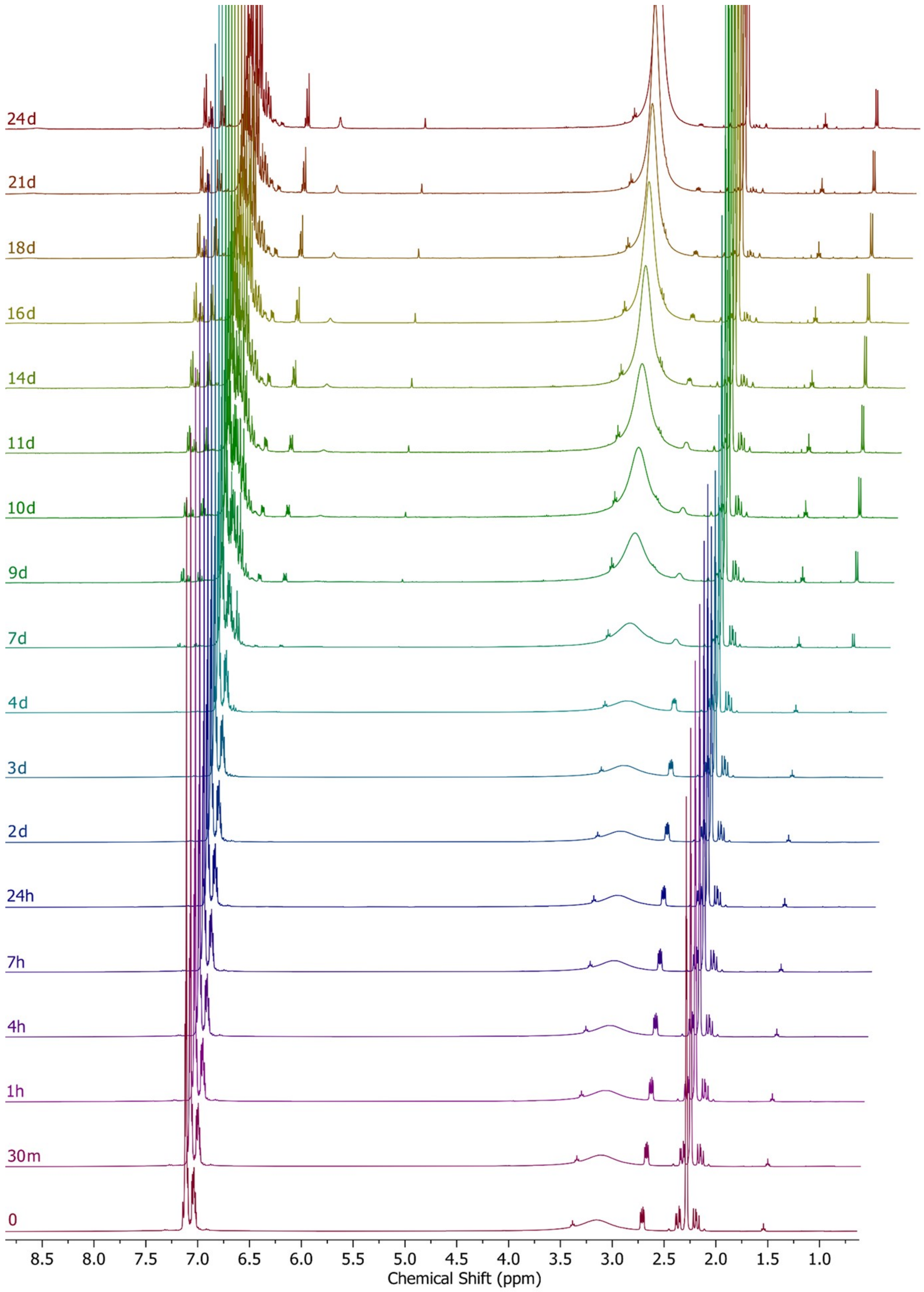

Figure 57. S-trityl cysteine (6.6 mg) was solubilized in DMSO-d6 (600 μL) and the solution was kept at room temperature (20-22 ºC) inside an NMR tube. The spectrum was registered at regular intervals for 24 days.

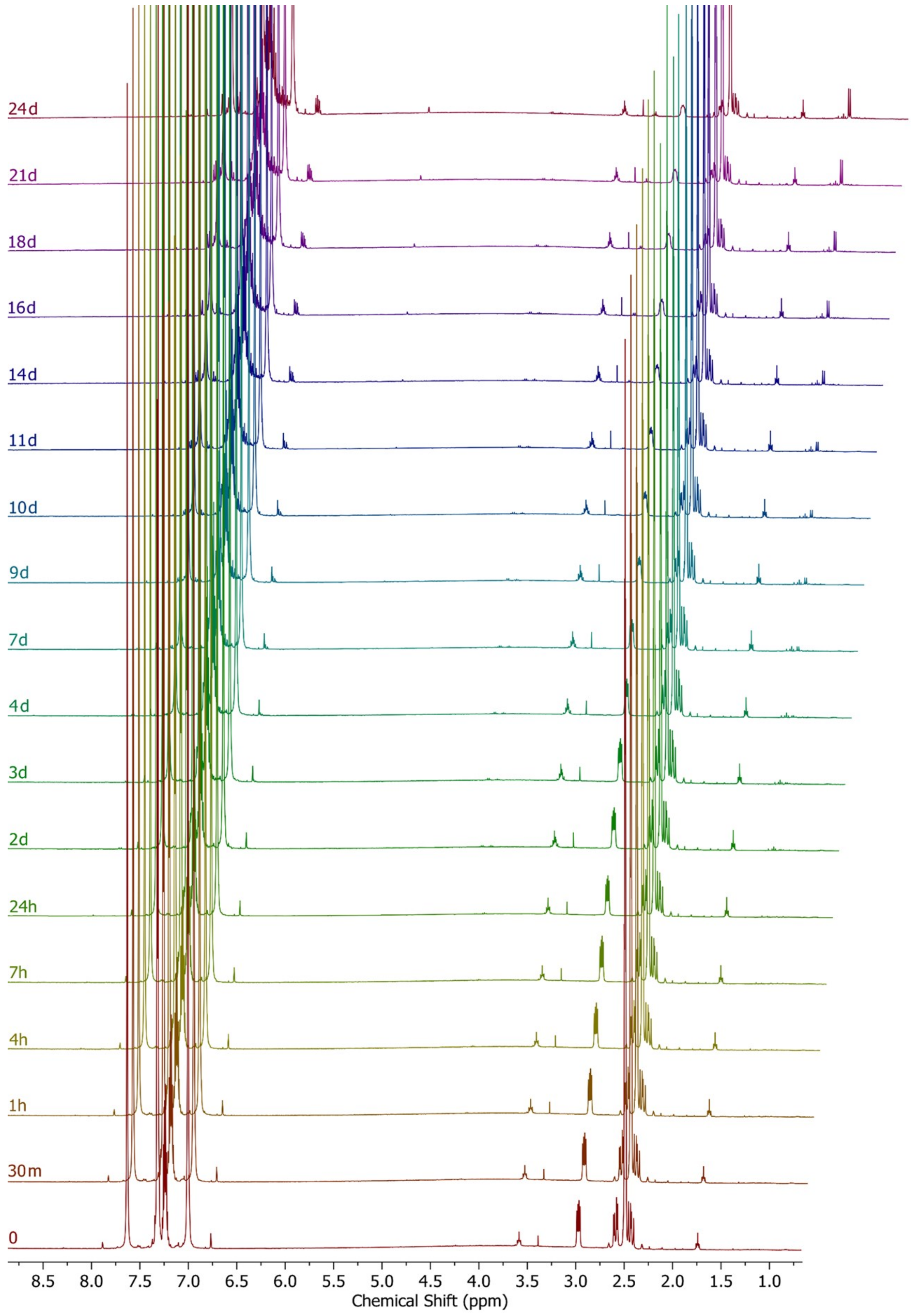

Figure 58. S-trityl cysteine (6.6 mg) was combined with imidazole (8 molar equivalents) in DMSO-d6 (600 μL). The NMR tube was kept at room temperature (20-22 ºC) and the reaction was followed at regular intervals for 24 days.

XII. References

1. R. F. Araújo, C. J. R. Silva, M. C. Paiva, M. M. Franco, M. F. Proença, Efficient dispersion of multi-walled carbon nanotubes in aqueous solution by non-covalent interaction with perylene bisimides, *RSC Adv*., 2013, **3**, 24535-24542.
